# Proteomics reveals changes in hepatic proteins during chicken embryonic development: an alternative model to study human obesity

**DOI:** 10.1186/s12864-017-4427-6

**Published:** 2018-01-08

**Authors:** Mengling Peng, Shengnan Li, Qianian He, Jinlong Zhao, Longlong Li, Haitian Ma

**Affiliations:** Key Laboratory of Animal Physiology and Biochemistry, College of Veterinary Medicine, Nanjing Agricultural University, Nanjing, 210095 China

**Keywords:** iTRAQ, Chicken embryo, Model, Obesity, Proteomics

## Abstract

**Background:**

Chicken embryos are widely used as a model for studies of obesity; however, no detailed information is available about the dynamic changes of proteins during the regulation of adipose biology and metabolism. Thus, the present study used an isobaric tags for relative and absolute quantitation (iTRAQ)-based proteomic approach to identify the changes in protein abundance at different stages of chicken embryonic development.

**Results:**

In this study, the abundances of 293 hepatic proteins in 19-day old of chicken embryos compared with 14-day old and 160 hepatic proteins at hatching compared with 19-day old embryos were significantly changed. Pathway analysis showed that fatty acid degradation (upregulated ACAA2, CPT1A, and ACOX1), protein folding (upregulated PDIs, CALR3, LMAN1, and UBQLN1) and gluconeogenesis (upregulated ACSS1, AKR1A1, ALDH3A2, ALDH7A1, and FBP2) were enhanced from embryonic day 14 (E14) to E19 of chicken embryo development. Analysis of the differentially abundant proteins indicated that glycolysis was not the main way to produce energy from E19 to hatching day during chicken embryo development. In addition, purine metabolism was enhanced, as deduced from increased IMPDH2, NT5C, PGM2, and XDH abundances, and the decrease of growth rate could be overcome by increasing the abundance of ribosomal proteins from E19 to the hatching day.

**Conclusion:**

The levels of certain proteins were coordinated with each other to regulate the changes in metabolic pathways to satisfy the requirement for growth and development at different stages of chicken embryo development. Importantly, ACAA2, CPT1A, and ACOX1 might be key factors to control fat deposition during chicken embryonic development. These results provided information showing that chicken is a useful model to further investigate the mechanism of obesity and insulin resistance in humans.

**Electronic supplementary material:**

The online version of this article (10.1186/s12864-017-4427-6) contains supplementary material, which is available to authorized users.

## Background

The world is in health transition, and obesity is the greatest threat to human health, obesity is associated with the development of diabetes and alterations typical of metabolic syndrome [1]. Many studies have shown that obesity is associated with various lifestyle-related diseases, such as cardiovascular, chronic diabetic hyperglycemia, diabetes mellitus, hypertension, and fatty liver disease [2–4], causing a major health burden in terms of morbidity and mortality. It is often considered that obesity mainly occurs in developed countries; however, it has spread from developed countries to less-wealthy countries over the past decade. The biology of obesity is very complex, and the mechanisms linking obesity to various diseases are still poorly understood [5].

Various models have been used to study the biology of obesity in humans, including fetuses and children [6, 7], rat embryos or pups [8], and chicken embryos [9]. Recently, chickens have become a widely used model to study adipose tissue biology, metabolism, and obesity because their genetic makeup is approximately 70% homologous to that of humans [10]. Like humans, the liver, rather than adipose tissue, is the major site for de novo lipid synthesis in chickens [11]. Quantitative trait loci linked to fatness in chickens contain genes implicated in human susceptibility to obesity and diabetes [12]. It was reported that chickens mimic the early stage of type 2 diabetes in humans, exhibiting both hyperglycemia and resistance to exogenous insulin [13]. In addition, chickens also represent a model to study the mechanisms of adipocyte hyperplasia during development, a process that may exacerbate adult obesity [13]. However, relatively little is known about the dynamic changes of key proteins that regulating adipose metabolism at the different stages of chicken embryonic development.

It is widely accepted that during early life, maternal and environmental factors have distinct impacts on the long-term health of offspring through ‘programming/malprogramming’ of body functions during ‘critical periods’ of perinatal life [14]. However, the basic mechanisms are not fully understood because of the lack of an animal model to decipher singular risk factors, irrespective of potential confounders and variables, which are unavoidable in the complex placental mammalian mother-fetus-environment interaction [15]. It was reported that the physiological development pattern during the late prenatal development of chickens is similar to that in mammalian and human fetuses [16]. Embryonic development is enclosed in an eggshell that is hardly influenced by external factors and is independent from the mother; therefore, the chicken embryo can be used as an excellent model to investigate pre- and perinatal developmental processes [16, 17]. Thus, it would be meaningful and critical to understand the changes in the abundances of the key proteins that control adipose biology and fat metabolism during chicken embryonic development.

In addition, the domestic chicken provides a widespread and relatively inexpensive source of dietary protein for humans. However, commercial broiler chickens rapidly accumulate excess fat as a result of intensive genetic selection for growth [13]. Excessive fat accumulation is an economic and environmental concern for the broiler industry because it reduces feed utilization and causes excessive nitrogen wastage, as well as the negative effect on consumers who are at increased risk of cardiovascular disease and obesity from dietary fat intake. Notably, chicken embryonic development actually relies on two separate phases of lipid metabolism. One takes place in the parent hen before laying the egg, which includes lipid synthesis in the maternal liver and lipid transport for incorporation into the maturing oocyte. The other phase occurs after embryonic day 14 (E14) during the later stage of embryonic development [18]. At this stage, which is the major growth period, the yolk lipids are the main source of energy metabolism in the embryo. Lipids are taken up by the yolk sac membrane (YSM) from the yolk and then transferred into embryonic circulation for growth [19, 20]. One of the features of lipid metabolism in avians is large lipid droplet accumulation in hepatocytes resulting from the large amounts of triacylglycerol delivered to the tissues [18–22]. Thus, it is vital to understand the metabolic changes in the liver that control adipose tissue metabolism, which would both enhance the utility of chickens as a model for human obesity and insulin resistance, and highlight new approaches to reduce fat deposition in commercial chickens.

Therefore, the present study used the isobaric tags for relative and absolute quantitation (iTRAQ)-based proteomic approach to identify the changing patterns of protein abundance during chicken embryonic development. Analysis of global protein abundance will provide new insights into the mechanism of fat deposition during chicken embryonic development, and pave the way for chicken embryo as a model to investigate the mechanism of obesity and insulin resistance in humans. It also will provide information regarding fat deposition control in commercial chickens.

## Methods

### Animal experiment

A total of 200 fertilized eggs from Ross 308 hens were obtained from Jiangsu Wuxi chicken breeding company (Wuxi, China). Each egg was weighed and numbered individually before incubation. Fertilized eggs were placed into an electric forced-draft incubator (Dezhou Keyu incubation equipment Co., Shandong, China) at 37 ± 0.5 °C and 65% relative humidity, with rocking at an angle of 90° at 30 min intervals. The start of the incubation period was referred to “E1” (1-day-old embryos), and post-hatching was termed hatching day 1 (H1). Finally, 60 liver samples of embryos were collected on E14, E19, and H1 respectively. All samples were snap frozen in liquid nitrogen and stored at −80 °C for further analysis.

### Protein extraction, digestion, and iTRAQ labeling

150 mg of liver samples at different stages of embryonic development was dissolved in lysis buffer containing 1 mM phenylmethylsulfonyl fluoride (Bio Basic Inc., Amherst, NY, USA) and 2 mM ethylenediamine tetraacetic acid (Amersco, Burlington, MA, USA), and mixed thoroughly. After incubating on ice for 5 min, 10 mM dithiothreitol (DTT; Amersco, MA, USA) was added to the mixtures and disrupted by tissue lysing machine (240 s, 50 HZ/s. Shanghai Jingxin Industrial Development Co., LTD, Shanghai, China). The mixtures were centrifuged at 25,000×*g* 4 °C for 15 min. The supernatant was resuspended in 10 mM DTT and kept at 56 °C for 60 min to reduce the disulfide bonds of the peptides. Then, 55 mM iodoacetamide (Sigma-Aldrich, Saint Louis, MO, USA) was added to the solution and kept it in a dark room for 45 min, and then mixed with equal volume of cold acetone (Guangdong Shantou Xilong Chemical Co., LTD. Shantou, Guangdong, China), and stored at −20 °C for 2 h. The solution was centrifuged at 25,000×*g* 4 °C for 15 min and the eluate was collected. Then, 1 mL of cold acetone was added and stored at −20 °C for 30 min. The solution was then centrifuged at 25,000×*g* 4 °C for 15 min. The lysate was sonicated with a probe sonicator (Ningbo Xingzhi Biotechnology Co., LTD, Ningbo, Zhejiang, China) for 15 min followed by centrifugation at 25,000×*g* 4 °C for 15 min. The supernatant was collected, and protein concentration was determined using the Bradford method [23]. Ten samples were randomly selected and mixed with equal protein content in each treatment. Finally, six liver extract protein pools were used in iTRAQ protocol at different stage of chicken embryonic development.

For trypsin-mediated protein digestion, 100 μg of protein from different treatment samples was reduced and alkylated, and then digested using trypsin gold (Promega, Madison, USA) with the ratio of protein: trypsin = 20:1 at 37 °C for 4 h. After trypsin digestion, the samples were resolved in 0.5 mM TEAB and labeled with different isobaric tags according to the 8-plex iTRAQ reagent application kit protocol (AB Sciex, Concord, MA, USA). The mixtures of iTRAQ-labeled peptides were pooled and dried by vacuum centrifugation, and then fractionated by reverse phase chromatography.

### Fractionation by reverse phase chromatography

To fractionate iTRAQ-labeled peptide mixture using the Shimadzu LC-20AB HPLC Pump system, the mixture was reconstituted with buffer A [5% ACN (Thermo Fisher Scientific, Waltham, MA, USA), 95% H_2_O, with the pH adjusted to 9.8 with 2 mL of ammonia (Sangon Biotech Co., LTD, Shanghai, China)] and loaded onto a 4.6 × 250 mm Gemini C18 column containing 5 μm particles (Phenomenex, Torrance, CA, USA). The peptides were eluted at a flow rate of 1 mL/min with a gradient of 5% buffer B (5% H_2_O, 95% ACN, pH adjusted to 9.8 with ammonia) for 10 min, 5–35% buffer B for 40 min, and 35–95% buffer B for 1 min. The system was then maintained in 95% buffer B for 3 min and decreased to 5% within 1 min before equilibrating with 5% buffer B for 10 min before the next injection. Elution was monitored by measuring absorbance at 214 nm, and fractions were collected every 1 min. The eluted peptides were pooled as 20 fractions and vacuum-dried.

### LC-ESI-MS/MS analysis

Each fraction was re-suspended in buffer A (5% ACN, 0.1% FA) and centrifuged at 20,000×*g* for 10 min. In each fraction, the final concentration of peptides was about 0.5 μg/μL. The supernatant was loaded onto a LC-20 AD Nanoacquity HPLC (Shimadzu, Kyoto, Japan) with an autosampler onto a C18 trap column (Waters, Milford, MA, USA), and the peptides were eluted onto an analytical C18 column with a 75 μm inner diameter (Waters, Milford, MA, USA). The samples were loaded at 8 μL/min for 4 min, continued by a 41 min gradient running at 300 nL/min from 5 to 35% B (95% ACN, 0.1% FA), followed by a 5 min linear gradient to 80% buffer B, maintained at 80% for 5 min, and finally returned to 5% in 1 min.

Data acquisition was performed with a TripleTOF 5600 System fitted with a Nanospray III source (AB SCIEX, Concord, Ontario, Canada), a pulled quartz tip as the emitter (New Objectives, Woburn, MA, USA) and controlled with software Analyst 1.6 (AB SCIEX, Concord, Ontario, Canada). Data were acquired under the following MS conditions: ion spray voltage 2.5 kV, a curtain gas at 30 psi, a nebulizer gas at 15 psi, and an interface heater temperature of 150 °C. For information dependent data acquisition (IDA), survey scans were acquired in 250 ms intervals. As many as 30 product ion scans were collected if they exceeded a threshold of 120 counts per second (counts/s) and had a 2+ to 5+ charge-state. Total cycle time was fixed to 3.3 s. The Q2 transmission window was 100 Da for 100%. Four-time bins were summed for each scan at a pulser frequency value of 11 kHz by monitoring the 40 GHz multichannel TDC detector with four-anode channel detect ion. An iTRAQ adjust rolling collision energy was applied to all precursor ions for collision-induced dissociation. Dynamic exclusion was set for 1/2 of peak width (15 s), and then the precursor was refreshed off the exclusion list.

### iTRAQ protein identification and quantification

The raw data files acquire from the Orbitrap were converted into MAS-COT generic format (*.MGF) files using Proteome Wizard. Protein identification was performed using the Mascot search engine (Matrix science, London, UK; version 2.3.02) against the non-redundant and Swiss-Prot databases. Peptides with significance scores ≥20 at the 95% confidence were counted as identified to reduce the probability of false peptide identification. Each confident protein identification involved at least one unique peptide, and for protein species quantitation, a protein species was required to contain at least two unique spectra. The quantitative protein ratios were weighted and normalized by the median ratio in Mascot. The *t*-test was employed and a *P*-value was calculated for each protein. Only ratios with *P*-values <0.05, and fold change ≥1.2 or ≤0.85 were considered significant.

### Bioinformatic analysis

Gene Ontology (GO) annotation analysis including biological process (BP), cellular component (CC) and molecular function (MF) was performed based on the obtaining of differentially abundant proteins. The KEGG database (http://www.genome.jp/kegg/) was used to classify the identified proteins. The Search Tool for the Retrieval of Interacting Genes/Proteins (STRING) database of physical and functional interactions was used to analyze the protein-protein interaction (PPI) of all the identified proteins.

## Results

### Protein identification

**Fig. 1:**
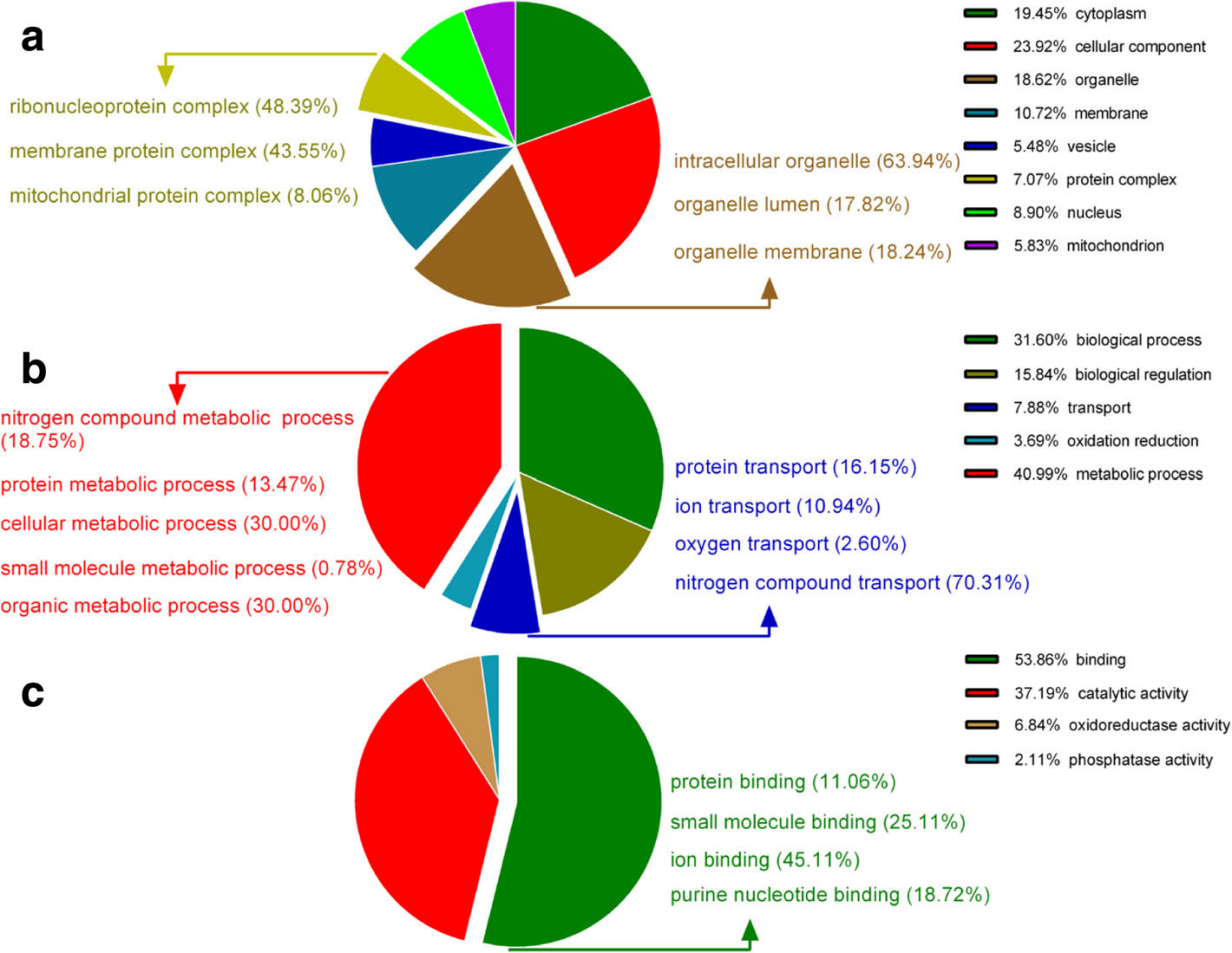
Gene ontology (GO) annotation of all identified proteins by iTRAQ proteomics. (**a**): Cellular components annotation; (**b**): Biological processes annotation; (**c**): Molecular functions annotation

In the present study, a total of 3409 proteins were identified at different stages of chicken embryonic development. The spectra, unique spectra, peptides, and unique peptides numbers, and the mass and sequence coverage of the proteins identified are supplied as Additional file 1: Fig. S1. GO cellular component annotation of all identified proteins showed that the most representative proteins were classified into cytoplasm (19.45%), cellular component (23.92%), and organelle (18.62%) (Fig. 1a). The most represented GO biological process annotations were biological process (31.60%), metabolic process (40.99%), biological regulation (15.84%), and transport (7.88%). Among these biological process, the metabolic process were mainly involved into cellular metabolic process, organic metabolic process, nitrogen compound metabolic process, protein metabolic process, and small molecule metabolic process; the transport encompasses protein transport, ion transport, oxygen transport and nitrogen compound transport (Fig. 1b). GO molecular function annotations were binding (53.86%), catalytic activity (37.19%), oxidoreductase activity (6.84%), and phosphatase activity (2.11%), and binding mainly included protein binding, small molecule binding, ion binding, and purine nucleotide binding (Fig. 1c).

### Differentially abundant proteins

**Fig. 2:**
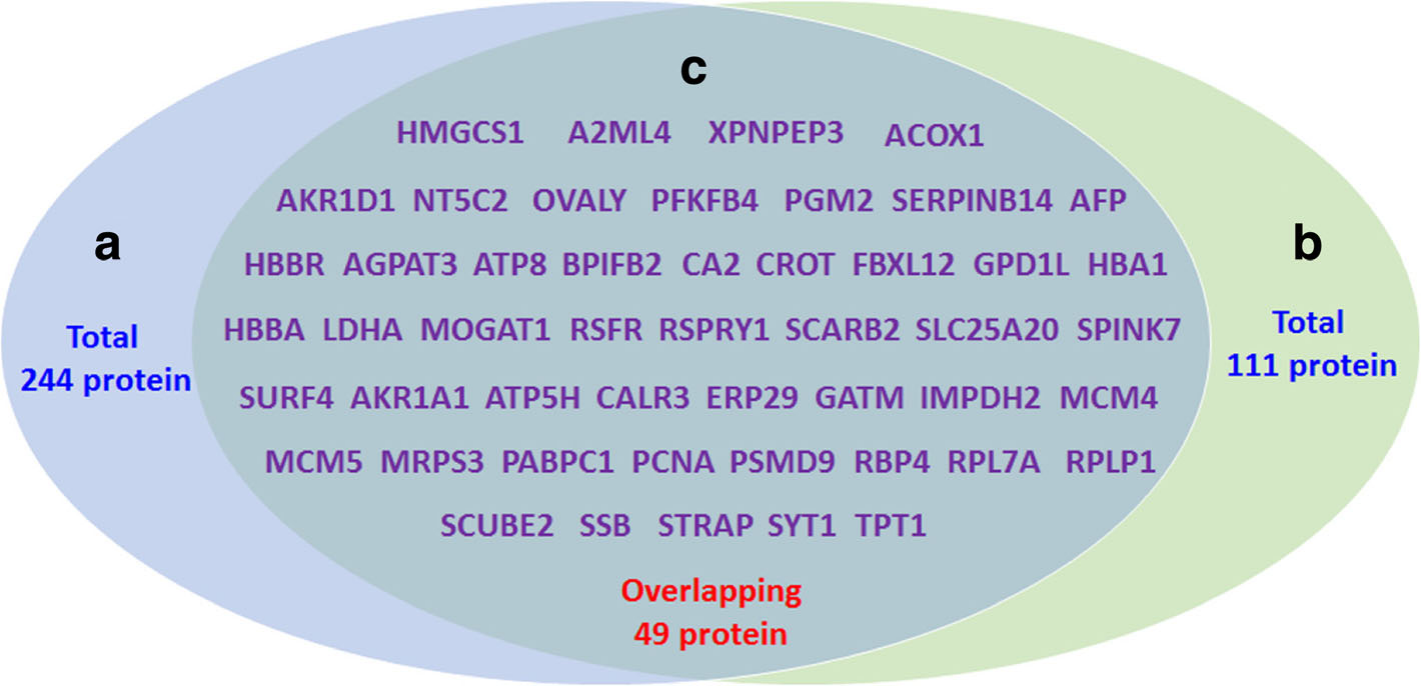
The number of overlapping proteins that were differentially abundant between both comparisons in chicken embryos. **a**: The number of differentially abundant proteins at E19 compared with E14; **b**: The number of differentially abundant proteins at H1 compared with E19; **c**: The number of overlapping proteins differentially abundant between both comparisons

**Fig. 3:**
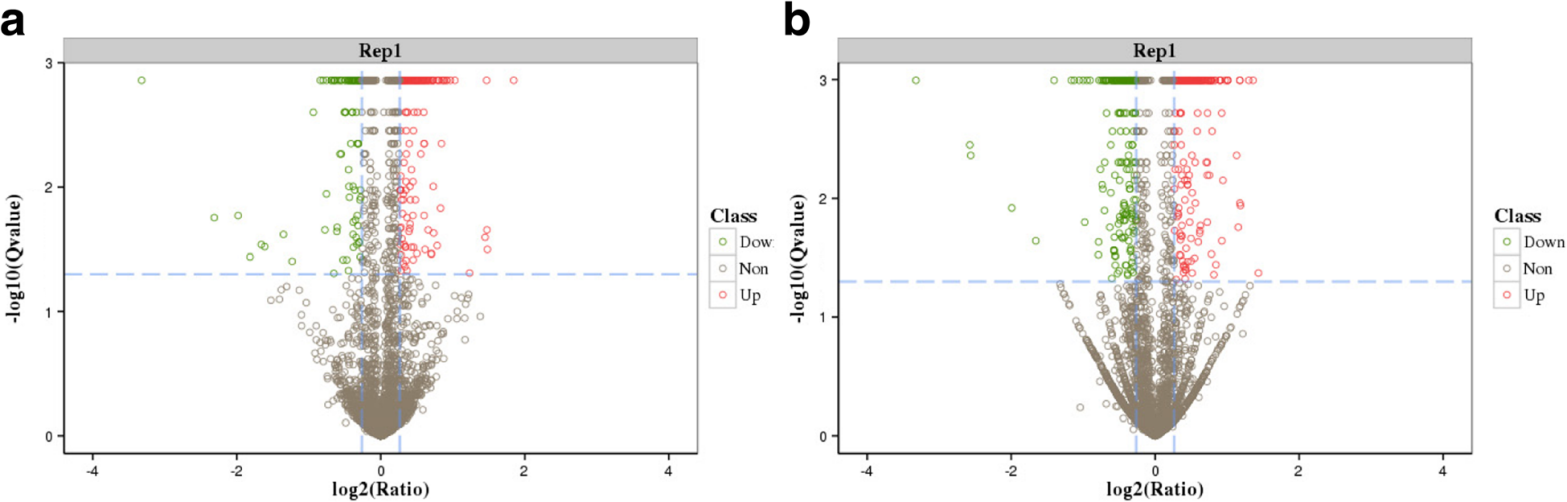
Volcano plot of differentially abundant proteins. **a**: Differentially abundant proteins at E19 compared with E14; (**b)**: differentially abundant proteins at H1 compared with E19. This is a volcano plot of log2 fold-change (x-axis) versus −log10 Q-value (y-axis, representing the probability that the protein is differentially abundant). Q value ≤0.05 and Fold-change ≥1.2 were set as the significance threshold for differential abundance. The red and green dots indicate points-of-interest that display both large-magnitude fold-changes as well as high statistical significance. Dots in red mean significantly upregulated proteins that passed screening threshold. Dots in green mean significant downregulated proteins that passed screening threshold. Gray dots are non-significant differentially abundant protein

We identified 293 significantly differentially abundant hepatic proteins (125 downregulated and 168 upregulated) in the comparison of E19 with E14 in chicken embryos. Detailed information on the differentially abundant proteins is shown in Additional file 2: Table S1. Meanwhile, 160 significantly differentially abundant hepatic proteins (57 downregulated and 103 upregulated) were identified in the comparison of E19 with H1 in chicken embryos (for the details see, Additional file 3: Table S2). Among the differentially abundant proteins, 49 proteins overlapped in both comparisons in chicken embryos (for the details, see Additional file 4: Table S3) and a Venn diagram for the differentially abundant proteins is shown in Fig. 2. In addition, the top 15 differentially abundant proteins in these two comparisons are listed in Table 1. A volcano plot of geometric mean expression ratios and combined *Q*-values of the identified proteins (each protein shown as a circle) shows that the abundances of a number of hepatic proteins were altered (Fig. 3). The cut off values for significant changes in protein ratio ≥ 1.2 or ≤0.8 and that *Q*-value ≤0.05 are indicated by broken blue lines. The unique proteins whose abundance was significantly altered between E14 and E19 or between E19 and H1 were used for further analyses of functional ontology.

**Table 1:** The top 15 proteins with the highest differential abundance in chicken embryos

E19d VS E14d				H1d VS E19d			
Gene Ontology	NCBInr Description	Ratio	*P*-value	Gene Ontology	NCBInr Description	Ratio	*P*-value
ACAD11	acyl-CoA dehydrogenase family member 11	2.703	0.042	LBFABP	fatty acid-binding protein, liver	3.597	0.001
GAL2	Gal 2	2.263	0.011	PPP6R3	serine/threonine-protein phosphatase 6 regulatory subunit 3	2.791	0.032
DM5L	dimethylaniline monooxygenase	2.226	0.018	TEKT4	tektin-4	2.779	0.022
HNMT	histamine N-methyltransferase	2.192	0.004	FABP1	fatty acid-binding protein, liver	2.769	0.001
FBXL12	hepatic lectin	2.011	0.001	IGLL1	Ig light chain precursor	1.954	0.001
HMGCS1	hydroxymethylglutaryl-CoA synthase, cytoplasmic	2.011	0.001	1 SV	Apovitellenin-1	1.89	0.001
Comtd1	catechol O-methyltransferase domain-containing protein 1	1.92	0.007	XDH	xanthine dehydrogenase/oxidase	1.88	0.001
DHRS7	dehydrogenase/reductase SDR family member 7	1.879	0.001	PAICS	multifunctional protein ADE2	1.801	0.001
GGACT	gamma-glutamylaminecyclotransferase-like isoform 2	1.864	0.001	XPNPEP3	probable Xaa-Pro aminopeptidase 3	1.794	0.004
RHOT2	mitochondrial Rho GTPase 2	1.861	0.001	DBI	Acyl-CoA-binding protein	1.751	0.001
LECT2	Myeloid protein 1	1.783	0.001	FKBP5	peptidyl-prolyl cis-trans isomerase FKBP5	1.746	0.001
Ephx1	epoxide hydrolase 1-like	1.772	0.001	CYP1A2	cytochrome P450 1A5	1.721	0.03
PPP2R5A	serine/threonine-protein phosphatase 2A 56 kDa regulatory subunit alpha isoform	1.761	0.044	MCM4	DNA replication licensing factor mcm4	1.715	0.001
LOC768709	uncharacterized protein LOC768709	1.731	0.003	IYD	iodotyrosine dehalogenase 1	1.664	0.023
FAAH	fatty-acid amide hydrolase 1	1.725	0.001	NDUFB1	NADH dehydrogenase [ubiquinone] 1 beta subcomplex subunit 1	1.658	0.01

*Abbreviations*: *NCBInr Description* Description of matched accession (NCBInr)

### Functional ontology classification of differentially abundant proteins

**Fig. 4:**
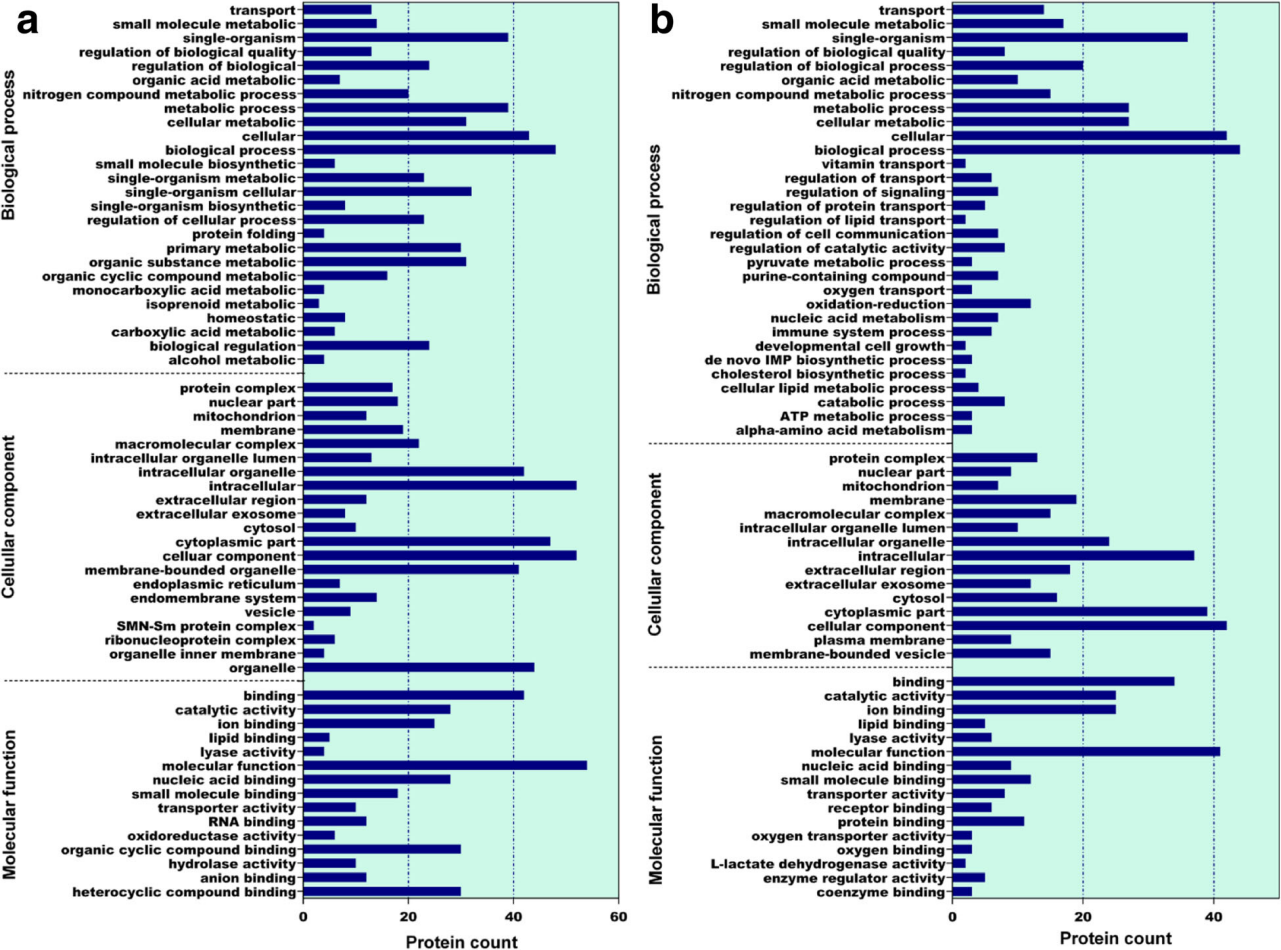
Bar chart of the gene ontology analysis. **a**: Differentially abundant proteins at E19 compared with E14; (**b)**: Differentially abundant proteins at H1 compared with E19.The bar chart shows the distribution of corresponding GO terms. The length shows the number of all differentially abundant proteins associated with the GO term

GO annotation was used to identify the functions of the differentially abundant proteins during chicken embryonic development. Among the 293 differentially abundant proteins between E14 and E19 in chicken embryos, 208 had annotated functions and were classified into 62 functional groups (Fig. 4a), of which the biological process accounted for 26 GO terms (the most representative were biological process and cellular process), cellular component accounted for 21 GO terms (the most representative were cellular component, cytoplasmic part, membrane-bounded organelle, and intracellular), and molecular function accounted for 15 GO terms (the most representative were molecular function and binding). The other 85 proteins had no annotated functions and are shown in Additional file 5: Table S4. Differentially abundant proteins between E14 and E19 in chicken embryos were mainly enriched in metabolic process, biological process, catalytic activity, and binding protein categories.

Among the 160 differentially abundant proteins between E19 and H1 in chicken embryos, 114 had annotated functions and were classified into 62 functional groups (Fig. 4b), of which biological process accounted for 31 GO terms (the most representative were biological process and single-organism), cellular component accounted for 15 GO terms (the most representative were cellular component and cytoplasmic part), and molecular function accounted for 16 GO terms (the most representative were molecular function and binding). The other 46 proteins had no annotated functions and are shown in Additional file 6: Table S5. The differentially abundant proteins between E19 and H1 in chicken embryos were mainly enriched in the small molecule metabolic process, biological process, binding, and catalytic activity.

### Pathway enrichment analysis of differentially abundant proteins

KEGG pathway enrichment analysis showed that the differentially abundant proteins between E14 and E19 in chicken embryos belonged to 21 pathways based on the KEGG database. More detailed information is shown in Additional file 7: Table S6. Among them, metabolic pathways were the most represented pathways, encompassing 56 differentially abundant proteins, followed by glycolysis/gluconeogenesis and fatty acid degradation. Other enriched pathways were valine, leucine, and isoleucine degradation; protein processing in endoplasmic reticulum; pentose phosphate pathway; glycine, serine and threonine metabolism; pyruvate metabolism; butanoate metabolism; arginine and proline metabolism; biosynthesis of amino acids; cysteine and methionine metabolism; ribosome; PPAR signaling pathway; fatty acid metabolism; beta-Alanine metabolism; glycerolipid metabolism; carbon metabolism; purine metabolism; amino sugar and nucleotide sugar metabolism; and oxidative phosphorylation.

At the same time, the differentially abundant proteins between E19 and H1 in chicken embryos mapped to 18 pathways based on the KEGG database. More detailed information is shown in Additional file 8: Table S7. Similarly, metabolic pathways were the most represented pathways encompassing 33 proteins, followed by glycolysis/gluconeogenesis and purine metabolism. Other enriched pathways were ribosome; pentose and glucoronate interconversions; PPAR signaling pathway; galactose metabolism; propanoate metabolism; alanine, aspartate and glutamate metabolism; glycine, serine and threonine metabolism; pyruvate metabolism; amino sugar and nucleotide sugar metabolism; carbon metabolism; protein processing in endoplasmic reticulum; fatty acid metabolism; phenylalanine metabolism; glycerolipid metabolism; and nitrogen metabolism.

### Proteins networks analysis

**Fig. 5:**
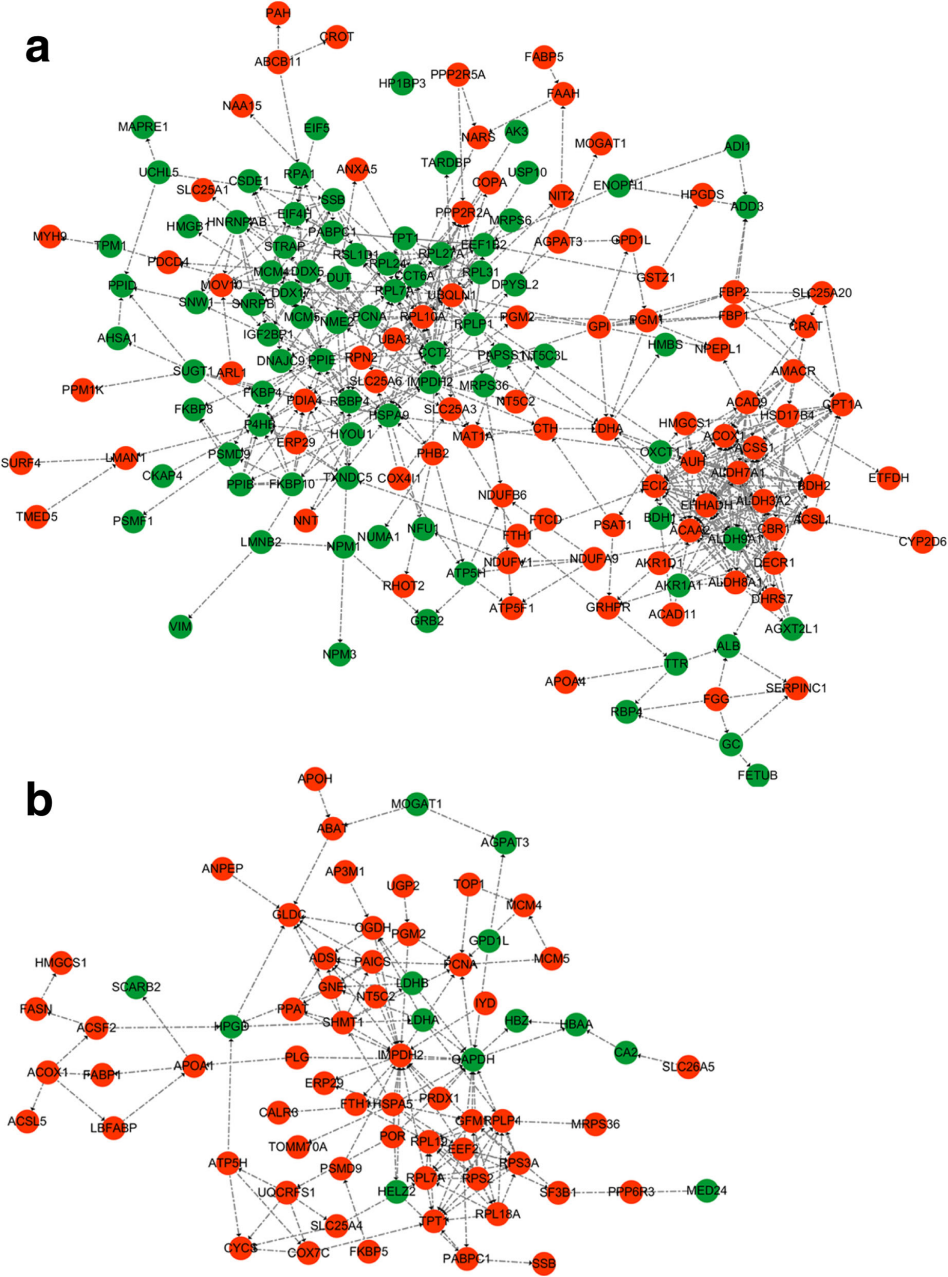
Protein interaction network generated using STRING. (**a**): 168 differentially abundant proteins were observed at E19 compared with E14; (**b**): 70 differentially abundant proteins were observed at H1 compared with E19

The protein-protein interaction networks were generated by the web-tool STRING 9.0 (http://string-db.org). The protein-interactions are shown in Fig. 5, in which the stronger associations are represented by thicker lines. The results showed that functional modules were apparent in the network and formed tight connections with the differential abundant proteins between E14 and E19 in chicken embryos. The functional modules were mainly involved in fatty acid degradation (ACAA2, ACOX1, ACSL1, ALDH3A2, ALDH7A1, ALDH9A1, CPT1A, ECI2, and EHHADH), glycolysis/gluconeogenesis (ACSS1, AKR1A1, ALDH3A2, ALDH7A1, ALDH9A1, FBP1, FBP2, GPI, LDHA, PGM1, and PGM2) and protein processing in the endoplasmic reticulum (CALR3, ERP29, HYOU1, LMAN1, P4HB, PDIA4, RPN2, and UBQLN1). The central functional modules based on the protein-protein interaction networks are shown in Table 2.

**Table 2:** Center differentially expressed proteins at E19d when compared to E14d in chicken embryos

Gene Ontology	NCBInr Description	NCBInr Accession	Species	Uniq_Pep _Num	Uniq_Spec_Num	Protein Coverage	NCBInr Identity	Ratio	*P*-value	Tendency
CALR3	calreticulin	gi|118,103,332	*Gallus gallus*	1	16	0.017	99.26	0.695	0.001	↓
AKR1A1	alcohol dehydrogenase	gi|57,529,654	Gallus gallus	8	24	0.275	100	0.737	0.001	↓
ERP29	endoplasmic reticulum resident protein 29 precursor	gi|444,741,647	Gallus gallus	6	32	0.261	100	0.757	0.001	↓
P4HB	cognin/prolyl-4-hydroxylase/protein disulfide isomerase	gi|21,703,694	Gallus gallus	23	273	0.579	100	0.769	0.001	↓
ALDH9A1	4-trimethylaminobutyraldehyde dehydrogenase	gi|118,094,103	Gallus gallus	14	104	0.373	100	0.783	0.001	↓
HYOU1	hypoxia up-regulated protein 1 precursor	gi|57,528,712	Gallus gallus	19	62	0.306	98.74	0.790	0.001	↓
RPN2	dolichyl-diphosphooligosaccharide--protein glycosyltransferase subunit 2 precursor	gi|57,529,367	Gallus gallus	5	20	0.136	100	1.713	0.001	↑
LDHA	L-lactate dehydrogenase A chain	gi|45,384,208	Gallus gallus	6	27	0.262	100	1.525	0.001	↑
GPI	glucose-6-phosphate isomerase	gi|57,524,920	Gallus gallus	17	46	0.459	100	1.524	0.001	↑
CPT1A	carnitine O-palmitoyltransferase 1, liver isoform	gi|61,097,993	Gallus gallus	15	46	0.234	100	1.517	0.001	↑
EHHADH	peroxisomal bifunctional enzyme	gi|118,094,872	Gallus gallus	27	150	0.496	100	1.506	0.001	↑
ACSS1	acyl-CoA synthetase short-chain family member 1-like	gi|363,734,138	Gallus gallus	12	53	0.299	100	1.471	0.001	↑
FBP2	fructose-1,6-bisphosphatase isozyme 2	gi|50,762,391	Gallus gallus	1	3	0.06	100	1.462	0.032	↑
ACSL1	long-chain-fatty-acid--CoA ligase 1	gi|60,302,804	Gallus gallus	24	107	0.461	100	1.38	0.001	↑
ECI2	enoyl-CoA delta isomerase 2, mitochondrial isoform 2	gi|50,734,079	Gallus gallus	8	29	0.291	99.4	1.345	0.001	↑
FBP1	fructose-1,6-bisphosphatase 1	gi|50,762,393	Gallus gallus	21	414	0.902	95.49	1.337	0.001	↑
UBQLN1	ubiquilin-1 isoform 2	gi|118,104,137	Gallus gallus	2	8	0.082	100	1.335	0.048	↑
LMAN1	protein ERGIC-53 precursor	gi|71,897,199|	Gallus gallus	10	31	0.302	99.79	1.334	0.001	↑
ALDH3A2	fatty aldehyde dehydrogenase	gi|57,525,324	Gallus gallus	13	51	0.371	99.39	1.325	0.001	↑
PGM1	phosphoglucomutase-1	gi|84,619,526	Gallus gallus	8	17	0.136	99.83	1.322	0.001	↑
ALDH7A1	alpha-aminoadipic semialdehyde dehydrogenase isoform 2	gi|118,104,602	Gallus gallus	16	169	0.437	100	1.321	0.001	↑
PDIA4	protein disulfide-isomerase A4	gi|57,530,768	Gallus gallus	28	126	0.592	99.68	1.314	0.001	↑
ACAA2	3-ketoacyl-CoA thiolase, mitochondrial	gi|57,529,492	Gallus gallus	20	140	0.909	100	1.271	0.001	↑
PGM2	phosphoglucomutase-2	gi|71,897,287	Gallus gallus	9	18	0.176	99.84	1.268	0.02	↑
PC	pyruvate carboxylase	gi|45,383,466	Gallus gallus	50	897	0.624	100	1.259	0.001	↑
ACOX1	peroxisomal acyl-coenzyme A oxidase 1	gi|55,741,614	Gallus gallus	15	45	0.393	99.7	1.21	0.003	↑

*Abbreviations*: *NCBInr Identity* Identity score of blast (NCBInr), *NCBInr Accession* Matched accession of blast (NCBInr), *NCBInr Description* Description of matched accession (NCBInr), *Uniq_Pep_Num* Identified unique peptide number of protein, *Uniq_Spec_Num* Identified unique spectrum number of protein

# compared with control group, ↑ indicated up-regulated; ↓ indicated down-regulated

Tendency: proteins expression changes at E19d than that at E14d in chicken embryo, ↑indicated up-regulated; ↓indicated down-regulated

The functional modules were apparent in the network and formed tight connections with the differentially abundant proteins between E19 and H1 in chicken embryos. They were mainly involved in purine metabolism (IMPDH2, NT5C, PGM2, PPAT, and XDH), glycolysis/gluconeogenesis (AKR1A1, GAPDH, LDHA, LDHB, and PGM2), and ribosome (RPL18A, RPL19, RPL7A, RPLP1, RPS2, and RPS3A). The central functional modules based on the protein-protein interaction networks analysis are shown in Table 3.

**Table 3:** Center differentially expressed proteins at H1d when compared to E19d in chicken embryos

Gene Ontology	NCBInr Description	NCBInr Accession	Species	Uniq_Pep _Num	Uniq_Spec_Num	Protein Coverage	NCBInr Identity	Ratio	P-value	Tendency
LDHB	L-lactate dehydrogenase B chain	gi|45,383,766	*Gallus gallus*	21	376	0.859	100	0.684	0.001	↓
GAPDH	glyceraldehyde-3-phosphate dehydrogenase	gi|46,048,961	*Gallus gallus*	22	587	0.999	100	0.766	0.001	↓
LDHA	L-lactate dehydrogenase A chain	gi|45,384,208	*Gallus gallus*	6	27	0.262	99.7	0.727	0.001	↓
XDH	xanthine dehydrogenase/oxidase	gi|46,048,759	*Gallus gallus*	7	10	0.06	100	1.88	0.001	↑
PPAT	amidophosphoribosyltransferase precursor	gi|52,345,390	*Gallus gallus*	15	25	0.38	99.8	1.658	0.001	↑
NT5C2	cytosolic purine 5′-nucleotidase	gi|71,895,075	*Gallus gallus*	9	23	0.225	100	1.369	0.001	↑
PGM2	phosphoglucomutase-2	gi|71,897,287	*Gallus gallus*	9	18	0.176	99.84	1.36	0.001	↑
AKR1A1	alcohol dehydrogenase [NADP(+)]	gi|57,529,654	*Gallus gallus*	8	24	0.275	99.69	1.268	0.001	↑
RPL7A	60S ribosomal protein L7a	gi|52,138,653	*Gallus gallus*	8	19	0.389	100	1.268	0.002	↑
RPL19	60S ribosomal protein L19	gi|71,896,335	*Gallus gallus*	2	7	0.144	100	1.267	0.03	↑
IMPDH2	inosine-5′-monophosphate dehydrogenase 2	gi|71,895,387	*Gallus gallus*	7	11	0.21	100	1.25	0.006	↑
RPS3A	ribosomal protein S3A	gi|129,270,064	*Gallus gallus*	14	67	0.572	100	1.239	0.001	↑
RPLP1	60S acidic ribosomal protein P1	gi|45,384,350	*Gallus gallus*	1	10	0.14	100	1.234	0.016	↑
RPL18A	60S ribosomal protein L18a, partial	gi|363,727,621	*Gallus gallus*	3	6	0.196	100	1.218	0.035	↑
RPS2	40S ribosomal protein S2	gi|461,496,478	*Gallus gallus*	7	20	0.266	100	1.21	0.004	↑

*Abbreviations*: *NCBInr Identity* Identity score of blast (NCBInr), NCBInr Accession, Matched accession of blast (NCBInr), *NCBInr Description* Description of matched accession (NCBInr), *Uniq_Pep_Num* Identified unique peptide number of protein, *Uniq_Spec_Num* Identified unique spectrum number of protein

# compared with control group, ↑ indicated up-regulated; ↓ indicated down-regulated

Tendency: proteins expression changes at H1d than that at E19d in chicken embryo, ↑indicated up-regulated; ↓indicated down-regulated

Meanwhile, the differentially abundant proteins that were not assigned to any known functions in both comparisons are shown in Additional file 9: Table S8 and Additional file 10: Table S9, respectively. 47 and 28 proteins with differential abundance could not be assigned to known functions at different stages of chicken embryonic development. Among them, no anyone abundantly expressed proteins were found at E19 compared with E14 in chicken embryos, while Ig light chain precursor (IGLL1) was one abundantly expressed protein at H1 compared with E19 in chicken embryo.

## Discussion

Chickens are widely used both as a source of dietary protein for humans and a model for studies of obesity or insulin resistance in humans; however, few studies have investigated the dynamic changes in proteins that control adipose biology and metabolism in chicken embryonic development. The present study employed the iTRAQ-based proteomics approach to investigate the changes in global protein abundance and identified 3409 proteins at different stages of chicken embryonic development. GO biological process analysis indicated that most of the identified proteins were related to carbon metabolism, purine metabolism, and fatty acid metabolism during chicken embryonic development. In addition, GO cellular components analysis showed that the differentially abundant proteins were mainly involved in cellular component and organelle at different stages of chicken embryonic development.

The abundance of 293 hepatic proteins was significantly altered at E19 compared with E14 in chicken embryos, and the most representative pathways of these differentially abundant proteins were fatty acid degradation, glycolysis/gluconeogenesis, and protein processing in the endoplasmic reticulum, according to pathway enrichment analysis. Lipid metabolism is a complex process and the key proteins related to this process play important roles in mammal and chicken [24]. During chicken embryonic development, lipids in yolk are the major source of nutrition for growth [20–22]. During the late period of chicken embryonic development, a number of genes are expressed, most of which are involved in lipid metabolism and energy metabolism. A previous study reported that the acetyl-coenzyme A acyltransferase 2 (*ACAA2*) gene showed higher expression (2.02-fold change) in the livers of chicken E16 compared with E20 embryos [19]. Consistently, our data showed that the abundance of ACAA2, a central functional module in fatty acid degradation, was significantly increased at E19 compared with E14 in chicken embryos. ACAA2 mainly catalyzes the last step of the mitochondrial fatty acid β-oxidation. It was reported that ACAA2 decreases fatty acid content through promoting β-oxidation of fatty acids in patients with cancer [25]. ACAA2 is associated with abnormal blood lipid levels and an individual’s risk for coronary artery disease [26, 27]. CPT1A is another central functional module in the fatty acid degradation pathway, and its abundance was significantly increased at E19 compared with E14 in chicken embryos. As one the isoforms of carnitine palmitoyltransferase I (CPT I), abnormal CPT1A expression caused the elevation of free fatty acid levels, accumulation of fat, and decreased oxidation of fatty acid in humans [28]. In addition, our results showed that the abundance of peroxisomal acyl-coenzyme A oxidase 1 (ACOX1) was significantly increased at E19 compared with E14 in chicken embryos. Differential expression of ACOX1 can cause perioral lipid accumulation on top of the initially homogeneous steatosis [29]. Lipid accumulation is considered as a key factor in explaining the epidemic rise in obesity and metabolic syndrome [30]. As the central functional modules in the fat metabolism pathway, the increased abundance of ACAA2, CPT1A and ACOX1 indicated that lipolysis was enhanced to provide energy for growth from E14 to E19 in chicken embryos.

Protein processing in the endoplasmic reticulum was another most representative pathway enriched for differentially abundant proteins between E14 and E19 in chicken embryos. We found that five central functional modules were upregulated (CALR3, LMAN1, PDIA4, RPN2 and UBQLN1) and three central functional modules were downregulated (ERP29, HYOU1 and P4HB). From E14 to E19 during chicken embryonic development, the exocrine pancreas and duodenum begin to mature, the embryo is capable of respiratory movements, and the stomach begins to contract, which requires the involvement of various proteins. Proteins are modified, assembled, and folded in the endoplasmic reticulum (ER) before exerting their functions. In the present study, we found that the levels of protein disulfide-isomerase A4 (PDIA4), protein ERGIC-53 precursor (LMAN1), and ubiquilin-1 (UBQLN1) increased from E14 to E19 in chicken embryos. Upregulation of protein disulfide isomerases (PDIs) in the ER can increase disulfide bond formation efficiency and the protein folding rate [31, 32]. Meanwhile, moving the correctly folded proteins to Golgi apparatus requires the participation of other proteins, such as LMAN1 [33]. UBQLN1 can allow misfolded proteins to be degraded through the proteasomal system to maintain the normal physiology of the organism [34]. In this study, the abundance of dolichyl-diphosphooligosaccharide-protein glycosyltransferase subunit 2 precursor (RPN2) was upregulated, while the abundances of ER resident protein 29 precursor (ERP29) and cognin/prolyl-4-hydroxylase/protein disulfide isomerase (P4HB) were downregulated from E14 to E19 in chicken embryos. RPN2 is a unique integral glycoprotein in the rough ER membrane, and participates in translocation and the maintenance of ER structural integrity [35]. ERP29 mainly plays roles in secretory protein processing, which leads to various transcript variants through alternative splicing [36]. P4HB activity is tightly regulated and enhanced during protein catalysis processes [37]. It was reported that hypoxia up-regulated protein 1 precursor (HYOU1) was accumulated in the ER under hypoxia [38]. As mentioned above, the chicken embryo is capable of respiratory movements at about E19 thus the downregulation of HYOU1 from E14 to E19 in chicken embryos suggested that a physiological state transition occurs chicken embryos begin to have respiratory movements. Taken above, these results indicated that protein synthesis was enhanced from E14 to E19 in chicken embryos, and which may be associated with the chicken embryos requiring more proteins for beak tucking and other organism developments at this stage.

Based on the differentially abundant proteins between E14 and E19 in chicken embryos, the glycolysis/gluconeogenesis metabolism pathways was enriched and 10 central functional modules (ACSS1, AKR1A1, ALDH3A2, ALDH7A1, ALDH9A1, FBP1, FBP2, GPI, LDHA, and PGM2) associated with this pathway were significantly altered. ACSS1, a mitochondrial isoform of acetyl-CoA short chain synthetase (ACSSs), plays a key role in energy metabolism [39]. Acetyl-CoA in mitochondria can be produced by ACSS1 and then exported to the cytosol for fatty acid synthesis in mammals [40]. As discussed above, fatty acid degradation is promoted by upregulating ACAA2, CPT1A, and ACOX1 levels from E14 to E19 in chicken embryos. Thus, acetyl-CoA, produced by enhanced levels of ACSS1, might enter into the citric acid cycle and produce energy to conserve the consumption of glucose at this stage of chicken embryo development. Alcohol dehydrogenase [NADP+] (AKR1A1) is associated with increased glycolysis/gluconeogenesis [41]. In this study, the abundance of AKR1A1 was 0.74-fold at E19 compared with E14 chicken embryos. Aldehyde dehydrogenases (ALDHs) are a class of enzymes that can lead carbohydrates, lipids, and amino acids to form aldehydes during the metabolic process. ALDH9A1 was highly expressed in various tissues [42] and it mainly participates in γ-aminobutyric acid (GABA) metabolism [43], which contributes to supplying carbons to the citric acid cycle in the absence of glucose. ALDH3A2 and ALDH7A1 levels increased at E19 compared with E14 in chicken embryos and were enriched for the glycolysis/gluconeogenesis pathway. These results indicated that increasing levels of ALDH3A2 and ALDH7A1 might lead to decreasing glucose utilization and promotion of gluconeogenesis. Fructose-1, 6-bisphosphatase (FBP), which has two isoenzymes (FBP1 and FBP2), is one of the key enzymes in the glucose metabolism pathway in mammals. Li et al. inferred that FBP1, a rate-limiting enzyme in gluconeogenesis, has a similar function in chickens compared with its homolog in humans [44]. FBP2 is mainly involved in the gluconeogenesis pathway, which can increase glycogen content. Glucose-6-phosphate isomerase (GPI) catalyzes glucose-6-phosphate to fructose-6-phosphate in glycolysis. GPI and pyruvate carboxylase (PC) play important roles in gluconeogenesis in the liver [45]. Our results showed that the levels of FBP1, FBP2, GPI, PC, and lactate dehydrogenase A chain (LDHA) were increased from E14 to E19 in chicken embryos. These results indicated that gluconeogenesis was promoted and was the main method of generating energy at this stage of chicken embryonic development. In addition, phosphoglucomutase (PGM) deficiency was observed under condition of glycolysis in vitro, which leads to the production of lactic acid. Increased levels of PGM-2 (an isoform of PGM) suggested that glycolysis decreased from E14 to E19 during chicken embryonic development. From E14 to E19 in chicken embryos, oxygen consumption was enhanced for energy requirement [46], and the changes in metabolism might be affected by nutrient composition in the eggs [47]. Taken together, our results indicated that gluconeogenesis was enhanced E14 to E19, which was mainly achieved by increasing the levels of FBP, GPI, PC, and LDHA to utilize emergency fuel reserves during embryonic development.

Similarly, the abundance of 160 hepatic proteins was significantly altered from E19 to H1 in chicken embryos, and the differentially abundant proteins were mainly enriched in glycolysis/gluconeogenesis, purine metabolism, and ribosome metabolism according to pathway enrichment analysis. As discussed above, 12 central functional proteins (ACSS1, AKR1A1, ALDH3A2, ALDH7A1, ALDH9A1, FBP1, FBP2, GPI, LDHA, PGM1, and PGM2) were enriched in glycolysis/gluconeogenesis from E14 to E19, while only five central functional proteins (AKR1A1, GAPDH, LDHA, LDHB, and PGM2) were enriched from E19 to H1 in chicken embryos. Among them, only AKR1A1 and LDHA were both enriched in the glycolysis/gluconeogenesis pathway in both comparisons in chicken embryos. However, the levels of AKR1A1 and LDHA decreased from E19 to H1, which was the opposite of that from E14 to E19-. In addition, we found that the levels of LDHB and GAPDA decreased, while that of PGM2 increased from E19 to H1 in chicken embryonic development. A decrease in AKR1A1 and an increase in PGM2 could inhibit the glycolysis/gluconeogenesis pathway [41]. Glycolysis is the metabolic pathway that converts glucose into pyruvate and forms the high-energy molecule ATP, while gluconeogenesis is a metabolic pathway that generates glucose from certain non-carbohydrate carbon substrates. Although there was a discrepancy in the differential abundance of proteins at the two stages of chicken embryonic development, the observed changes in protein abundance indicated that glycolysis is not the main method to produce energy from E19 to H1 in chicken embryos.

The levels of five central functional proteins of purine metabolism (IMPDH2, NT5C, PGM2, PPAT and XDH) changed significantly from E19 to H1 in chicken embryos. Monophosphate dehydrogenase 2 (IMPDH2), a rate-limiting enzyme of de novo guanosine biosynthesis [48], increased from E19 to H1. It was reported that deletion of *Impdh* could lead to early lethality during embryonic development in mice [49]. In addition, we found that the levels of cytosolic purine 5′-nucleotidase (NT5C), amidophosphoribosyltransferase (PPAT), and xanthine dehydrogenase (XDH) increased from E19 to H1 in chicken embryos. A recent study reported that NT5C overexpression decreased dNTP pools and negatively regulated nucleotide synthesis [50]. PPAT is a key enzyme in the first reaction of de novo purine biosynthesis [51] and XDH is involved in the oxidative metabolism of purines [52]. At the end of incubation, the beak of chicken embryo is unchanged and hatching muscle development matures. Meanwhile, chicken embryos need more energy for the transit to dietary feeding as a new energy source. Thus, enhanced purine metabolism case by increases in IMPDH2, NT5C, PGM2 and XDH from E19 to H1 in chicken embryos probably represents preparation for the emergence and growth of chicken embryos.

In animals and humans, ribosomes are essential for growth and comprise a small 40S subunit and a large 60S subunit. In this study, the levels of six central functional proteins (RPL18A, RPL19, RPL7A, RPLP1, RPS2, and RPS3A) increased from E19 to H1 in chicken embryos. Among them, RPL18A, RPL19, RPL7A, and RPLP1 belong to the 60S subunit, and RPS2 and RPS3A belong to the 40S subunit. The function of the 40S subunit is to bring mRNA and aminoacylated tRNAs together, while the 60S subunit mainly catalyzes peptide bond formation [53]. The function loss of ribosomal proteins could affect ribosomal biogenesis and result in human disease [54]. The phenomenon of decreased growth rate caused by bisphenol A could be overcome by upregulating ribosomal biogenesis in *Saccharomyces cerevisiae* [55]. At the end of incubation, embryos are capable of respiratory movement, stomach contraction, beak tucking, and other organism developments that all require the involvement of proteins. In addition, as chicken embryos begin to break out of their shells, and they are faced a change of environment on hatching day. Thus, the increased levels of ribosomal proteins indicated that protein synthesis had increased from E19 to H1 in chicken embryos, which would result in higher levels of functional proteins for the emergence and growth of chicken during this special period of development.

## Conclusion

To the best of our knowledge, this is the first study to identify changes in protein abundance at different stages of chicken embryonic development using iTRAQ-based comparative proteomics analysis. To satisfy requirement for embryonic growth and development, lipolysis (upregulation of ACAA2, CPT1A and ACOX1 abundance), protein folding (upregulation of PDIs, CALR3, LMAN1 and UBQLN1 abundance), and gluconeogenesis (upregulation of FBP, GPI, PC and LDHA abundance) were enhanced from E14 to E19 in chicken embryos. Glycolysis still was not the main method to produce energy, while the purine metabolism (upregulation of IMPDH2, NT5C, PGM2 and XDH abundance) and protein synthesis (upregulation of RPL18A, RPL19, RPL7A, RPLP1, RPS2 and RPS3A abundance) were increased from E19 to H1 in chicken embryos, which prepared the embryos for emergence and growth. Taken together, these results indicated that metabolism changes dramatically to satisfy the requirement for physiological growth and development at different stages in chicken embryos, and that ACAA2, CPT1A, and ACOX1 might be the key factors that control fatty deposition in chicken embryos. These results provide valuable information for chickens as a model for further investigation of the mechanism of obesity and insulin resistance in humans.

## Additional files

Additional file 1: Figure S1. The distribution of peptide length, peptide number, protein mass and protein’s sequences coverage. (DOCX 243 kb)

Additional file 2: Table S1. Differentially expressed proteins at E19d when compared to E14d in chicken embryos. (DOCX 84 kb)

Additional file 3: Table S2. Differentially expressed proteins at H1d when compared to E19d in chicken embryos. (DOCX 68 kb)

Additional file 4: Table S3. The overlapping of differentially expressed abundance proteins on both comparisons in chicken embryos. (DOCX 24 kb)

Additional file 5: Table S4. Differentially expressed proteins with no annotated functions at E19d when compared to E14d in chicken embryos. (DOCX 41 kb)

Additional file 6: Table S5. Differentially expressed proteins with no annotated functions at H1d when compared to E19d in chicken embryos. (DOCX 32 kb)

Additional file 7: Table S6. Detailed pathway enrichment of differential expression protein between E14d and E19d in chicken embryos. (DOCX 22 kb)

Additional file 8: Table S7. Detailed pathway enrichment of differential expression protein between E19d and H1d in chicken embryos. (DOCX 22 kb)

Additional file 9: Table S8. Differentially expressed proteins which were not assigned to any known functions at E19d when compared to E14d in chicken embryos. (DOCX 33 kb)

Additional file 10: Table S9. Differentially expressed proteins which were not assigned to any known functions at H1d when compared to E19d in chicken embryos. (DOCX 28 kb)

## Abbreviations

BP: Biological process
CC: Cellular component
GO: Gene Ontology
iTRAQ: Isobaric tags for relative and absolute quantitation
KEGG: Kyoto Encyclopedia of Genes and Genomes
MF: Molecular function
STRING: The Search Tool for the Retrieval of Interacting Genes/Proteins
