# Supplementary material for: Proteomics reveals changes in hepatic proteins during chicken embryonic development: an alternative model to study human obesity
Source: BMC Genomics. 2018 Jan 8;19:29. doi: 10.1186/s12864-017-4427-6 (PMC5759888; doi:10.1186/s12864-017-4427-6)
Supplement: Supplementary file 1 — The distribution of peptide length, peptide number, protein mass and protein’s sequences coverage. (DOCX 243 kb) [file 12864_2017_4427_MOESM1_ESM.docx]

**Online additional file**

**Proteomics analysis reveals hepatic proteins changes during chicken embryonic development：An alternative model for human obesity study**

Mengling Peng, Shengnan Li, Qianqian He, Jinlong Zhao, Longlong Li, Haitian Ma*

**Additional Figure 1.** The distribution of peptide length, peptide number, protein mass and protein’s sequences coverage.


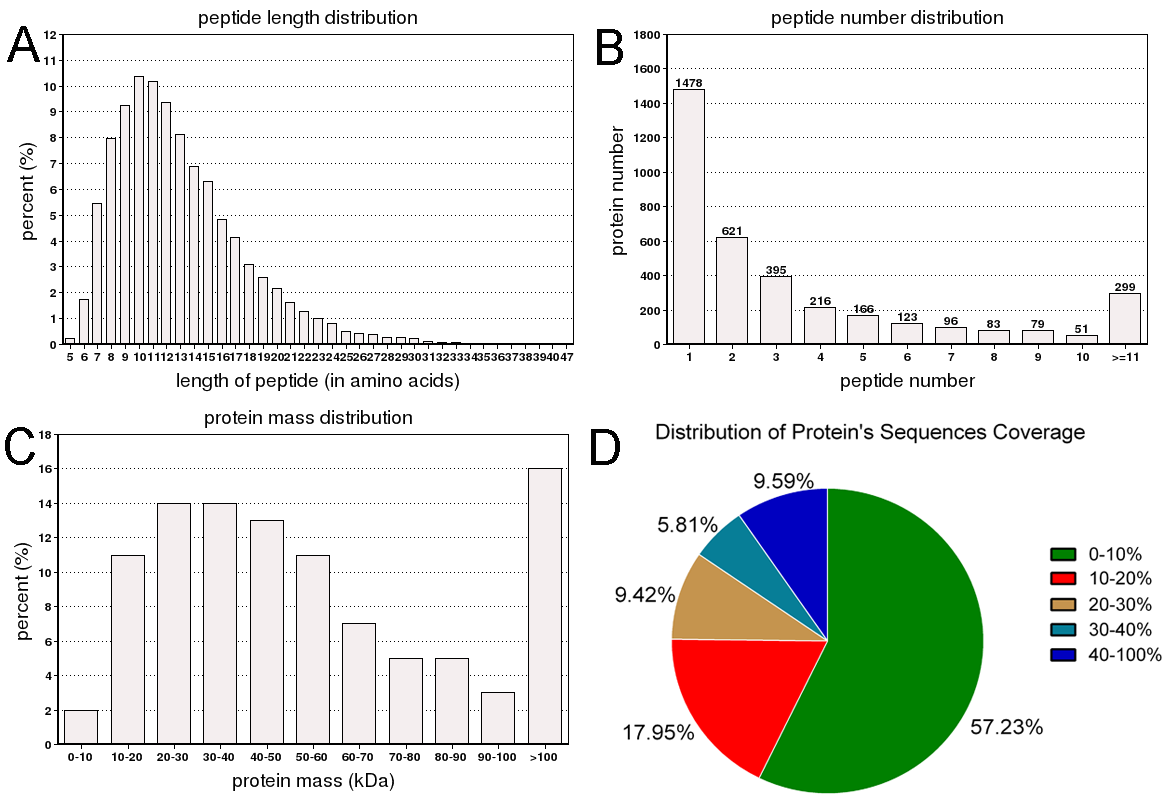


A: peptide length distribution; B: peptide number distribution; C: protein mass distribution; D: protein’s sequences coverage.
