# Supplementary material for: Proteomics reveals changes in hepatic proteins during chicken embryonic development: an alternative model to study human obesity
Source: BMC Genomics. 2018 Jan 8;19:29. doi: 10.1186/s12864-017-4427-6 (PMC5759888; doi:10.1186/s12864-017-4427-6)
Supplement: Supplementary file 2 — Differentially expressed proteins at E19d when compared to E14d in chicken embryos. (DOCX 84 kb) [file 12864_2017_4427_MOESM2_ESM.docx]

**Online Additional file**

**Proteomics analysis reveals hepatic proteins changes during chicken embryonic development：An alternative model for human obesity study**

Mengling Peng, Shengnan Li, Qianqian He, Jinlong Zhao, Longlong Li, Haitian Ma*

**Additional Table 1.** Differentially expressed proteins at E19d when compared to E14d in chicken embryos

| Gene Ontology | NCBInr Description | NCBInr Accession | Species | Uniq_Pep _Num | Uniq_Spec_Num | Protein Coverage | NCBInr Identity | Ratio | P-value | Tendency |
| --- | --- | --- | --- | --- | --- | --- | --- | --- | --- | --- |
| EXOSC9 | exosome complex exonuclease RRP45 | gi\|77797833 | *Gallus gallus* | 1 | 1 | 0.023 | 100 | 0.100 | 0.001 | ↓ |
| SLC2A9 | solute carrier family 2, facilitated glucose transporter member 9 | gi\|363733819 | *Gallus gallus* | 1 | 1 | 0.017 | 100 | 0.168 | 0.004 | ↓ |
| AKAP9 | A-kinase anchor protein 9 | gi\|158186693 | *Gallus gallus* | 1 | 1 | 0.004 | 100 | 0.169 | 0.004 | ↓ |
| COL5A1 | collagen alpha-1(V) chain precursor | gi\|46048885 | *Gallus gallus* | 1 | 2 | 0.023 | 100 | 0.378 | 0.001 | ↓ |
| DUT | deoxyuridine 5'-triphosphate nucleotidohydrolase, mitochondrial | gi\|118405166 | *Gallus gallus* | 1 | 2 | 0.072 | 100 | 0.446 | 0.001 | ↓ |
| AFP | alpha-fetoprotein | gi\|363733434 | *Gallus gallus* | 10 | 36 | 0.192 | 99.78 | 0.459 | 0.001 | ↓ |
| H1 | Histone H1 | gi\|121944 | *Gallus gallus* | 5 | 11 | 0.225 | 100 | 0.481 | 0.001 | ↓ |
| RPL31 | 60S ribosomal protein L31 | gi\|118084259 | *Gallus gallus* | 1 | 2 | 0.086 | 100 | 0.508 | 0.016 | ↓ |
| RBP4 | retinol-binding protein 4 precursor | gi\|45382541 | *Gallus gallus* | 3 | 7 | 0.194 | 100 | 0.58 | 0.023 | ↓ |
| MRPS6 | 28S ribosomal protein S6, mitochondrial | gi\|71895585 | *Gallus gallus* | 2 | 4 | 0.165 | 100 | 0.596 | 0.001 | ↓ |
| RSL1D1 | ribosomal L1 domain containing 1 | gi\|208609946 | *Gallus gallus* | 1 | 3 | 0.018 | 100 | 0.601 | 0.008 | ↓ |
| WDR77 | methylosome protein 50 | gi\|71895697 | *Gallus gallus* | 4 | 6 | 0.17 | 99.9 | 0.607 | 0.001 | ↓ |
| FETUB | fetuin-B | gi\|50752383 | *Gallus gallus* | 6 | 11 | 0.216 | 100 | 0.614 | 0.013 | ↓ |
| DNAJC9 | dnaJ homolog subfamily C member 9 | gi\|313747537 | *Gallus gallus* | 3 | 6 | 0.112 | 100 | 0.616 | 0.005 | ↓ |
| NCL | nucleolin | gi\|45384000 | *Gallus gallus* | 17 | 52 | 0.266 | 98.61 | 0.62 | 0.001 | ↓ |
| GATM | Glycine amidinotransferase, mitochondrial | gi\|308191432 | *Gallus gallus* | 19 | 123 | 0.6 | 100 | 0.621 | 0.001 | ↓ |
| PPIB | peptidyl-prolyl cis-trans isomerase B precursor | gi\|45382027 | *Gallus gallus* | 1 | 5 | 0.844 | 100 | 0.627 | 0.001 | ↓ |
| ALB | serum albumin precursor | gi\|45383974 | *Gallus gallus* | 2 | 40 | 0.636 | 99.36 | 0.634 | 0.001 | ↓ |
| MCM4 | DNA replication licensing factor mcm4 | gi\|118086936 | *Gallus gallus* | 7 | 11 | 0.087 | 100 | 0.642 | 0.001 | ↓ |
| NUMA1 | nuclear mitotic apparatus protein 1 | gi\|300116724 | *Gallus gallus* | 4 | 4 | 0.023 | 100 | 0.646 | 0.018 | ↓ |
| EIF5 | eukaryotic translation initiation factor 5 | gi\|57529904 | *Gallus gallus* | 5 | 12 | 0.123 | 100 | 0.652 | 0.004 | ↓ |
| HP1BP3 | heterochromatin protein 1-binding protein 3 | gi\|57529541 | *Gallus gallus* | 3 | 7 | 0.063 | 100 | 0.653 | 0.009 | ↓ |
| MCM5 | DNA replication licensing factor MCM5 | gi\|57525409 | *Gallus gallus* | 7 | 8 | 0.139 | 100 | 0.657 | 0.001 | ↓ |
| AHSA1 | activator of 90 kDa heat shock protein ATPase homolog 1 isoform 2 | gi\|50748536 | *Gallus gallus* | 3 | 3 | 0.079 | 100 | 0.659 | 0.047 | ↓ |
| RCN2 | reticulocalbin-2 | gi\|118095590 | *Gallus gallus* | 1 | 4 | 0.03 | 100 | 0.66 | 0.001 | ↓ |
| RPL7A | 60S ribosomal protein L7a | gi\|52138653 | *Gallus gallus* | 8 | 19 | 0.389 | 99.66 | 0.66 | 0.001 | ↓ |
| NPM3 | nucleoplasmin-3 | gi\|363735530 | *Gallus gallus* | 2 | 5 | 0.135 | 100 | 0.663 | 0.003 | ↓ |
| TPT1 | translationally-controlled tumor protein homolog | gi\|45382329 | *Gallus gallus* | 2 | 16 | 0.105 | 100 | 0.666 | 0.001 | ↓ |
| FKBP10 | peptidyl-prolyl cis-trans isomerase FKBP10-like isoform 1 | gi\|363743597 | *Gallus gallus* | 5 | 9 | 0.121 | 99.72 | 0.668 | 0.001 | ↓ |
| AK3 | AMP phosphotransferase, mitochondrial-like | gi\|363744423 | *Gallus gallus* | 6 | 15 | 0.33 | 100 | 0.672 | 0.001 | ↓ |
| SLC4A4 | electrogenic sodium bicarbonate cotransporter 1 isoform 2 | gi\|118090277 | *Gallus gallus* | 2 | 3 | 0.023 | 100 | 0.673 | 0.027 | ↓ |
| HMBS | porphobilinogen deaminase | gi\|118101810 | *Gallus gallus* | 5 | 7 | 0.142 | 100 | 0.676 | 0.021 | ↓ |
| SYT1 | synaptotagmin-1 | gi\|45384016 | *Gallus gallus* | 3 | 6 | 0.078 | 100 | 0.677 | 0.001 | ↓ |
| FKBP8 | peptidyl-prolyl cis-trans isomerase FKBP8 | gi\|361549440 | *Gallus gallus* | 5 | 10 | 0.159 | 100 | 0.678 | 0.001 | ↓ |
| RPS7 | 40S ribosomal protein S7 isoform 6 | gi\|118088890 | *Gallus gallus* | 7 | 24 | 0.474 | 100 | 0.678 | 0.001 | ↓ |
| TSR1 | pre-rRNA-processing protein TSR1 homolog | gi\|363741127 | *Gallus gallus* | 2 | 4 | 0.031 | 100 | 0.681 | 0.03 | ↓ |
| HRG | histidine-rich glycoprotein | gi\|410610509 | *Gallus gallus* | 1 | 3 | 0.021 | 99.15 | 0.682 | 0.031 | ↓ |
| CA5B | carbonic anhydrase 5B, mitochondrial | gi\|363738234 | *Gallus gallus* | 3 | 7 | 0.13 | 99.09 | 0.682 | 0.036 | ↓ |
| NPM1 | nucleophosmin | gi\|45383996 | *Gallus gallus* | 7 | 34 | 0.335 | 100 | 0.684 | 0.001 | ↓ |
| TTR | transthyretin precursor | gi\|45384444 | *Gallus gallus* | 6 | 24 | 0.707 | 100 | 0.686 | 0.001 | ↓ |
| SSB | Sjogren syndrome antigen B (autoantigen La) isoform 1 | gi\|302488427 | *Gallus gallus* | 5 | 13 | 0.139 | 100 | 0.689 | 0.001 | ↓ |
| VIM | vimentin | gi\|114326309 | *Gallus gallus* | 14 | 39 | 0.437 | 100 | 0.695 | 0.001 | ↓ |
| CALR3 | calreticulin | gi\|118103332 | *Gallus gallus* | 1 | 16 | 0.017 | 99.26 | 0.695 | 0.001 | ↓ |
| RPL27A | 60S ribosomal protein L27a | gi\|118091246 | *Gallus gallus* | 4 | 9 | 0.27 | 100 | 0.696 | 0.001 | ↓ |
| HBBR | hemoglobin subunit rho | gi\|52138683 | *Gallus gallus* | 1 | 6 | 0.435 | 100 | 0.701 | 0.005 | ↓ |
| SNW1 | SNW domain-containing protein 1 | gi\|50748542 | *Gallus gallus* | 2 | 4 | 0.035 | 99.85 | 0.703 | 0.016 | ↓ |
| RPL24 | 60S ribosomal protein L24 | gi\|118083604 | *Gallus gallus* | 3 | 11 | 0.191 | 100 | 0.71 | 0.001 | ↓ |
| OXCT1 | succinyl-CoA | gi\|60592998 | *Gallus gallus* | 6 | 10 | 0.143 | 100 | 0.71 | 0.007 | ↓ |
| SH3BGRL | SH3 domain-binding glutamic acid-rich-like protein | gi\|60302796 | *Gallus gallus* | 5 | 10 | 0.614 | 100 | 0.71 | 0.002 | ↓ |
| TARDBP | TAR DNA-binding protein 43 | gi\|71894865 | *Gallus gallus* | 3 | 5 | 0.08 | 100 | 0.711 | 0.014 | ↓ |
| MRPS36 | 28S ribosomal protein S36, mitochondrial | gi\|363744215 | *Gallus gallus* | 1 | 7 | 0.126 | 99.69 | 0.713 | 0.001 | ↓ |
| SCUBE2 | signal peptide, CUB domain, EGF-like 2 | gi\|363734260 | *Gallus gallus* | 1 | 7 | 0.008 | 100 | 0.714 | 0.001 | ↓ |
| BDH1 | D-beta-hydroxybutyrate dehydrogenase, mitochondrial precursor | gi\|57529615 | *Gallus gallus* | 1 | 5 | 0.03 | 100 | 0.715 | 0.015 | ↓ |
| SNRPG | uncharacterized protein LOC771344 isoform 1 | gi\|118101457 | *Gallus gallus* | 1 | 7 | 0.158 | 100 | 0.716 | 0.002 | ↓ |
| ADD3 | hypothetical protein RCJMB04_6m11 | gi\|60098711 | *Gallus gallus* | 4 | 7 | 0.078 | 100 | 0.716 | 0.005 | ↓ |
| HNRNPA1 | heterogeneous nuclear ribonucleoprotein A1 | gi\|71895177 | *Gallus gallus* | 9 | 13 | 0.654 | 100 | 0.718 | 0.003 | ↓ |
| RPS26 | 40S ribosomal protein S26-like | gi\|363746266 | *Gallus gallus* | 2 | 7 | 0.209 | 100 | 0.727 | 0.005 | ↓ |
| PPIB | peptidyl-prolyl cis-trans isomerase B precursor | gi\|45382027 | *Gallus gallus* | 2 | 52 | 0.667 | 100 | 0.728 | 0.001 | ↓ |
| ALDOC | fructose-bisphosphate aldolase C | gi\|330417943 | *Gallus gallus* | 8 | 30 | 0.365 | 100 | 0.729 | 0.001 | ↓ |
| PBDC1 | hypothetical protein RCJMB04_33c16 | gi\|53136736 | *Gallus gallus* | 3 | 4 | 0.182 | 100 | 0.732 | 0.022 | ↓ |
| EEF1B2 | hypothetical protein RCJMB04_32c11 | gi\|53136666 | *Gallus gallus* | 7 | 30 | 0.71 | 100 | 0.732 | 0.001 | ↓ |
| FSCN1 | fascin | gi\|296011017 | *Gallus gallus* | 6 | 10 | 0.163 | 100 | 0.734 | 0.013 | ↓ |
| RBBP4 | histone-binding protein RBBP4 | gi\|45382339 | *Gallus gallus* | 3 | 9 | 0.179 | 100 | 0.734 | 0.001 | ↓ |
| FUS | RNA-binding protein FUS | gi\|48374057 | *Gallus gallus* | 4 | 12 | 0.133 | 100 | 0.735 | 0.001 | ↓ |
| AKR1A1 | alcohol dehydrogenase | gi\|57529654 | *Gallus gallus* | 8 | 24 | 0.275 | 100 | 0.737 | 0.001 | ↓ |
| AGXT2L1 | alanine-glyoxylate aminotransferase 2-like 1 | gi\|363733160 | *Gallus gallus* | 10 | 35 | 0.252 | 100 | 0.737 | 0.001 | ↓ |
| STRAP | serine-threonine kinase receptor-associated protein | gi\|347800736 | *Gallus gallus* | 5 | 13 | 0.166 | 100 | 0.739 | 0.001 | ↓ |
| ENOPH1 | enolase-phosphatase E1 | gi\|363733238 | *Gallus gallus* | 3 | 7 | 0.115 | 100 | 0.739 | 0.012 | ↓ |
| IGF2BP1 | insulin-like growth factor 2 mRNA-binding protein 1 | gi\|45384194 | *Gallus gallus* | 6 | 10 | 0.175 | 100 | 0.741 | 0.011 | ↓ |
| RBMX | heterogeneous nuclear ribonucleoprotein G | gi\|119331082 | *Gallus gallus* | 4 | 10 | 0.112 | 100 | 0.744 | 0.001 | ↓ |
| PPIE | peptidyl-prolyl cis-trans isomerase E | gi\|118101790 | *Gallus gallus* | 3 | 6 | 0.136 | 100 | 0.745 | 0.021 | ↓ |
| TCERG1 | uncharacterized protein LOC416349 | gi\|363739271 | *Gallus gallus* | 7 | 9 | 0.064 | 100 | 0.745 | 0.014 | ↓ |
| PNPO | pyridoxine-5'-phosphate oxidase-like, partial | gi\|363746851 | *Gallus gallus* | 4 | 9 | 0.359 | 100 | 0.745 | 0.001 | ↓ |
| RPLP1 | 60S acidic ribosomal protein P1 | gi\|45384350 | *Gallus gallus* | 1 | 10 | 0.14 | 100 | 0.747 | 0.012 | ↓ |
| PAPSS1 | bifunctional 3'-phosphoadenosine 5'-phosphosulfate synthase 1 | gi\|363733166 | *Gallus gallus* | 5 | 7 | 0.112 | 100 | 0.748 | 0.015 | ↓ |
| PCNA | proliferating cell nuclear antigen | gi\|45383776 | *Gallus gallus* | 7 | 14 | 0.366 | 100 | 0.749 | 0.001 | ↓ |
| CCT6A | T-complex protein 1 subunit zeta | gi\|57525300 | *Gallus gallus* | 16 | 76 | 0.385 | 100 | 0.749 | 0.001 | ↓ |
| PARP1 | Poly | gi\|3220000 | *Gallus gallus* | 10 | 15 | 0.137 | 100 | 0.752 | 0.001 | ↓ |
| ADI1 | 1,2-dihydroxy-3-keto-5-methylthiopentene dioxygenase | gi\|71895073 | *Gallus gallus* | 6 | 12 | 0.461 | 100 | 0.753 | 0.001 | ↓ |
| RPA1 | replication protein A 70 kDa DNA-binding subunit | gi\|57525314 | *Gallus gallus* | 4 | 5 | 0.112 | 100 | 0.753 | 0.036 | ↓ |
| SUGT1 | suppressor of G2 allele of SKP1 homolog | gi\|302486333 | *Gallus gallus* | 4 | 7 | 0.168 | 99.98 | 0.754 | 0.005 | ↓ |
| FKBP1A | peptidyl-prolyl cis-trans isomerase FKBP1A | gi\|45383498 | *Gallus gallus* | 4 | 22 | 0.556 | 100 | 0.755 | 0.001 | ↓ |
| CSDE1 | cold shock domain-containing protein E1 | gi\|71896273 | *Gallus gallus* | 9 | 19 | 0.135 | 100 | 0.756 | 0.001 | ↓ |
| ERP29 | endoplasmic reticulum resident protein 29 precursor | gi\|444741647 | *Gallus gallus* | 6 | 32 | 0.261 | 100 | 0.757 | 0.001 | ↓ |
| PFDN2 | prefoldin subunit 2-like | gi\|363742957 | *Gallus gallus* | 5 | 12 | 0.321 | 100 | 0.758 | 0.002 | ↓ |
| IMPDH2 | inosine-5'-monophosphate dehydrogenase 2 | gi\|71895387 | *Gallus gallus* | 7 | 11 | 0.21 | 100 | 0.761 | 0.006 | ↓ |
| PSMF1 | proteasome inhibitor PI31 subunit | gi\|71896121 | *Gallus gallus* | 4 | 7 | 0.173 | 100 | 0.764 | 0.005 | ↓ |
| CKAP4 | cytoskeleton-associated protein 4 | gi\|363727954 | *Gallus gallus* | 8 | 14 | 0.21 | 100 | 0.765 | 0.001 | ↓ |
| PSMD9 | 26S proteasome non-ATPase regulatory subunit 9 | gi\|57525182 | *Gallus gallus* | 3 | 4 | 0.164 | 100 | 0.766 | 0.043 | ↓ |
| P4HB | cognin/prolyl-4-hydroxylase/protein disulfide isomerase | gi\|21703694 | *Gallus gallus* | 23 | 273 | 0.579 | 100 | 0.769 | 0.001 | ↓ |
| CCT2 | T-complex protein 1 subunit beta | gi\|60302718 | *Gallus gallus* | 20 | 87 | 0.505 | 100 | 0.774 | 0.001 | ↓ |
| UCHL5 | ubiquitin carboxyl-terminal hydrolase isozyme L5 | gi\|57529689 | *Gallus gallus* | 6 | 10 | 0.232 | 100 | 0.774 | 0.004 | ↓ |
| LMNB2 | lamin-B2 | gi\|45384202 | *Gallus gallus* | 12 | 25 | 0.243 | 100 | 0.774 | 0.001 | ↓ |
| FAM222B | uncharacterized protein LOC417578 | gi\|71897055 | *Gallus gallus* | 1 | 7 | 0.029 | 100 | 0.775 | 0.023 | ↓ |
| HCCS | cytochrome c-type heme lyase | gi\|71895281 | *Gallus gallus* | 2 | 5 | 0.084 | 100 | 0.776 | 0.019 | ↓ |
| SYNCRIP | synaptotagmin binding, cytoplasmic RNA interacting protein | gi\|71894995 | *Gallus gallus* | 8 | 19 | 0.326 | 100 | 0.777 | 0.001 | ↓ |
| PABPC1 | polyadenylate-binding protein 1 | gi\|71896197 | *Gallus gallus* | 12 | 42 | 0.316 | 100 | 0.777 | 0.001 | ↓ |
| HMGB1 | high mobility group protein HMG1 | gi\|5815432 | *Gallus gallus* | 3 | 12 | 0.191 | 100 | 0.778 | 0.001 | ↓ |
| NT5C3L | cytosolic 5'-nucleotidase III-like protein | gi\|57530543 | *Gallus gallus* | 4 | 7 | 0.149 | 100 | 0.779 | 0.008 | ↓ |
| SEPT 9 | septin-9 | gi\|71897123 | *Gallus gallus* | 3 | 13 | 0.053 | 99.75 | 0.78 | 0.001 | ↓ |
| DPYSL2 | dihydropyrimidinase-related protein 2 | gi\|45383177 | *Gallus gallus* | 1 | 3 | 0.451 | 100 | 0.78 | 0.001 | ↓ |
| LGALS2 | galectin-2-like isoform 1 | gi\|363727877 | *Gallus gallus* | 3 | 7 | 0.248 | 100 | 0.782 | 0.008 | ↓ |
| DDX5 | probable ATP-dependent RNA helicase DDX5 | gi\|45382259 | *Gallus gallus* | 8 | 25 | 0.269 | 100 | 0.783 | 0.001 | ↓ |
| TPM1 | alpha-tropomyosin | gi\|211110 | *Gallus gallus* | 4 | 24 | 0.339 | 100 | 0.783 | 0.001 | ↓ |
| ALDH9A1 | 4-trimethylaminobutyraldehyde dehydrogenase | gi\|118094103 | *Gallus gallus* | 14 | 104 | 0.373 | 100 | 0.783 | 0.001 | ↓ |
| SNRPB | small nuclear ribonucleoprotein-associated protein B' | gi\|45382973 | *Gallus gallus* | 4 | 18 | 0.183 | 100 | 0.784 | 0.001 | ↓ |
| CHP1 | calcineurin B homologous protein 1 | gi\|56118996 | *Gallus gallus* | 4 | 8 | 0.272 | 100 | 0.785 | 0.008 | ↓ |
| CG-16 | 16 kDa beta-galactoside-binding lectin | gi\|46048435 | *Gallus gallus* | 3 | 10 | 0.269 | 99.25 | 0.786 | 0.001 | ↓ |
| TXNDC5 | thioredoxin domain-containing protein 5 precursor | gi\|57530789 | *Gallus gallus* | 11 | 28 | 0.336 | 100 | 0.786 | 0.001 | ↓ |
| FKBP4 | peptidyl-prolyl cis-trans isomerase FKBP4 | gi\|57525441 | *Gallus gallus* | 6 | 24 | 0.204 | 99.74 | 0.786 | 0.001 | ↓ |
| MAPRE1 | microtubule-associated protein RP/EB family member 1 | gi\|71894959 | *Gallus gallus* | 5 | 7 | 0.326 | 100 | 0.787 | 0.028 | ↓ |
| ATP5H | ATP synthase subunit d, mitochondrial isoform 1 | gi\|118099965 | *Gallus gallus* | 10 | 86 | 0.759 | 100 | 0.789 | 0.001 | ↓ |
| SFPQ | splicing factor, proline- and glutamine-rich | gi\|363742292 | *Gallus gallus* | 15 | 29 | 0.332 | 100 | 0.789 | 0.001 | ↓ |
| HNRNPAB | heterogeneous nuclear ribonucleoprotein A/B | gi\|45384514 | *Gallus gallus* | 11 | 22 | 0.616 | 100 | 0.790 | 0.001 | ↓ |
| HYOU1 | hypoxia up-regulated protein 1 precursor | gi\|57528712 | *Gallus gallus* | 19 | 62 | 0.306 | 98.74 | 0.790 | 0.001 | ↓ |
| NFU1 | hypothetical protein RCJMB04_35n21 | gi\|53136898 | *Gallus gallus* | 3 | 5 | 0.155 | 100 | 0.790 | 0.026 | ↓ |
| GC | vitamin D-binding protein precursor | gi\|45382425 | *Gallus gallus* | 11 | 45 | 0.277 | 100 | 0.790 | 0.001 | ↓ |
| DDX17 | probable ATP-dependent RNA helicase DDX17 | gi\|118082784 | *Gallus gallus* | 10 | 24 | 0.288 | 100 | 0.792 | 0.001 | ↓ |
| PPID | peptidyl-prolyl cis-trans isomerase D | gi\|363733121 | *Gallus gallus* | 5 | 13 | 0.132 | 100 | 0.792 | 0.001 | ↓ |
| GRB2 | growth factor receptor-bound protein 2 | gi\|124110120 | *Gallus gallus* | 4 | 8 | 0.157 | 100 | 0.793 | 0.013 | ↓ |
| NME2 | nucleoside diphosphate kinase | gi\|45384260 | *Gallus gallus* | 10 | 86 | 0.817 | 99.52 | 0.793 | 0.001 | ↓ |
| USP10 | ubiquitin carboxyl-terminal hydrolase 10 | gi\|57524928 | *Gallus gallus* | 6 | 7 | 0.095 | 100 | 0.795 | 0.04 | ↓ |
| SULT1C1 | sulfotransferase 1C1 | gi\|45382969 | *Gallus gallus* | 16 | 89 | 0.717 | 99.04 | 0.796 | 0.001 | ↓ |
| HSPA9 | stress-70 protein, mitochondrial precursor | gi\|57524986 | *Gallus gallus* | 23 | 149 | 0.542 | 99.65 | 0.797 | 0.001 | ↓ |
| EIF4H | eukaryotic translation initiation factor 4H | gi\|124249410 | *Gallus gallus* | 4 | 11 | 0.425 | 100 | 0.800 | 0.014 | ↓ |
| ACAD11 | acyl-CoA dehydrogenase family member 11 | gi\|57530751 | *Gallus gallus* | 1 | 1 | 0.153 | 98.9 | 2.703 | 0.042 | ↑ |
| GAL2 | Gal 2 | gi\|113911475 | *Gallus gallus* | 1 | 3 | 0.229 | 100 | 2.263 | 0.011 | ↑ |
| DM5L | dimethylaniline monooxygenase | gi\|363736481 | *Gallus gallus* | 4 | 4 | 0.079 | 100 | 2.226 | 0.018 | ↑ |
| HNMT | histamine N-methyltransferase | gi\|118093867 | *Gallus gallus* | 2 | 4 | 0.092 | 100 | 2.192 | 0.004 | ↑ |
| FBXL12 | hepatic lectin | gi\|45382743 | *Gallus gallus* | 2 | 9 | 0.068 | 100 | 2.011 | 0.001 | ↑ |
| HMGCS1 | hydroxymethylglutaryl-CoA synthase, cytoplasmic | gi\|45382279 | *Gallus gallus* | 7 | 15 | 0.18 | 99.76 | 2.011 | 0.001 | ↑ |
| Comtd1 | catechol O-methyltransferase domain-containing protein 1 | gi\|363735497 | *Gallus gallus* | 5 | 8 | 0.199 | 100 | 1.92 | 0.007 | ↑ |
| DHRS7 | dehydrogenase/reductase SDR family member 7 | gi\|118092288 | *Gallus gallus* | 9 | 74 | 0.393 | 100 | 1.879 | 0.001 | ↑ |
| GGACT | gamma-glutamylaminecyclotransferase-like isoform 2 | gi\|50730609 | *Gallus gallus* | 5 | 25 | 0.307 | 100 | 1.864 | 0.001 | ↑ |
| RHOT2 | mitochondrial Rho GTPase 2 | gi\|124249422 | *Gallus gallus* | 2 | 5 | 0.05 | 100 | 1.861 | 0.001 | ↑ |
| LECT2 | Myeloid protein 1 | gi\|127095 | *Gallus gallus* | 9 | 25 | 0.401 | 100 | 1.783 | 0.001 | ↑ |
| Ephx1 | epoxide hydrolase 1-like | gi\|50740570 | *Gallus gallus* | 15 | 63 | 0.37 | 100 | 1.772 | 0.001 | ↑ |
| PPP2R5A | serine/threonine-protein phosphatase 2A 56 kDa regulatory subunit alpha isoform | gi\|118087908 | *Gallus gallus* | 1 | 2 | 0.016 | 100 | 1.761 | 0.044 | ↑ |
| LOC768709 | uncharacterized protein LOC768709 | gi\|118104643 | *Gallus gallus* | 2 | 3 | 0.13 | 100 | 1.731 | 0.003 | ↑ |
| FAAH | fatty-acid amide hydrolase 1 | gi\|50751550 | *Gallus gallus* | 4 | 7 | 0.09 | 100 | 1.725 | 0.001 | ↑ |
| A2M | alpha-2-macroglobulin | gi\|363728304 | *Gallus gallus* | 9 | 18 | 0.165 | 98.82 | 1.723 | 0.001 | ↑ |
| SURF4 | surfeit locus protein 4 | gi\|45383444 | *Gallus gallus* | 2 | 19 | 0.104 | 100 | 1.722 | 0.001 | ↑ |
| MGIST3 | microsomal glutathione S-transferase 3 isoform 2 | gi\|50751077 | *Gallus gallus* | 1 | 4 | 0.045 | 100 | 1.72 | 0.029 | ↑ |
| SLC25A3 | phosphate carrier protein, mitochondrial | gi\|57525378 | *Gallus gallus* | 5 | 24 | 0.161 | 100 | 1.713 | 0.001 | ↑ |
| RPN2 | dolichyl-diphosphooligosaccharide--protein glycosyltransferase subunit 2 precursor | gi\|57529367 | *Gallus gallus* | 5 | 20 | 0.136 | 100 | 1.713 | 0.001 | ↑ |
| SLC25A1 | tricarboxylate transport protein, mitochondrial | gi\|118098357 | *Gallus gallus* | 8 | 78 | 0.3 | 98.4 | 1.71 | 0.001 | ↑ |
| PIT 54 | PIT 54 protein precursor | gi\|46395491 | *Gallus gallus* | 10 | 30 | 0.328 | 100 | 1.709 | 0.001 | ↑ |
| RSPRY1 | RING finger and SPRY domain-containing protein 1 | gi\|50753502 | *Gallus gallus* | 1 | 3 | 0.014 | 98.34 | 1.685 | 0.006 | ↑ |
| HSD17B11 | estradiol 17-beta-dehydrogenase 11 isoform 2 | gi\|50746549 | *Gallus gallus* | 8 | 45 | 0.284 | 100 | 1.678 | 0.001 | ↑ |
| ANXA5 | annexin A5 | gi\|71895873 | *Gallus gallus* | 17 | 158 | 0.713 | 100 | 1.669 | 0.001 | ↑ |
| SLC25A20 | mitochondrial carnitine/acylcarnitine carrier protein | gi\|50754473 | *Gallus gallus* | 7 | 30 | 0.296 | 100 | 1.668 | 0.001 | ↑ |
| SDR16C5 | epidermal retinol dehydrogenase 2 | gi\|71895993 | *Gallus gallus* | 5 | 13 | 0.243 | 99.49 | 1.66 | 0.001 | ↑ |
| ATP8 | ATP synthase F0 subunit 8 | gi\|198401599 | *Gallus gallus* | 2 | 11 | 0.643 | 97.99 | 1.658 | 0.001 | ↑ |
| AGPAT3 | 1-acyl-sn-glycerol-3-phosphate acyltransferase gamma | gi\|118083886 | *Gallus gallus* | 6 | 16 | 0.165 | 99.77 | 1.654 | 0.005 | ↑ |
| SPINK7 | ovomucoid | gi\|209979542 | *Gallus gallus* | 2 | 5 | 0.157 | 98.52 | 1.652 | 0.016 | ↑ |
| SLC27A2 | very long-chain acyl-CoA synthetase, partial | gi\|363747167 | *Gallus gallus* | 7 | 21 | 0.259 | 100 | 1.637 | 0.001 | ↑ |
| CYP4V2 | cytochrome P450, family 4, subfamily V, polypeptide 2 | gi\|50657412 | *Gallus gallus* | 6 | 11 | 0.143 | 100 | 1.635 | 0.005 | ↑ |
| SCARB2 | lysosome membrane protein 2 | gi\|50746651 | *Gallus gallus* | 9 | 31 | 0.249 | 100 | 1.622 | 0.001 | ↑ |
| MAT1A | S-adenosylmethionine synthase isoform type-1 | gi\|313760551 | *Gallus gallus* | 16 | 57 | 0.518 | 100 | 1.603 | 0.001 | ↑ |
| CYP2U1 | cytochrome P450 2U1-like | gi\|363733294 | *Gallus gallus* | 8 | 23 | 0.169 | 100 | 1.592 | 0.001 | ↑ |
| LOC396380 | glutathione S-transferase 3 | gi\|47604962 | *Gallus gallus* | 7 | 28 | 0.402 | 100 | 1.585 | 0.001 | ↑ |
| GST3 | fatty acyl-CoA hydrolase precursor, medium chain isoform 2 | gi\|363738173 | *Gallus gallus* | 7 | 35 | 0.395 | 100 | 1.585 | 0.001 | ↑ |
| NDUFA9 | NADH dehydrogenase | gi\|57529307 | *Gallus gallus* | 9 | 21 | 0.328 | 100 | 1.578 | 0.001 | ↑ |
| TMEM30A | cell cycle control protein 50A | gi\|61097955 | *Gallus gallus* | 1 | 4 | 0.022 | 100 | 1.551 | 0.001 | ↑ |
| SRPRB | hypothetical protein RCJMB04_19k4 | gi\|53133718 | *Gallus gallus* | 4 | 6 | 0.182 | 99.03 | 1.545 | 0.017 | ↑ |
| SCP2 | Non-specific lipid-transfer protein | gi\|2501195 | *Gallus gallus* | 1 | 4 | 0.638 | 100 | 1.539 | 0.019 | ↑ |
| AADACL2 | arylacetamide deacetylase | gi\|118095370 | *Gallus gallus* | 17 | 87 | 0.613 | 100 | 1.539 | 0.001 | ↑ |
| LDHA | L-lactate dehydrogenase A chain | gi\|45384208 | *Gallus gallus* | 6 | 27 | 0.262 | 100 | 1.525 | 0.001 | ↑ |
| AMACR | alpha-methylacyl-CoA racemase | gi\|71895383 | *Gallus gallus* | 8 | 21 | 0.303 | 99.17 | 1.524 | 0.001 | ↑ |
| GPI | glucose-6-phosphate isomerase | gi\|57524920 | *Gallus gallus* | 17 | 46 | 0.459 | 100 | 1.524 | 0.001 | ↑ |
| CPT1A | carnitine O-palmitoyltransferase 1, liver isoform | gi\|61097993 | *Gallus gallus* | 15 | 46 | 0.234 | 100 | 1.517 | 0.001 | ↑ |
| MARC1 | mitochondrial amidoxime-reducing component 1 | gi\|118087865 | *Gallus gallus* | 10 | 61 | 0.336 | 100 | 1.516 | 0.001 | ↑ |
| HBBA | hemoglobin subunit beta | gi\|49169791 | *Gallus gallus* | 7 | 86 | 0.999 | 100 | 1.516 | 0.001 | ↑ |
| CA2 | carbonic anhydrase 2 | gi\|46048696 | *Gallus gallus* | 8 | 26 | 0.469 | 100 | 1.513 | 0.001 | ↑ |
| PTCD1 | pentatricopeptide repeat-containing protein 1 | gi\|363739557 | *Gallus gallus* | 1 | 9 | 0.016 | 100 | 1.508 | 0.001 | ↑ |
| EHHADH | peroxisomal bifunctional enzyme | gi\|118094872 | *Gallus gallus* | 27 | 150 | 0.496 | 100 | 1.506 | 0.001 | ↑ |
| PFKFB4 | 6-phosphofructo-2-kinase/fructose-2,6-bisphosphatase | gi\|71895485 | *Gallus gallus* | 8 | 16 | 0.2 | 100 | 1.506 | 0.002 | ↑ |
| LOC421740 | amine sulfotransferase | gi\|50744552 | *Gallus gallus* | 8 | 24 | 0.315 | 97.07 | 1.505 | 0.001 | ↑ |
| LOC100857820 | uncharacterized oxidoreductase C663.09c-like | gi\|363738114 | *Gallus gallus* | 2 | 7 | 0.581 | 100 | 1.5 | 0.001 | ↑ |
| ARL1 | ADP-ribosylation factor-like protein 1 | gi\|125628642 | *Gallus gallus* | 1 | 7 | 0.05 | 99.82 | 1.487 | 0.001 | ↑ |
| PON2 | serum paraoxonase/arylesterase 2 precursor | gi\|319918842 | *Gallus gallus* | 10 | 48 | 0.345 | 100 | 1.481 | 0.001 | ↑ |
| HSD11B1b | corticosteroid 11-beta-dehydrogenase isozyme 1 | gi\|118102457 | *Gallus gallus* | 11 | 63 | 0.508 | 100 | 1.475 | 0.001 | ↑ |
| FADS6 | fatty acid desaturase 6 | gi\|118099955 | *Gallus gallus* | 3 | 10 | 0.072 | 100 | 1.473 | 0.011 | ↑ |
| Acss1 | acyl-CoA synthetase short-chain family member 1-like | gi\|363734138 | *Gallus gallus* | 12 | 53 | 0.299 | 100 | 1.471 | 0.001 | ↑ |
| RSFR | ribonuclease homolog precursor | gi\|56118294 | *Gallus gallus* | 3 | 5 | 0.302 | 100 | 1.47 | 0.005 | ↑ |
| DECR1 | 2,4-dienoyl-CoA reductase, mitochondrial | gi\|50731694 | *Gallus gallus* | 14 | 86 | 0.503 | 100 | 1.465 | 0.001 | ↑ |
| RNPEP | aminopeptidase B | gi\|363743138 | *Gallus gallus* | 6 | 15 | 0.108 | 100 | 1.465 | 0.001 | ↑ |
| FBP2 | fructose-1,6-bisphosphatase isozyme 2 | gi\|50762391 | *Gallus gallus* | 1 | 3 | 0.06 | 100 | 1.462 | 0.032 | ↑ |
| SLC25A6 | ADP/ATP translocase 3 | gi\|54020693 | *Gallus gallus* | 5 | 20 | 0.5 | 99.27 | 1.44 | 0.001 | ↑ |
| KMO | kynurenine 3-monooxygenase isoform 1 | gi\|363735774 | *Gallus gallus* | 8 | 22 | 0.208 | 100 | 1.438 | 0.001 | ↑ |
| SERPINB14 | Ovalbumin | gi\|129293 | *Gallus gallus* | 11 | 157 | 0.443 | 100 | 1.432 | 0.001 | ↑ |
| AKR1D1 | PREDICTED | gi\|363728195 | *Gallus gallus* | 11 | 37 | 0.505 | 99.78 | 1.431 | 0.001 | ↑ |
| PDCD4 | programmed cell death protein 4 | gi\|45383532 | *Gallus gallus* | 4 | 5 | 0.099 | 99.67 | 1.429 | 0.009 | ↑ |
| MAOA | monoamine oxidase A | gi\|71895251 | *Gallus gallus* | 2 | 4 | 0.069 | 100 | 1.428 | 0.042 | ↑ |
| GSTM2 | glutathione S-transferase 2 | gi\|46048786 | *Gallus gallus* | 14 | 121 | 0.836 | 100 | 1.427 | 0.001 | ↑ |
| SQRDL | sulfide quinone oxidoreductase, mitochondrial | gi\|50752997 | *Gallus gallus* | 5 | 8 | 0.142 | 100 | 1.426 | 0.004 | ↑ |
| ACAD9 | acyl-CoA dehydrogenase family member 9, mitochondrial | gi\|57524955 | *Gallus gallus* | 25 | 132 | 0.529 | 100 | 1.421 | 0.001 | ↑ |
| PAH | phenylalanine-4-hydroxylase | gi\|47604920 | *Gallus gallus* | 15 | 58 | 0.41 | 100 | 1.414 | 0.001 | ↑ |
| DNAJC13 | dnaJ homolog subfamily C member 13 | gi\|118085986 | *Gallus gallus* | 4 | 6 | 0.02 | 100 | 1.414 | 0.034 | ↑ |
| NAA15 | N-alpha-acetyltransferase 15, NatA auxiliary subunit | gi\|363733098 | *Gallus gallus* | 2 | 6 | 0.037 | 99.79 | 1.413 | 0.022 | ↑ |
| ETFDH | electron transfer flavoprotein-ubiquinone oxidoreductase, mitochondrial | gi\|71895853 | *Gallus gallus* | 19 | 76 | 0.547 | 100 | 1.412 | 0.001 | ↑ |
| NT5C2 | cytosolic purine 5'-nucleotidase | gi\|71895075 | *Gallus gallus* | 9 | 23 | 0.225 | 100 | 1.403 | 0.001 | ↑ |
| HBA1 | hemoglobin subunit alpha-A | gi\|52138655 | *Gallus gallus* | 8 | 313 | 0.838 | 100 | 1.4 | 0.001 | ↑ |
| ALDH8A1 | aldehyde dehydrogenase family 8 member A1 isoform 2 | gi\|50742728 | *Gallus gallus* | 8 | 22 | 0.234 | 98.64 | 1.398 | 0.001 | ↑ |
| GPD1L | glycerol-3-phosphate dehydrogenase 1-like | gi\|50732786 | *Gallus gallus* | 2 | 16 | 0.076 | 100 | 1.395 | 0.001 | ↑ |
| DDOST | Dolichyl-diphosphooligosaccharide--protein glycosyltransferase 48 kDa subunit | gi\|1352649 | *Gallus gallus* | 9 | 31 | 0.266 | 100 | 1.394 | 0.001 | ↑ |
| PPM1K | protein phosphatase 1K, mitochondrial | gi\|118090208 | *Gallus gallus* | 3 | 5 | 0.099 | 97.62 | 1.394 | 0.016 | ↑ |
| Fgg | fibrinogen, gamma chain precursor | gi\|45384500 | *Gallus gallus* | 13 | 34 | 0.372 | 100 | 1.392 | 0.001 | ↑ |
| MTTP | microsomal triglyceride transfer protein large subunit precursor | gi\|157954041 | *Gallus gallus* | 28 | 122 | 0.456 | 100 | 1.392 | 0.001 | ↑ |
| NNT | NAD(P) transhydrogenase, mitochondrial | gi\|363744134 | *Gallus gallus* | 24 | 145 | 0.348 | 100 | 1.39 | 0.001 | ↑ |
| HPGDS | hematopoietic prostaglandin D synthase | gi\|45384344 | *Gallus gallus* | 11 | 70 | 0.581 | 100 | 1.39 | 0.001 | ↑ |
| MOGAT1 | 2-acylglycerol O-acyltransferase 1 | gi\|363737112 | *Gallus gallus* | 5 | 15 | 0.221 | 100 | 1.388 | 0.006 | ↑ |
| MYH9 | myosin-9 | gi\|45382693 | *Gallus gallus* | 27 | 66 | 0.222 | 100 | 1.385 | 0.001 | ↑ |
| ACSL1 | long-chain-fatty-acid--CoA ligase 1 | gi\|60302804 | *Gallus gallus* | 24 | 107 | 0.461 | 100 | 1.38 | 0.001 | ↑ |
| FMO6 | flavin containing monooxygenase 6 pseudogene | gi\|45383027 | *Gallus gallus* | 13 | 41 | 0.313 | 99.49 | 1.379 | 0.001 | ↑ |
| SLC25A13 | calcium-binding mitochondrial carrier protein Aralar2 | gi\|61098440 | *Gallus gallus* | 13 | 38 | 0.288 | 98.83 | 1.378 | 0.001 | ↑ |
| BDH2 | 3-hydroxybutyrate dehydrogenase type 2 | gi\|50746905 | *Gallus gallus* | 7 | 29 | 0.376 | 100 | 1.372 | 0.001 | ↑ |
| FTCD | formimidoyltransferase-cyclodeaminase | gi\|45382475 | *Gallus gallus* | 18 | 54 | 0.47 | 100 | 1.372 | 0.001 | ↑ |
| OVALY | ovalbumin-related protein Y | gi\|71897377 | *Gallus gallus* | 7 | 13 | 0.229 | 100 | 1.367 | 0.008 | ↑ |
| SPINK5 | ovoinhibitor precursor | gi\|71895337 | *Gallus gallus* | 4 | 6 | 0.106 | 100 | 1.367 | 0.034 | ↑ |
| CYB5R2 | NADH-cytochrome b5 reductase 2 | gi\|82080903 | *Gallus gallus* | 5 | 16 | 0.343 | 99.77 | 1.367 | 0.008 | ↑ |
| AUH | methylglutaconyl-CoA hydratase, mitochondrial isoform 1 | gi\|357593606 | *Gallus gallus* | 10 | 41 | 0.371 | 100 | 1.366 | 0.001 | ↑ |
| ABCA8 | ATP-binding cassette sub-family A member 10 | gi\|363740873 | *Gallus gallus* | 8 | 14 | 0.056 | 100 | 1.363 | 0.007 | ↑ |
| NIT2 | omega-amidase NIT2 isoform 2 | gi\|363728462 | *Gallus gallus* | 12 | 26 | 0.611 | 100 | 1.362 | 0.001 | ↑ |
| TMED5 | transmembrane emp24 domain-containing protein 5 precursor | gi\|56118980 | *Gallus gallus* | 3 | 5 | 0.142 | 100 | 1.348 | 0.026 | ↑ |
| Mtch2 | mitochondrial carrier homolog 2 | gi\|45382213 | *Gallus gallus* | 5 | 36 | 0.181 | 100 | 1.346 | 0.001 | ↑ |
| ECI2 | enoyl-CoA delta isomerase 2, mitochondrial isoform 2 | gi\|50734079 | *Gallus gallus* | 8 | 29 | 0.291 | 99.4 | 1.345 | 0.001 | ↑ |
| MOV10 | putative helicase MOV-10 | gi\|61098155 | *Gallus gallus* | 6 | 8 | 0.073 | 100 | 1.344 | 0.04 | ↑ |
| FTH | ferritin heavy chain | gi\|45384172 | *Gallus gallus* | 5 | 9 | 0.261 | 100 | 1.342 | 0.038 | ↑ |
| FBP1 | fructose-1,6-bisphosphatase 1 | gi\|50762393 | *Gallus gallus* | 21 | 414 | 0.902 | 95.49 | 1.337 | 0.001 | ↑ |
| FN3K | fructosamine-3-kinase | gi\|50757671 | *Gallus gallus* | 7 | 13 | 0.339 | 100 | 1.336 | 0.04 | ↑ |
| UBQLN1 | ubiquilin-1 isoform 2 | gi\|118104137 | *Gallus gallus* | 2 | 8 | 0.082 | 100 | 1.335 | 0.048 | ↑ |
| LMAN1 | protein ERGIC-53 precursor | gi\|71897199\| | *Gallus gallus* | 10 | 31 | 0.302 | 99.79 | 1.334 | 0.001 | ↑ |
| ATP5F1 | ATP synthase subunit b, mitochondrial | gi\|363743079 | *Gallus gallus* | 11 | 52 | 0.506 | 100 | 1.333 | 0.001 | ↑ |
| NDUFB6 | NADH dehydrogenase | gi\|50755345 | *Gallus gallus* | 1 | 6 | 0.078 | 100 | 1.332 | 0.01 | ↑ |
| ABCB11 | bile salt export pump | gi\|363736219 | *Gallus gallus* | 10 | 11 | 0.087 | 97.78 | 1.329 | 0.007 | ↑ |
| MRC1L-A | MRC1L-A | gi\|452084954 | *Gallus gallus* | 14 | 22 | 0.122 | 99.93 | 1.328 | 0.001 | ↑ |
| NDUFS2 | NADH dehydrogenase | gi\|363745994 | *Gallus gallus* | 6 | 17 | 0.654 | 100 | 1.326 | 0.001 | ↑ |
| GRHPR | glyoxylate reductase/hydroxypyruvate reductase | gi\|363744954 | *Gallus gallus* | 13 | 67 | 0.699 | 100 | 1.326 | 0.001 | ↑ |
| ALDH3A2 | fatty aldehyde dehydrogenase | gi\|57525324 | *Gallus gallus* | 13 | 51 | 0.371 | 99.39 | 1.325 | 0.001 | ↑ |
| PGM1 | phosphoglucomutase-1 | gi\|84619526 | *Gallus gallus* | 8 | 17 | 0.136 | 99.83 | 1.322 | 0.001 | ↑ |
| ALDH7A1 | alpha-aminoadipic semialdehyde dehydrogenase isoform 2 | gi\|118104602 | *Gallus gallus* | 16 | 169 | 0.437 | 100 | 1.321 | 0.001 | ↑ |
| UBE4A | ubiquitin conjugation factor E4 A | gi\|71897157 | *Gallus gallus* | 1 | 1 | 0.035 | 100 | 1.32 | 0.036 | ↑ |
| FABP5 | fatty acid binding protein 5 (psoriasis-associated) | gi\|57530631 | *Gallus gallus* | 1 | 4 | 0.067 | 100 | 1.316 | 0.038 | ↑ |
| AIFM2 | apoptosis-inducing factor 2 | gi\|50749348 | *Gallus gallus* | 4 | 5 | 0.142 | 100 | 1.314 | 0.042 | ↑ |
| ADAM23 | disintegrin and metalloproteinase domain-containing protein 23 | gi\|223636298 | *Gallus gallus* | 1 | 6 | 0.015 | 100 | 1.314 | 0.005 | ↑ |
| PDIA4 | protein disulfide-isomerase A4 | gi\|57530768 | *Gallus gallus* | 28 | 126 | 0.592 | 99.68 | 1.314 | 0.001 | ↑ |
| RPL10A | 60S ribosomal protein L10a | gi\|50760435 | *Gallus gallus* | 7 | 22 | 0.415 | 100 | 1.303 | 0.001 | ↑ |
| LOC417013 | putative acyl-CoA dehydrogenase AidB-like | gi\|363740095 | *Gallus gallus* | 21 | 51 | 0.455 | 100 | 1.303 | 0.001 | ↑ |
| UBA3 | NEDD8-activating enzyme E1 catalytic subunit isoform 1 | gi\|363738827 | *Gallus gallus* | 3 | 6 | 0.084 | 100 | 1.294 | 0.017 | ↑ |
| CISD1 | CDGSH iron sulfur domain-containing protein 1 | gi\|314122187 | *Gallus gallus* | 3 | 14 | 0.444 | 100 | 1.293 | 0.006 | ↑ |
| apoAIV | apolipoprotein A-IV precursor | gi\|45384392 | *Gallus gallus* | 17 | 66 | 0.669 | 100 | 1.291 | 0.001 | ↑ |
| COL12A1 | collagen alpha-1(XII) chain precursor | gi\|45384318 | *Gallus gallus* | 19 | 30 | 0.082 | 99.07 | 1.291 | 0.001 | ↑ |
| CTH | cystathionine gamma-lyase | gi\|118094764 | *Gallus gallus* | 12 | 71 | 0.398 | 100 | 1.29 | 0.001 | ↑ |
| HSD17B4 | peroxisomal multifunctional enzyme type 2 | gi\|45384406 | *Gallus gallus* | 21 | 84 | 0.415 | 100 | 1.287 | 0.001 | ↑ |
| CMBL | carboxymethylenebutenolidase homolog isoform 3 | gi\|50734923 | *Gallus gallus* | 3 | 10 | 0.121 | 95.94 | 1.286 | 0.002 | ↑ |
| CROT | peroxisomal carnitine O-octanoyltransferase isoform 2 | gi\|118085713 | *Gallus gallus* | 6 | 19 | 0.104 | 100 | 1.283 | 0.001 | ↑ |
| HSDL2 | hydroxysteroid dehydrogenase-like protein 2-like isoform 1 | gi\|363744876 | *Gallus gallus* | 16 | 88 | 0.54 | 100 | 1.283 | 0.001 | ↑ |
| GLB1 | beta-galactosidase precursor | gi\|71896501 | *Gallus gallus* | 4 | 9 | 0.083 | 100 | 1.278 | 0.03 | ↑ |
| XPO7 | exportin-7 | gi\|61098426 | *Gallus gallus* | 3 | 9 | 0.027 | 100 | 1.276 | 0.022 | ↑ |
| COX4I1 | cytochrome c oxidase subunit 4 isoform 1, mitochondrial | gi\|71895513 | *Gallus gallus* | 10 | 61 | 0.789 | 100 | 1.275 | 0.001 | ↑ |
| NARS | asparaginyl-tRNA synthetase, cytoplasmic | gi\|71897197 | *Gallus gallus* | 4 | 11 | 0.072 | 100 | 1.273 | 0.002 | ↑ |
| ATG2B | autophagy-related protein 2 homolog B | gi\|363734236 | *Gallus gallus* | 2 | 8 | 0.013 | 99.9 | 1.272 | 0.002 | ↑ |
| ACAA2 | 3-ketoacyl-CoA thiolase, mitochondrial | gi\|57529492 | *Gallus gallus* | 20 | 140 | 0.909 | 100 | 1.271 | 0.001 | ↑ |
| PGM2 | phosphoglucomutase-2 | gi\|71897287 | *Gallus gallus* | 9 | 18 | 0.176 | 99.84 | 1.268 | 0.02 | ↑ |
| SUOX | Sulfite oxidase | gi\|28558813 | *Gallus gallus* | 8 | 30 | 0.207 | 100 | 1.267 | 0.001 | ↑ |
| CXorf57 | uncharacterized protein CXorf57 | gi\|363732680 | *Gallus gallus* | 1 | 6 | 0.01 | 86.89 | 1.266 | 0.03 | ↑ |
| CRAT | carnitine O-acetyltransferase | gi\|118099288 | *Gallus gallus* | 13 | 40 | 0.255 | 99.68 | 1.265 | 0.001 | ↑ |
| PHB2 | prohibitin-2 | gi\|124249322 | *Gallus gallus* | 11 | 56 | 0.422 | 100 | 1.263 | 0.001 | ↑ |
| GSTZ1 | maleylacetoacetate isomerase isoform 1 | gi\|118091881 | *Gallus gallus* | 6 | 25 | 0.4 | 100 | 1.261 | 0.001 | ↑ |
| GPX1 | glutathione peroxidase 1-like | gi\|363738644 | *Gallus gallus* | 7 | 37 | 0.675 | 100 | 1.259 | 0.001 | ↑ |
| PC | pyruvate carboxylase | gi\|45383466 | *Gallus gallus* | 50 | 897 | 0.624 | 100 | 1.259 | 0.001 | ↑ |
| NADK2 | NAD kinase domain-containing protein 1 | gi\|363744331 | *Gallus gallus* | 12 | 32 | 0.342 | 94.38 | 1.255 | 0.001 | ↑ |
| NPEPL1 | probable aminopeptidase NPEPL1 | gi\|118100855 | *Gallus gallus* | 10 | 12 | 0.3 | 100 | 1.253 | 0.014 | ↑ |
| NPEPL1 | glycogen debranching enzyme | gi\|118094283 | *Gallus gallus* | 16 | 19 | 0.122 | 99.74 | 1.251 | 0.006 | ↑ |
| CYP2D6 | cytochrome P450 2D3-like | gi\|307078128 | *Gallus gallus* | 4 | 12 | 0.116 | 100 | 1.25 | 0.001 | ↑ |
| A2ML4 | alpha-2-macroglobulin-like protein 1-like | gi\|363743392 | *Gallus gallus* | 16 | 26 | 0.135 | 99.72 | 1.246 | 0.001 | ↑ |
| BPIFB2 | ovoglobulinG2 type AA | gi\|385145527 | *Gallus gallus* | 1 | 8 | 0.109 | 100 | 1.245 | 0.013 | ↑ |
| GLUL | glutamine synthetase-like | gi\|326924776 | *Gallus gallus* | 12 | 46 | 0.516 | 99.45 | 1.244 | 0.001 | ↑ |
| GPD1L2 | glycerol-3-phosphate dehydrogenase 1-like protein | gi\|363736119 | *Gallus gallus* | 14 | 91 | 0.636 | 100 | 1.238 | 0.001 | ↑ |
| MRPS30 | 28S ribosomal protein S30, mitochondrial | gi\|45384394 | *Gallus gallus* | 3 | 14 | 0.082 | 100 | 1.237 | 0.014 | ↑ |
| COPA | coatomer subunit alpha | gi\|71897175 | *Gallus gallus* | 18 | 32 | 0.172 | 99.92 | 1.237 | 0.001 | ↑ |
| CBR1 | carbonyl reductase | gi\|71895267 | *Gallus gallus* | 15 | 96 | 0.797 | 99.64 | 1.235 | 0.001 | ↑ |
| SERPINC1 | antithrombin-III | gi\|363736402 | *Gallus gallus* | 9 | 29 | 0.203 | 100 | 1.234 | 0.001 | ↑ |
| Ndufv1 | NADH dehydrogenase | gi\|71896123 | *Gallus gallus* | 2 | 14 | 0.536 | 100 | 1.233 | 0.001 | ↑ |
| HPX | hemopexin | gi\|16805334 | *Gallus gallus* | 4 | 7 | 0.157 | 100 | 1.226 | 0.048 | ↑ |
| SULT1E1 | estrogen sulfotransferase | gi\|118090299 | *Gallus gallus* | 7 | 24 | 0.265 | 100 | 1.215 | 0.002 | ↑ |
| BSG | basigin isoform 1 precursor | gi\|347543825 | *Gallus gallus* | 6 | 30 | 0.222 | 100 | 1.213 | 0.006 | ↑ |
| PSAT1 | phosphoserine aminotransferase | gi\|363744581 | *Gallus gallus* | 15 | 57 | 0.435 | 100 | 1.213 | 0.001 | ↑ |
| ACOX1 | peroxisomal acyl-coenzyme A oxidase 1 | gi\|55741614 | *Gallus gallus* | 15 | 45 | 0.393 | 99.7 | 1.21 | 0.003 | ↑ |
| PPP2R2A | serine-threonine protein phosphatase 2A regulatory subunit | gi\|26984174 | *Gallus gallus* | 3 | 10 | 0.122 | 100 | 1.207 | 0.019 | ↑ |
| XPNPEP3 | probable Xaa-Pro aminopeptidase 3 | gi\|50728694 | *Gallus gallus* | 3 | 9 | 0.055 | 100 | 1.204 | 0.008 | ↑ |
| PGRMC1 | membrane-associated progesterone receptor component 1 | gi\|429903872 | *Gallus gallus* | 4 | 13 | 0.318 | 100 | 1.202 | 0.004 | ↑ |

Abbreviations: NCBInr Identity, Identity score of blast (NCBInr); NCBInr Accession, Matched accession of blast (NCBInr); NCBInr Description, Description of matched accession (NCBInr); Uniq_Pep_Num, Identified unique peptide number of protein; Uniq_Spec_Num, Identified unique spectrum number of protein.

**^#^** compared with control group, ↑ indicated up-regulated; ↓ indicated down-regulated.

Tendency: proteins expression changes at E19d than that at E14d in chicken embryo, ↑indicated up-regulated; ↓indicated down-regulated.
