# Supplementary material for: Proteomics reveals changes in hepatic proteins during chicken embryonic development: an alternative model to study human obesity
Source: BMC Genomics. 2018 Jan 8;19:29. doi: 10.1186/s12864-017-4427-6 (PMC5759888; doi:10.1186/s12864-017-4427-6)
Supplement: Supplementary file 3 — Differentially expressed proteins at H1d when compared to E19d in chicken embryos. (DOCX 68 kb) [file 12864_2017_4427_MOESM3_ESM.docx]

**Online additional file**

**Proteomics analysis reveals hepatic proteins changes during chicken embryonic development：An alternative model for human obesity study**

Mengling Peng, Shengnan Li, Qianqian He, Jinlong Zhao, Longlong Li, Haitian Ma*

**Additional Table 2.** Differentially expressed proteins at H1d when compared to E19d in chicken embryos

| Gene Ontology | NCBInr Description | NCBInr Accession | Species | Uniq_Pep _Num | Uniq_Spec_Num | Protein Coverage | NCBInr Identity | Ratio | P-value | Tendency |
| --- | --- | --- | --- | --- | --- | --- | --- | --- | --- | --- |
| BPIFB2 | ovoglobulinG2 type AA | gi\|385145527 | *Gallus gallus* | 1 | 8 | 0.109 | 100 | 0.784 | 0.018 | ↓ |
| FABPI | fatty acid-binding protein | gi\|56119000 | *Gallus gallus* | 4 | 19 | 0.326 | 100 | 0.622 | 0.001 | ↓ |
| FBXL12 | hepatic lectin | gi\|45382743 | *Gallus gallus* | 2 | 9 | 0.068 | 100 | 0.658 | 0.001 | ↓ |
| ACOT2L | acyl-coenzyme A thioesterase 2, mitochondrial-like | gi\|363734458 | *Gallus gallus* | 2 | 6 | 0.059 | 100 | 0.732 | 0.047 | ↓ |
| CAPN11 | CAPN1 [Gallus gallus] | gi\|209892841 | *Gallus gallus* | 2 | 4 | 0.044 | 100 | 0.684 | 0.005 | ↓ |
| CROT | PREDICTED: peroxisomal carnitine O-octanoyltransferase isoform 2 | gi\|118085713 | *Gallus gallus* | 6 | 19 | 0.104 | 100 | 0.761 | 0.002 | ↓ |
| RSPRY1 | RING finger and SPRY domain-containing protein 1 | gi\|50753502 | *Gallus gallus* | 1 | 3 | 0.014 | 100 | 0.655 | 0.023 | ↓ |
| SPINK7 | ovomucoid | gi\|209979542 | *Gallus gallus* | 2 | 5 | 0.157 | 100 | 0.676 | 0.005 | ↓ |
| HBBH | epsilon globin | gi\|71895591 | *Gallus gallus* | 4 | 10 | 0.816 | 100 | 0.769 | 0.001 | ↓ |
| MOGAT1 | 2-acylglycerol O-acyltransferase 1 | gi\|363737112 | *Gallus gallus* | 5 | 15 | 0.221 | 99.67 | 0.643 | 0.001 | ↓ |
| HPGD | 15-hydroxyprostaglandin dehydrogenase [NAD+] | gi\|50746505 | *Gallus gallus* | 6 | 26 | 0.26 | 100 | 0.782 | 0.001 | ↓ |
| LDHB | L-lactate dehydrogenase B chain | gi\|45383766 | *Gallus gallus* | 21 | 376 | 0.859 | 100 | 0.684 | 0.001 | ↓ |
| CATH1 | fowlicidin-1 | gi\|72003802 | *Gallus gallus* | 2 | 4 | 0.196 | 99.32 | 0.733 | 0.007 | ↓ |
| HBA1 | hemoglobin subunit alpha-A | gi\|52138655 | *Gallus gallus* | 8 | 313 | 0.838 | 100 | 0.574 | 0.001 | ↓ |
| COL14A1 | collagen XIV | gi\|288875 | *Gallus gallus* | 10 | 12 | 0.07 | 99.78 | 0.736 | 0.01 | ↓ |
| HBM | hemoglobin subunit alpha-D | gi\|52138645 | *Gallus gallus* | 10 | 410 | 0.922 | 100 | 0.639 | 0.001 | ↓ |
| NLN | neurolysin, mitochondrial | gi\|118103869 | *Gallus gallus* | 3 | 5 | 0.046 | 100 | 0.779 | 0.011 | ↓ |
| SCARB2 | lysosome membrane protein 2 | gi\|50746651 | *Gallus gallus* | 9 | 31 | 0.249 | 100 | 0.756 | 0.001 | ↓ |
| MELTF | melanotransferrin precursor | gi\|45383930 | *Gallus gallus* | 7 | 18 | 0.13 | 99.05 | 0.669 | 0.001 | ↓ |
| SULT | sulfotransferase | gi\|45384226 | *Gallus gallus* | 21 | 293 | 0.811 | 100 | 0.739 | 0.001 | ↓ |
| AKR1B10 | aldo-keto reductase family 1 member B10 | gi\|45382879 | *Gallus gallus* | 1 | 8 | 0.069 | 100 | 0.79 | 0.002 | ↓ |
| PTER | phosphotriesterase-related protein | gi\|50732421 | *Gallus gallus* | 3 | 8 | 0.104 | 100 | 0.765 | 0.024 | ↓ |
| GAPDH | glyceraldehyde-3-phosphate dehydrogenase | gi\|46048961 | *Gallus gallus* | 22 | 587 | 0.999 | 100 | 0.766 | 0.001 | ↓ |
| CYP2AC1 | cytochrome P450 2K1-like | gi\|118089176 | *Gallus gallus* | 18 | 60 | 0.482 | 100 | 0.757 | 0.001 | ↓ |
| AFP | alpha-fetoprotein | gi\|363733434 | *Gallus gallus* | 10 | 36 | 0.192 | 100 | 0.776 | 0.001 | ↓ |
| SLC25A20 | mitochondrial carnitine/acylcarnitine carrier protein | gi\|50754473 | *Gallus gallus* | 7 | 30 | 0.296 | 100 | 0.785 | 0.001 | ↓ |
| PCTPL | PCTP-like protein-like | gi\|50745555 | *Gallus gallus* | 5 | 19 | 0.263 | 100 | 0.798 | 0.004 | ↓ |
| CPE | carboxypeptidase E | gi\|363733143 | *Gallus gallus* | 1 | 2 | 0.016 | 99.44 | 0.1 | 0.001 | ↓ |
| HBZ | hemoglobin subunit pi | gi\|52138651 | *Gallus gallus* | 8 | 67 | 0.725 | 100 | 0.683 | 0.001 | ↓ |
| AvBD1 | gallinacin-1 | gi\|50404774 | *Gallus gallus* | 1 | 3 | 0.205 | 100 | 0.696 | 0.038 | ↓ |
| LY86 | lymphocyte antigen 86 precursor | gi\|52138689 | *Gallus gallus* | 1 | 2 | 0.075 | 99.38 | 0.584 | 0.022 | ↓ |
| GYG1 | glycogenin-1 | gi\|57529551 | *Gallus gallus* | 2 | 11 | 0.093 | 99.7 | 0.797 | 0.017 | ↓ |
| ATP8 | ATP synthase F0 subunit 8 | gi\|198401599 | *Gallus gallus* | 2 | 11 | 0.643 | 100 | 0.799 | 0.001 | ↓ |
| AGPAT3 | PR1-acyl-sn-glycerol-3-phosphate acyltransferase gamma | gi\|118083886 | *Gallus gallus* | 6 | 16 | 0.165 | 100 | 0.636 | 0.001 | ↓ |
| LYG2 | lysozyme g precursor | gi\|47825389 | *Gallus gallus* | 5 | 7 | 0.299 | 99.53 | 0.657 | 0.021 | ↓ |
| RSFR | ribonuclease homolog precursor | gi\|56118294 | *Gallus gallus* | 3 | 5 | 0.302 | 100 | 0.768 | 0.031 | ↓ |
| CA13 | carbonic anhydrase 13-like | gi\|363730730 | *Gallus gallus* | 3 | 5 | 0.154 | 100 | 0.721 | 0.038 | ↓ |
| TTN | connectin/titin | gi\|1513030 | *Gallus gallus* | 1 | 5 | 0.005 | 88.65 | 0.522 | 0.002 | ↓ |
| LDHA | L-lactate dehydrogenase A chain | gi\|45384208 | *Gallus gallus* | 6 | 27 | 0.262 | 99.7 | 0.727 | 0.001 | ↓ |
| TGFBR2 | TGF-beta receptor type-2 precursor | gi\|45382189 | *Gallus gallus* | 1 | 1 | 0.013 | 100 | 0.317 | 0.029 | ↓ |
| GSTA3 | glutathione S-transferase 3 | gi\|47604962 | *Gallus gallus* | 7 | 28 | 0.402 | 99.56 | 0.712 | 0.001 | ↓ |
| SURF4 | surfeit locus protein 4 | gi\|45383444 | *Gallus gallus* | 2 | 19 | 0.104 | 100 | 0.788 | 0.025 | ↓ |
| COMTD1 | catechol O-methyltransferase domain-containing protein 1 | gi\|363735497 | *Gallus gallus* | 5 | 8 | 0.199 | 100 | 0.739 | 0.012 | ↓ |
| CO1A2 | Collagen alpha-2(I) chain | gi\|5921192 | *Gallus gallus* | 9 | 15 | 0.112 | 100 | 0.766 | 0.01 | ↓ |
| PRKAR1A | cAMP-dependent protein kinase type I-alpha regulatory subunit | gi\|56119042 | *Gallus gallus* | 6 | 17 | 0.432 | 100 | 0.763 | 0.001 | ↓ |
| PLOD1 | procollagen-lysine,2-oxoglutarate 5-dioxygenase 1 precursor | gi\|54111425 | *Gallus gallus* | 2 | 5 | 0.027 | 100 | 0.713 | 0.002 | ↓ |
| HBBA | hemoglobin subunit beta | gi\|49169791 | *Gallus gallus* | 7 | 86 | 0.999 | 100 | 0.594 | 0.001 | ↓ |
| HBBR | hemoglobin subunit rho | gi\|52138683 | *Gallus gallus* | 1 | 6 | 0.435 | 100 | 0.759 | 0.002 | ↓ |
| CA2 | carbonic anhydrase 2 | gi\|46048696 | *Gallus gallus* | 8 | 26 | 0.469 | 100 | 0.733 | 0.001 | ↓ |
| MED24 | mediator of RNA polymerase II transcription subunit 24 | gi\|71897119 | *Gallus gallus* | 1 | 4 | 0.014 | 100 | 0.795 | 0.029 | ↓ |
| CES1L1 | fatty acyl-CoA hydrolase precursor, medium chain isoform 2 | gi\|363738173 | *Gallus gallus* | 7 | 35 | 0.395 | 98.02 | 0.742 | 0.001 | ↓ |
| HBE | hemoglobin subunit epsilon | gi\|126165290\| | *Gallus gallus* | 1 | 3 | 0.748 | 99.32 | 0.559 | 0.001 | ↓ |
| M126 | Protein MRP-126 | gi\|126659 | *Gallus gallus* | 2 | 2 | 0.143 | 100 | 0.637 | 0.049 | ↓ |
| AADAC | arylacetamide deacetylase | gi\|118095370 | *Gallus gallus* | 17 | 87 | 0.613 | 100 | 0.797 | 0.001 | ↓ |
| GPD1L | glycerol-3-phosphate dehydrogenase 1-like | gi\|50732786 | *Gallus gallus* | 2 | 16 | 0.076 | 100 | 0.737 | 0.001 | ↓ |
| P22 | calcium-binding protein | gi\|46048671 | *Gallus gallus* | 5 | 27 | 0.26 | 100 | 0.754 | 0.001 | ↓ |
| HELZ2 | peroxisomal proliferator-activated receptor A-interacting complex 285 kDa protein | gi\|118100744 | *Gallus gallus* | 2 | 4 | 0.006 | 100 | 0.327 | 0.03 | ↓ |
| LBFABP | fatty acid-binding protein, liver | gi\|45382869 | *Gallus gallus* | 4 | 70 | 0.389 | 100 | 3.597 | 0.001 | ↑ |
| PPP6R3 | serine/threonine-protein phosphatase 6 regulatory subunit 3 | gi\|71894719 | *Gallus gallus* | 1 | 1 | 0.011 | 100 | 2.791 | 0.032 | ↑ |
| TEKT4 | tektin-4 | gi\|50755635 | *Gallus gallus* | 1 | 1 | 0.048 | 100 | 2.779 | 0.022 | ↑ |
| FABP1 | fatty acid-binding protein, liver | gi\|45383728 | *Gallus gallus* | 6 | 30 | 0.614 | 100 | 2.769 | 0.001 | ↑ |
| IGLL1 | Ig light chain precursor | gi\|212195 | *Gallus gallus* | 1 | 17 | 0.194 | 100 | 1.954 | 0.001 | ↑ |
| 1 SV | Apovitellenin-1 | gi\|114078 | *Gallus gallus* | 3 | 4 | 0.434 | 100 | 1.89 | 0.001 | ↑ |
| XDH | xanthine dehydrogenase/oxidase | gi\|46048759 | *Gallus gallus* | 7 | 10 | 0.06 | 100 | 1.88 | 0.001 | ↑ |
| PAICS | multifunctional protein ADE2 | gi\|45382959 | *Gallus gallus* | 20 | 79 | 0.641 | 100 | 1.801 | 0.001 | ↑ |
| XPNPEP3 | probable Xaa-Pro aminopeptidase 3 | gi\|50728694 | *Gallus gallus* | 3 | 9 | 0.055 | 100 | 1.794 | 0.004 | ↑ |
| DBI | Acyl-CoA-binding protein | gi\|14194444 | *Gallus gallus* | 3 | 36 | 0.488 | 100 | 1.751 | 0.001 | ↑ |
| FKBP5 | peptidyl-prolyl cis-trans isomerase FKBP5 | gi\|53749682 | *Gallus gallus* | 5 | 15 | 0.134 | 100 | 1.746 | 0.001 | ↑ |
| CYP1A2 | cytochrome P450 1A5 | gi\|45384068 | *Gallus gallus* | 1 | 1 | 0.053 | 100 | 1.721 | 0.03 | ↑ |
| MCM4 | DNA replication licensing factor mcm4 | gi\|118086936 | *Gallus gallus* | 7 | 11 | 0.087 | 100 | 1.715 | 0.001 | ↑ |
| IYD | iodotyrosine dehalogenase 1 | gi\|118088376 | *Gallus gallus* | 1 | 2 | 0.034 | 100 | 1.664 | 0.023 | ↑ |
| NDUFB1 | NADH dehydrogenase [ubiquinone] 1 beta subcomplex subunit 1 | gi\|147906350 | *Gallus gallus* | 1 | 3 | 0.186 | 100 | 1.658 | 0.01 | ↑ |
| PPAT | amidophosphoribosyltransferase precursor | gi\|52345390 | *Gallus gallus* | 15 | 25 | 0.38 | 99.8 | 1.658 | 0.001 | ↑ |
| MIF | Macrophage migration inhibitory factor | gi\|400257 | *Gallus gallus* | 3 | 18 | 0.313 | 100 | 1.651 | 0.001 | ↑ |
| HMGCS1 | hydroxymethylglutaryl-CoA synthase, cytoplasmic | gi\|45382279 | *Gallus gallus* | 7 | 15 | 0.18 | 99.23 | 1.651 | 0.001 | ↑ |
| AP3M1 | AP-3 complex subunit mu-1 | gi\|71895033 | *Gallus gallus* | 2 | 2 | 0.079 | 100 | 1.627 | 0.035 | ↑ |
| PFKFB4 | 6-phosphofructo-2-kinase/fructose-2,6-bisphosphatase | gi\|71895485 | *Gallus gallus* | 8 | 16 | 0.2 | 100 | 1.622 | 0.001 | ↑ |
| A2ML4 | alpha-2-macroglobulin-like protein 1-like | gi\|363743392 | *Gallus gallus* | 16 | 26 | 0.135 | 99.72 | 1.592 | 0.001 | ↑ |
| POR | NADPH--cytochrome P450 reductase | gi\|307775405 | *Gallus gallus* | 14 | 49 | 0.257 | 100 | 1.591 | 0.001 | ↑ |
| SLC26A5 | prestin | gi\|118405150 | *Gallus gallus* | 2 | 4 | 0.044 | 100 | 1.578 | 0.022 | ↑ |
| FASN | fatty acid synthase | gi\|319655768 | *Gallus gallus* | 15 | 26 | 0.08 | 100 | 1.576 | 0.001 | ↑ |
| CYP3A5 | cytochrome P450 A 37 | gi\|48976101 | *Gallus gallus* | 8 | 14 | 0.157 | 99.8 | 1.558 | 0.001 | ↑ |
| COX7C | cytochrome c oxidase subunit 7C, mitochondrial-like | gi\|118104378 | *Gallus gallus* | 1 | 4 | 0.143 | 100 | 1.556 | 0.001 | ↑ |
| SYT1 | synaptotagmin-1 | gi\|45384016 | *Gallus gallus* | 3 | 6 | 0.078 | 100 | 1.531 | 0.032 | ↑ |
| MRPS36 | 8S ribosomal protein S36, mitochondrial | gi\|363744215 | *Gallus gallus* | 1 | 7 | 0.126 | 100 | 1.517 | 0.004 | ↑ |
| CDV3 | protein CDV3 homolog | gi\|71896619 | *Gallus gallus* | 1 | 3 | 0.039 | 100 | 1.513 | 0.002 | ↑ |
| FTH1 | ferritin heavy chain | gi\|45384172 | *Gallus gallus* | 5 | 9 | 0.261 | 100 | 1.51 | 0.001 | ↑ |
| SCUBE2 | signal peptide, CUB domain, EGF-like 2 | gi\|363734260 | *Gallus gallus* | 1 | 7 | 0.008 | 100 | 1.498 | 0.001 | ↑ |
| GLDC | glycine dehydrogenase， mitochondrial precursor | gi\|45383510 | *Gallus gallus* | 11 | 19 | 0.161 | 99.21 | 1.492 | 0.001 | ↑ |
| MYDGF | uncharacterized protein LOC420161 precursor | gi\|57530610 | *Gallus gallus* | 1 | 3 | 0.054 | 100 | 1.471 | 0.005 | ↑ |
| UQCRFS1 | cytochrome b-c1 complex subunit Rieske, mitochondrial precursor | gi\|57524866 | *Gallus gallus* | 4 | 17 | 0.276 | 100 | 1.464 | 0.001 | ↑ |
| UGP2 | hypothetical protein RCJMB04_8o6 | gi\|53130600 | *Gallus gallus* | 14 | 51 | 0.343 | 99.8 | 1.443 | 0.001 | ↑ |
| APOA1 | apolipoprotein A-I preproprotein | gi\|45382961 | *Gallus gallus* | 20 | 241 | 0.837 | 100 | 1.44 | 0.001 | ↑ |
| GFM1 | elongation factor G, mitochondrial | gi\|118095339 | *Gallus gallus* | 9 | 25 | 0.14 | 100 | 1.436 | 0.001 | ↑ |
| ACSL5 | long-chain-fatty-acid--CoA ligase 5 | gi\|71895089 | *Gallus gallus* | 9 | 23 | 0.177 | 100 | 1.43 | 0.001 | ↑ |
| TOP1 | DNA topoisomerase 1 | gi\|45384130 | *Gallus gallus* | 2 | 4 | 0.024 | 100 | 1.415 | 0.034 | ↑ |
| ANPEP | aminopeptidase N | gi\|45382361 | *Gallus gallus* | 15 | 35 | 0.187 | 100 | 1.401 | 0.001 | ↑ |
| GSTA3 | glutathione S-transferase | gi\|49169816 | *Gallus gallus* | 5 | 15 | 0.575 | 100 | 1.397 | 0.001 | ↑ |
| ACSF2 | acyl-CoA synthetase family member 2, mitochondrial | gi\|118099923 | *Gallus gallus* | 7 | 12 | 0.151 | 99.29 | 1.396 | 0.013 | ↑ |
| HPD | 4-hydroxyphenylpyruvate dioxygenase | gi\|363739843 | *Gallus gallus* | 18 | 163 | 0.616 | 100 | 1.395 | 0.001 | ↑ |
| SERPINB14 | Ovalbumin | gi\|129293 | *Gallus gallus* | 11 | 157 | 0.443 | 100 | 1.385 | 0.001 | ↑ |
| LOC415662 | uncharacterized oxidoreductase | gi\|363738106 | *Gallus gallus* | 3 | 16 | 0.556 | 99.23 | 1.378 | 0.002 | ↑ |
| RBP4 | retinol-binding protein 4 precursor | gi\|45382541 | *Gallus gallus* | 3 | 7 | 0.194 | 100 | 1.373 | 0.031 | ↑ |
| NT5C2 | cytosolic purine 5'-nucleotidase | gi\|71895075 | *Gallus gallus* | 9 | 23 | 0.225 | 100 | 1.369 | 0.001 | ↑ |
| SSB | Sjogren syndrome antigen B (autoantigen La) isoform 1 | gi\|302488427 | *Gallus gallus* | 5 | 13 | 0.139 | 99.75 | 1.369 | 0.004 | ↑ |
| MCM5 | DNA replication licensing factor MCM5 | gi\|57525409 | *Gallus gallus* | 7 | 8 | 0.139 | 100 | 1.364 | 0.009 | ↑ |
| PGM2 | phosphoglucomutase-2 | gi\|71897287 | *Gallus gallus* | 9 | 18 | 0.176 | 99.84 | 1.36 | 0.001 | ↑ |
| DDT | D-dopachrome decarboxylase | gi\|71897241 | *Gallus gallus* | 6 | 46 | 0.839 | 99.15 | 1.358 | 0.001 | ↑ |
| COL18A1 | collagen, type XVIII, alpha 1 precursor | gi\|45383788 | *Gallus gallus* | 6 | 9 | 0.063 | 100 | 1.344 | 0.007 | ↑ |
| ADSL | adenysuccinate lyase | gi\|54695133 | *Gallus gallus* | 1 | 10 | 0.563 | 100 | 1.342 | 0.031 | ↑ |
| CALR3 | calreticulin | gi\|118103332 | *Gallus gallus* | 1 | 16 | 0.017 | 100 | 1.341 | 0.001 | ↑ |
| GATM | Glycine amidinotransferase | gi\|308191432 | *Gallus gallus* | 19 | 123 | 0.6 | 99.76 | 1.332 | 0.001 | ↑ |
| SLC9A3R2 | Na(+)/H(+) exchange regulatory cofactor NHE-RF2 | gi\|363739404 | *Gallus gallus* | 2 | 5 | 0.093 | 100 | 1.33 | 0.013 | ↑ |
| APOH | beta-2-glycoprotein 1 | gi\|118099767 | *Gallus gallus* | 5 | 10 | 0.168 | 100 | 1.323 | 0.01 | ↑ |
| SLC25A4 | ADP/ATP translocase 1 | gi\|57530120 | *Gallus gallus* | 2 | 6 | 0.265 | 100 | 1.321 | 0.022 | ↑ |
| PCNA | proliferating cell nuclear antigen | gi\|45383776 | *Gallus gallus* | 7 | 14 | 0.366 | 100 | 1.315 | 0.004 | ↑ |
| STRAP | serine-threonine kinase receptor-associated protein | gi\|347800736 | *Gallus gallus* | 5 | 13 | 0.166 | 100 | 1.309 | 0.001 | ↑ |
| CYCS | cytochrome c | gi\|118405198 | *Gallus gallus* | 9 | 65 | 1 | 100 | 1.301 | 0.001 | ↑ |
| AKR1D1 | 3-oxo-5-beta-steroid 4-dehydrogenase isoform 2 | gi\|363728195 | *Gallus gallus* | 11 | 37 | 0.505 | 100 | 1.297 | 0.001 | ↑ |
| ROCK1 | rho-associated protein kinase 1 | gi\|313661353 | *Gallus gallus* | 2 | 4 | 0.029 | 100 | 1.288 | 0.047 | ↑ |
| GNE | hypothetical protein RCJMB04_6o2 | gi\|60098713 | *Gallus gallus* | 3 | 5 | 0.048 | 100 | 1.287 | 0.018 | ↑ |
| SULT1C3 | sulfotransferase 1C1 | gi\|45382969 | *Gallus gallus* | 16 | 89 | 0.717 | 100 | 1.286 | 0.001 | ↑ |
| 1 | Histone H2A-III | gi\|462231 | *Gallus gallus* | 3 | 17 | 0.465 | 100 | 1.278 | 0.004 | ↑ |
| COMMD6 | COMM domain-containing protein 6 | gi\|363729141 | *Gallus gallus* | 2 | 4 | 0.135 | 100 | 1.277 | 0.039 | ↑ |
| SF3B1 | splicing factor 3B subunit 1 | gi\|363735880 | *Gallus gallus* | 4 | 11 | 0.037 | 100 | 1.273 | 0.011 | ↑ |
| OVALY | ovalbumin-related protein Y | gi\|71897377 | *Gallus gallus* | 7 | 13 | 0.229 | 100 | 1.268 | 0.031 | ↑ |
| AKR1A1 | alcohol dehydrogenase [NADP(+)] | gi\|57529654 | *Gallus gallus* | 8 | 24 | 0.275 | 99.69 | 1.268 | 0.001 | ↑ |
| RPL7A | 60S ribosomal protein L7a | gi\|52138653 | *Gallus gallus* | 8 | 19 | 0.389 | 100 | 1.268 | 0.002 | ↑ |
| RPL19 | 60S ribosomal protein L19 | gi\|71896335 | *Gallus gallus* | 2 | 7 | 0.144 | 100 | 1.267 | 0.03 | ↑ |
| PRDX1 | peroxiredoxin-1 | gi\|429836849 | *Gallus gallus* | 9 | 97 | 0.724 | 100 | 1.265 | 0.001 | ↑ |
| CALR | calreticulin | gi\|44969651 | *Gallus gallus* | 15 | 84 | 0.545 | 100 | 1.265 | 0.001 | ↑ |
| HSPH1 | heat shock 105kDa | gi\|228008358 | *Gallus gallus* | 4 | 9 | 0.063 | 98.94 | 1.262 | 0.044 | ↑ |
| ACOX1 | peroxisomal acyl-coenzyme A oxidase 1 | gi\|55741614 | *Gallus gallus* | 15 | 45 | 0.393 | 99.7 | 1.259 | 0.001 | ↑ |
| HSPA5 | 78 kDa glucose-regulated protein precursor | gi\|45382769 | *Gallus gallus* | 23 | 234 | 0.518 | 100 | 1.256 | 0.001 | ↑ |
| PRMT5 | hypothetical protein RCJMB04_14b8 | gi\|53132882 | *Gallus gallus* | 4 | 5 | 0.076 | 100 | 1.252 | 0.029 | ↑ |
| IMPDH2 | inosine-5'-monophosphate dehydrogenase 2 | gi\|71895387 | *Gallus gallus* | 7 | 11 | 0.21 | 100 | 1.25 | 0.006 | ↑ |
| EFHD1 | EF-hand domain-containing protein D1 | gi\|72535161 | *Gallus gallus* | 3 | 9 | 0.151 | 100 | 1.243 | 0.011 | ↑ |
| PSMD9 | 26S proteasome non-ATPase regulatory subunit 9 | gi\|57525182 | *Gallus gallus* | 3 | 4 | 0.164 | 99.52 | 1.243 | 0.041 | ↑ |
| C11ORF54 | chromosome 1 open reading frame, human C11orf54 | gi\|471434827 | *Gallus gallus* | 10 | 37 | 0.5 | 100 | 1.243 | 0.001 | ↑ |
| YVCT | probable 2-ketogluconate reductase-like, partial | gi\|363745151 | *Gallus gallus* | 2 | 7 | 0.195 | 100 | 1.24 | 0.013 | ↑ |
| ABAT | 4-aminobutyrate aminotransferase, mitochondrial | gi\|118098116 | *Gallus gallus* | 8 | 17 | 0.17 | 100 | 1.239 | 0.001 | ↑ |
| RPS3A | ribosomal protein S3A | gi\|129270064 | *Gallus gallus* | 14 | 67 | 0.572 | 100 | 1.239 | 0.001 | ↑ |
| RPLP1 | 60S acidic ribosomal protein P1 | gi\|45384350 | *Gallus gallus* | 1 | 10 | 0.14 | 100 | 1.234 | 0.016 | ↑ |
| PABPC1 | polyadenylate-binding protein 1 | gi\|71896197 | *Gallus gallus* | 12 | 42 | 0.316 | 100 | 1.233 | 0.001 | ↑ |
| EEF2 | elongation factor 2 | gi\|45382453 | *Gallus gallus* | 34 | 276 | 0.526 | 99.77 | 1.231 | 0.001 | ↑ |
| OGDH | 2-oxoglutarate dehydrogenase, mitochondrial | gi\|71897293 | *Gallus gallus* | 13 | 27 | 0.168 | 100 | 1.229 | 0.001 | ↑ |
| MRPL40 | 39S ribosomal protein L40, mitochondrial-like | gi\|363739973 | *Gallus gallus* | 5 | 8 | 0.337 | 100 | 1.226 | 0.026 | ↑ |
| SHMT1 | serine hydroxymethyltransferase, cytosolic | gi\|363739376 | *Gallus gallus* | 17 | 115 | 0.451 | 99.79 | 1.226 | 0.001 | ↑ |
| TPT1 | translationally-controlled tumor protein homolog | gi\|45382329 | *Gallus gallus* | 2 | 16 | 0.105 | 100 | 1.225 | 0.001 | ↑ |
| HAL | histidine ammonia-lyase | gi\|45383354 | *Gallus gallus* | 12 | 31 | 0.248 | 100 | 1.225 | 0.001 | ↑ |
| ATP5H | ATP synthase subunit d, mitochondrial isoform 1 | gi\|118099965 | *Gallus gallus* | 10 | 86 | 0.759 | 100 | 1.225 | 0.001 | ↑ |
| 1 SV | Histone H1 | gi\|121944 | *Gallus gallus* | 5 | 11 | 0.225 | 100 | 1.22 | 0.013 | ↑ |
| RPL18A | 60S ribosomal protein L18a, partial | gi\|363727621 | *Gallus gallus* | 3 | 6 | 0.196 | 100 | 1.218 | 0.035 | ↑ |
| LOC100859645 | glutathione S-transferase-like | gi\|363732251 | *Gallus gallus* | 2 | 6 | 0.303 | 100 | 1.216 | 0.047 | ↑ |
| PLG | plasminogen | gi\|118088308 | *Gallus gallus* | 9 | 13 | 0.132 | 100 | 1.216 | 0.021 | ↑ |
| TOMM70A | mitochondrial import receptor subunit TOM70 [Gallus gallus] | gi\|50729660 | *Gallus gallus* | 8 | 13 | 0.139 | 100 | 1.212 | 0.011 | ↑ |
| MANF | putative RNA-binding protein 15B | gi\|363738498 | *Gallus gallus* | 5 | 10 | 0.383 | 100 | 1.211 | 0.001 | ↑ |
| RPS2 | 40S ribosomal protein S2 | gi\|461496478 | *Gallus gallus* | 7 | 20 | 0.266 | 100 | 1.21 | 0.004 | ↑ |
| ERP29 | endoplasmic reticulum resident protein 29 precursor | gi\|444741647 | *Gallus gallus* | 6 | 32 | 0.261 | 100 | 1.209 | 0.001 | ↑ |
| PSMD7 | hypothetical protein RCJMB04_7b3 | gi\|53130330 | *Gallus gallus* | 4 | 7 | 0.155 | 100 | 1.205 | 0.011 | ↑ |

Abbreviations: NCBInr Identity, Identity score of blast (NCBInr); NCBInr Accession, Matched accession of blast (NCBInr); NCBInr Description, Description of matched accession (NCBInr); Uniq_Pep_Num, Identified unique peptide number of protein; Uniq_Spec_Num, Identified unique spectrum number of protein.

**^#^** compared with control group, ↑ indicated up-regulated; ↓ indicated down-regulated.

Tendency: proteins expression changes at H1d than that at E19d in chicken embryo, ↑indicated up-regulated; ↓indicated down-regulated.
