# Supplementary material for: Proteomics reveals changes in hepatic proteins during chicken embryonic development: an alternative model to study human obesity
Source: BMC Genomics. 2018 Jan 8;19:29. doi: 10.1186/s12864-017-4427-6 (PMC5759888; doi:10.1186/s12864-017-4427-6)
Supplement: Supplementary file 4 — The overlapping of differentially expressed abundance proteins on both comparisons in chicken embryos. (DOCX 24 kb) [file 12864_2017_4427_MOESM4_ESM.docx]

**Online Additional file**

**Proteomics analysis reveals hepatic proteins changes during chicken embryonic development：An alternative model for human obesity study**

Mengling Peng, Shengnan Li, Qianqian He, Jinlong Zhao, Longlong Li, Haitian Ma*

**Additional Table 3.** The overlapping of differentially expressed abundance proteins on both comparisons in chicken embryos

| Gene Ontology | NCBInr Description | Gene Ontology | NCBInr Description |
| --- | --- | --- | --- |
| HMGCS1 | hydroxymethylglutaryl-CoA synthase, cytoplasmic | SCARB2 | lysosome membrane protein 2 |
| XPNPEP3 | probable Xaa-Pro aminopeptidase 3 | SLC25A20 | mitochondrial carnitine/acylcarnitine carrier protein |
| A2ML4 | alpha-2-macroglobulin-like protein 1-like | SPINK7 | ovomucoid |
| ACOX1 | peroxisomal acyl-coenzyme A oxidase 1 | SURF4 | surfeit locus protein 4 |
| AKR1D1 | 3-oxo-5-beta-steroid 4-dehydrogenase isoform 2 | AKR1A1 | alcohol dehydrogenase |
| NT5C2 | cytosolic purine 5'-nucleotidase | ATP5H | ATP synthase subunit d, mitochondrial isoform 1 |
| OVALY | ovalbumin-related protein Y | CALR3 | calreticulin |
| PFKFB4 | 6-phosphofructo-2-kinase/fructose-2,6-bisphosphatase | ERP29 | endoplasmic reticulum resident protein 29 precursor |
| PGM2 | phosphoglucomutase-2 | GATM | Glycine amidinotransferase, mitochondrial |
| SERPINB14 | Ovalbumin | IMPDH2 | inosine-5'-monophosphate dehydrogenase 2 |
| AFP | alpha-fetoprotein | MCM4 | DNA replication licensing factor mcm4 |
| HBBR | hemoglobin subunit rho | MCM5 | DNA replication licensing factor MCM5 |
| AGPAT3 | PR1-acyl-sn-glycerol-3-phosphate acyltransferase gamma | MRPS36 | 28S ribosomal protein S36, mitochondrial |
| ATP8 | ATP synthase F0 subunit 8 | PABPC1 | polyadenylate-binding protein 1 |
| BPIFB2 | ovoglobulinG2 type AA | PCNA | proliferating cell nuclear antigen |
| CA2 | carbonic anhydrase 2 | PSMD9 | 26S proteasome non-ATPase regulatory subunit 9 |
| CROT | PREDICTED: peroxisomal carnitine O-octanoyltransferase isoform 2 | RBP4 | retinol-binding protein 4 precursor |
| FBXL12 | hepatic lectin | RPL7A | 60S ribosomal protein L7a |
| GPD1L | glycerol-3-phosphate dehydrogenase 1-like | RPLP1 | 60S acidic ribosomal protein P1 |
| HBA1 | hemoglobin subunit alpha-A | SCUBE2 | signal peptide, CUB domain, EGF-like 2 |
| HBBA | hemoglobin subunit beta | SSB | Sjogren syndrome antigen B (autoantigen La) isoform 1 |
| LDHA | L-lactate dehydrogenase A chain | STRAP | serine-threonine kinase receptor-associated protein |
| MOGAT1 | 2-acylglycerol O-acyltransferase 1 | SYT1 | synaptotagmin-1 |
| RSFR | ribonuclease homolog precursor | TPT1 | translationally-controlled tumor protein homolog |
| RSPRY1 | RING finger and SPRY domain-containing protein 1 |  |  |

Abbreviations: NCBInr Description, Description of matched accession (NCBInr)
