# Supplementary material for: Proteomics reveals changes in hepatic proteins during chicken embryonic development: an alternative model to study human obesity
Source: BMC Genomics. 2018 Jan 8;19:29. doi: 10.1186/s12864-017-4427-6 (PMC5759888; doi:10.1186/s12864-017-4427-6)
Supplement: Supplementary file 5 — Differentially expressed proteins with no annotated functions at E19d when compared to E14d in chicken embryos. (DOCX 41 kb) [file 12864_2017_4427_MOESM5_ESM.docx]

**Online Additional file**

**Proteomics analysis reveals hepatic proteins changes during chicken embryonic development：An alternative model for human obesity study**

Mengling Peng, Shengnan Li, Qianqian He, Jinlong Zhao, Longlong Li, Haitian Ma*

**Additional Table 4.** Differentially expressed proteins with no annotated functions at E19d when compared to E14d in chicken embryos

| Gene Ontology | NCBInr Description | NCBInr Accession | Species | Uniq_Pep _Num | Uniq_Spec_Num | Protein Coverage | NCBInr Identity | Ratio | P-value | Tendency |
| --- | --- | --- | --- | --- | --- | --- | --- | --- | --- | --- |
| LOC768709 | uncharacterized protein LOC768709 | gi\|118104643 | *Gallus gallus* | 2 | 3 | 0.13 | 100 | 1.731 | 0.003 | ↑ |
| A2M | alpha-2-macroglobulin | gi\|363728304 | *Gallus gallus* | 9 | 18 | 0.165 | 98.82 | 1.723 | 0.001 | ↑ |
| PIT 54 | PIT 54 protein precursor | gi\|46395491 | *Gallus gallus* | 10 | 30 | 0.328 | 100 | 1.709 | 0.001 | ↑ |
| RSPRY1 | RING finger and SPRY domain-containing protein 1 | gi\|50753502 | *Gallus gallus* | 1 | 3 | 0.014 | 98.34 | 1.685 | 0.006 | ↑ |
| SLC25A20 | mitochondrial carnitine/acylcarnitine carrier protein | gi\|50754473 | *Gallus gallus* | 7 | 30 | 0.296 | 100 | 1.668 | 0.001 | ↑ |
| SPINK7 | ovomucoid | gi\|209979542 | *Gallus gallus* | 2 | 5 | 0.157 | 98.52 | 1.652 | 0.016 | ↑ |
| SCARB2 | lysosome membrane protein 2 | gi\|50746651 | *Gallus gallus* | 9 | 31 | 0.249 | 100 | 1.622 | 0.001 | ↑ |
| TMEM30A | cell cycle control protein 50A | gi\|61097955 | *Gallus gallus* | 1 | 4 | 0.022 | 100 | 1.551 | 0.001 | ↑ |
| SRPRB | hypothetical protein RCJMB04_19k4 | gi\|53133718 | *Gallus gallus* | 4 | 6 | 0.182 | 99.03 | 1.545 | 0.017 | ↑ |
| PTCD1 | pentatricopeptide repeat-containing protein 1 | gi\|363739557 | *Gallus gallus* | 1 | 9 | 0.016 | 100 | 1.508 | 0.001 | ↑ |
| LOC100857820 | uncharacterized oxidoreductase C663.09c-like | gi\|363738114 | *Gallus gallus* | 2 | 7 | 0.581 | 100 | 1.5 | 0.001 | ↑ |
| ARL1 | ADP-ribosylation factor-like protein 1 | gi\|125628642 | *Gallus gallus* | 1 | 7 | 0.05 | 99.82 | 1.487 | 0.001 | ↑ |
| RSFR | ribonuclease homolog precursor | gi\|56118294 | *Gallus gallus* | 3 | 5 | 0.302 | 100 | 1.47 | 0.005 | ↑ |
| SERPINB14 | Ovalbumin | gi\|129293 | *Gallus gallus* | 11 | 157 | 0.443 | 100 | 1.432 | 0.001 | ↑ |
| DNAJC13 | dnaJ homolog subfamily C member 13 | gi\|118085986 | *Gallus gallus* | 4 | 6 | 0.02 | 100 | 1.414 | 0.034 | ↑ |
| Fgg | fibrinogen, gamma chain precursor | gi\|45384500 | *Gallus gallus* | 13 | 34 | 0.372 | 100 | 1.392 | 0.001 | ↑ |
| MTTP | microsomal triglyceride transfer protein large subunit precursor | gi\|157954041 | *Gallus gallus* | 28 | 122 | 0.456 | 100 | 1.392 | 0.001 | ↑ |
| SLC25A13 | calcium-binding mitochondrial carrier protein Aralar2 | gi\|61098440 | *Gallus gallus* | 13 | 38 | 0.288 | 98.83 | 1.378 | 0.001 | ↑ |
| OVALY | ovalbumin-related protein Y | gi\|71897377 | *Gallus gallus* | 7 | 13 | 0.229 | 100 | 1.367 | 0.008 | ↑ |
| SPINK5 | ovoinhibitor precursor | gi\|71895337 | *Gallus gallus* | 4 | 6 | 0.106 | 100 | 1.367 | 0.034 | ↑ |
| NIT2 | omega-amidase NIT2 isoform 2 | gi\|363728462 | *Gallus gallus* | 12 | 26 | 0.611 | 100 | 1.362 | 0.001 | ↑ |
| TMED5 | transmembrane emp24 domain-containing protein 5 precursor | gi\|56118980 | *Gallus gallus* | 3 | 5 | 0.142 | 100 | 1.348 | 0.026 | ↑ |
| Mtch2 | mitochondrial carrier homolog 2 | gi\|45382213 | *Gallus gallus* | 5 | 36 | 0.181 | 100 | 1.346 | 0.001 | ↑ |
| UBE4A | ubiquitin conjugation factor E4 A | gi\|71897157 | *Gallus gallus* | 1 | 1 | 0.035 | 100 | 1.32 | 0.036 | ↑ |
| ADAM23 | disintegrin and metalloproteinase domain-containing protein 23 | gi\|223636298 | *Gallus gallus* | 1 | 6 | 0.015 | 100 | 1.314 | 0.005 | ↑ |
| AIFM2 | apoptosis-inducing factor 2 | gi\|50749348 | *Gallus gallus* | 4 | 5 | 0.142 | 100 | 1.314 | 0.042 | ↑ |
| UBA3 | NEDD8-activating enzyme E1 catalytic subunit isoform 1 | gi\|363738827 | *Gallus gallus* | 3 | 6 | 0.084 | 100 | 1.294 | 0.017 | ↑ |
| CISD1 | CDGSH iron sulfur domain-containing protein 1 | gi\|314122187 | *Gallus gallus* | 3 | 14 | 0.444 | 100 | 1.293 | 0.006 | ↑ |
| COL12A1 | collagen alpha-1(XII) chain precursor | gi\|45384318 | *Gallus gallus* | 19 | 30 | 0.082 | 99.07 | 1.291 | 0.001 | ↑ |
| HSDL2 | hydroxysteroid dehydrogenase-like protein 2-like isoform 1 | gi\|363744876 | *Gallus gallus* | 16 | 88 | 0.54 | 100 | 1.283 | 0.001 | ↑ |
| GLB1 | beta-galactosidase precursor | gi\|71896501 | *Gallus gallus* | 4 | 9 | 0.083 | 100 | 1.278 | 0.03 | ↑ |
| XPO7 | exportin-7 | gi\|61098426 | *Gallus gallus* | 3 | 9 | 0.027 | 100 | 1.276 | 0.022 | ↑ |
| ATG2B | autophagy-related protein 2 homolog B | gi\|363734236 | *Gallus gallus* | 2 | 8 | 0.013 | 99.9 | 1.272 | 0.002 | ↑ |
| CXorf57 | uncharacterized protein CXorf57 | gi\|363732680 | *Gallus gallus* | 1 | 6 | 0.01 | 86.89 | 1.266 | 0.03 | ↑ |
| GPX1 | glutathione peroxidase 1-like | gi\|363738644 | *Gallus gallus* | 7 | 37 | 0.675 | 100 | 1.259 | 0.001 | ↑ |
| NPEPL1 | probable aminopeptidase NPEPL1 | gi\|118100855 | *Gallus gallus* | 10 | 12 | 0.3 | 100 | 1.253 | 0.014 | ↑ |
| A2ML4 | alpha-2-macroglobulin-like protein 1-like | gi\|363743392 | *Gallus gallus* | 16 | 26 | 0.135 | 99.72 | 1.246 | 0.001 | ↑ |
| BPIFB2 | ovoglobulinG2 type AA | gi\|385145527 | *Gallus gallus* | 1 | 8 | 0.109 | 100 | 1.245 | 0.013 | ↑ |
| COPA | coatomer subunit alpha | gi\|71897175 | *Gallus gallus* | 18 | 32 | 0.172 | 99.92 | 1.237 | 0.001 | ↑ |
| SERPINC1 | antithrombin-III | gi\|363736402 | *Gallus gallus* | 9 | 29 | 0.203 | 100 | 1.234 | 0.001 | ↑ |
| HPX | hemopexin | gi\|16805334 | *Gallus gallus* | 4 | 7 | 0.157 | 100 | 1.226 | 0.048 | ↑ |
| XPNPEP3 | probable Xaa-Pro aminopeptidase 3 | gi\|50728694 | *Gallus gallus* | 3 | 9 | 0.055 | 100 | 1.204 | 0.008 | ↑ |
| EIF4H | eukaryotic translation initiation factor 4H | gi\|124249410 | *Gallus gallus* | 4 | 11 | 0.425 | 100 | 0.8 | 0.014 | ↓ |
| GC | vitamin D-binding protein precursor | gi\|45382425 | *Gallus gallus* | 11 | 45 | 0.277 | 100 | 0.79 | 0.001 | ↓ |
| HNRNPAB | heterogeneous nuclear ribonucleoprotein A/B | gi\|45384514 | *Gallus gallus* | 11 | 22 | 0.616 | 100 | 0.79 | 0.001 | ↓ |
| NFU1 | hypothetical protein RCJMB04_35n21 | gi\|53136898 | *Gallus gallus* | 3 | 5 | 0.155 | 100 | 0.79 | 0.026 | ↓ |
| SFPQ | splicing factor, proline- and glutamine-rich | gi\|363742292 | *Gallus gallus* | 15 | 29 | 0.332 | 100 | 0.789 | 0.001 | ↓ |
| CHP1 | calcineurin B homologous protein 1 | gi\|56118996 | *Gallus gallus* | 4 | 8 | 0.272 | 100 | 0.785 | 0.008 | ↓ |
| LGALS2 | galectin-2-like isoform 1 | gi\|363727877 | *Gallus gallus* | 3 | 7 | 0.248 | 100 | 0.782 | 0.008 | ↓ |
| PABPC1 | polyadenylate-binding protein 1 | gi\|71896197 | *Gallus gallus* | 12 | 42 | 0.316 | 100 | 0.777 | 0.001 | ↓ |
| SYNCRIP | synaptotagmin binding, cytoplasmic RNA interacting protein | gi\|71894995 | *Gallus gallus* | 8 | 19 | 0.326 | 100 | 0.777 | 0.001 | ↓ |
| FAM222B | uncharacterized protein LOC417578 | gi\|71897055 | *Gallus gallus* | 1 | 7 | 0.029 | 100 | 0.775 | 0.023 | ↓ |
| CCT2 | T-complex protein 1 subunit beta | gi\|60302718 | *Gallus gallus* | 20 | 87 | 0.505 | 100 | 0.774 | 0.001 | ↓ |
| PSMD9 | 26S proteasome non-ATPase regulatory subunit 9 | gi\|57525182 | *Gallus gallus* | 3 | 4 | 0.164 | 100 | 0.766 | 0.043 | ↓ |
| PSMF1 | proteasome inhibitor PI31 subunit | gi\|71896121 | *Gallus gallus* | 4 | 7 | 0.173 | 100 | 0.764 | 0.005 | ↓ |
| PFDN2 | prefoldin subunit 2-like | gi\|363742957 | *Gallus gallus* | 5 | 12 | 0.321 | 100 | 0.758 | 0.002 | ↓ |
| CSDE1 | cold shock domain-containing protein E1 | gi\|71896273 | *Gallus gallus* | 9 | 19 | 0.135 | 100 | 0.756 | 0.001 | ↓ |
| SUGT1 | suppressor of G2 allele of SKP1 homolog | gi\|302486333 | *Gallus gallus* | 4 | 7 | 0.168 | 99.98 | 0.754 | 0.005 | ↓ |
| TCERG1 | uncharacterized protein LOC416349 | gi\|363739271 | *Gallus gallus* | 7 | 9 | 0.064 | 100 | 0.745 | 0.014 | ↓ |
| RBMX | heterogeneous nuclear ribonucleoprotein G | gi\|119331082 | *Gallus gallus* | 4 | 10 | 0.112 | 100 | 0.744 | 0.001 | ↓ |
| FSCN1 | fascin | gi\|296011017 | *Gallus gallus* | 6 | 10 | 0.163 | 100 | 0.734 | 0.013 | ↓ |
| PBDC1 | hypothetical protein RCJMB04_33c16 | gi\|53136736 | *Gallus gallus* | 3 | 4 | 0.182 | 100 | 0.732 | 0.022 | ↓ |
| HNRNPA1 | heterogeneous nuclear ribonucleoprotein A1 | gi\|71895177 | *Gallus gallus* | 9 | 13 | 0.654 | 100 | 0.718 | 0.003 | ↓ |
| ADD3 | hypothetical protein RCJMB04_6m11 | gi\|60098711 | *Gallus gallus* | 4 | 7 | 0.078 | 100 | 0.716 | 0.005 | ↓ |
| SCUBE2 | signal peptide, CUB domain, EGF-like 2 | gi\|363734260 | *Gallus gallus* | 1 | 7 | 0.008 | 100 | 0.714 | 0.001 | ↓ |
| SH3BGRL | SH3 domain-binding glutamic acid-rich-like protein | gi\|60302796 | *Gallus gallus* | 5 | 10 | 0.614 | 100 | 0.71 | 0.002 | ↓ |
| SNW1 | SNW domain-containing protein 1 | gi\|50748542 | *Gallus gallus* | 2 | 4 | 0.035 | 99.85 | 0.703 | 0.016 | ↓ |
| SSB | Sjogren syndrome antigen B (autoantigen La) isoform 1 | gi\|302488427 | *Gallus gallus* | 5 | 13 | 0.139 | 100 | 0.689 | 0.001 | ↓ |
| CA5B | carbonic anhydrase 5B, mitochondrial | gi\|363738234 | *Gallus gallus* | 3 | 7 | 0.13 | 99.09 | 0.682 | 0.036 | ↓ |
| HRG | histidine-rich glycoprotein | gi\|410610509 | *Gallus gallus* | 1 | 3 | 0.021 | 99.15 | 0.682 | 0.031 | ↓ |
| TSR1 | pre-rRNA-processing protein TSR1 homolog | gi\|363741127 | *Gallus gallus* | 2 | 4 | 0.031 | 100 | 0.681 | 0.03 | ↓ |
| SLC4A4 | electrogenic sodium bicarbonate cotransporter 1 isoform 2 | gi\|118090277 | *Gallus gallus* | 2 | 3 | 0.023 | 100 | 0.673 | 0.027 | ↓ |
| FKBP10 | peptidyl-prolyl cis-trans isomerase FKBP10-like isoform 1 | gi\|363743597 | *Gallus gallus* | 5 | 9 | 0.121 | 99.72 | 0.668 | 0.001 | ↓ |
| RCN2 | reticulocalbin-2 | gi\|118095590 | *Gallus gallus* | 1 | 4 | 0.03 | 100 | 0.66 | 0.001 | ↓ |
| AHSA1 | activator of 90 kDa heat shock protein ATPase homolog 1 isoform 2 | gi\|50748536 | *Gallus gallus* | 3 | 3 | 0.079 | 100 | 0.659 | 0.047 | ↓ |
| EIF5 | eukaryotic translation initiation factor 5 | gi\|57529904 | *Gallus gallus* | 5 | 12 | 0.123 | 100 | 0.652 | 0.004 | ↓ |
| NUMA1 | nuclear mitotic apparatus protein 1 | gi\|300116724 | *Gallus gallus* | 4 | 4 | 0.023 | 100 | 0.646 | 0.018 | ↓ |
| MCM4 | DNA replication licensing factor mcm4 | gi\|118086936 | *Gallus gallus* | 7 | 11 | 0.087 | 100 | 0.642 | 0.001 | ↓ |
| NCL | nucleolin | gi\|45384000 | *Gallus gallus* | 17 | 52 | 0.266 | 98.61 | 0.62 | 0.001 | ↓ |
| DNAJC9 | dnaJ homolog subfamily C member 9 | gi\|313747537 | *Gallus gallus* | 3 | 6 | 0.112 | 100 | 0.616 | 0.005 | ↓ |
| FETUB | fetuin-B | gi\|50752383 | *Gallus gallus* | 6 | 11 | 0.216 | 100 | 0.614 | 0.013 | ↓ |
| WDR77 | methylosome protein 50 | gi\|71895697 | *Gallus gallus* | 4 | 6 | 0.17 | 99.9 | 0.607 | 0.001 | ↓ |
| RSL1D1 | ribosomal L1 domain containing 1 | gi\|208609946 | *Gallus gallus* | 1 | 3 | 0.018 | 100 | 0.601 | 0.008 | ↓ |
| COL5A1 | collagen alpha-1(V) chain precursor | gi\|46048885 | *Gallus gallus* | 1 | 2 | 0.023 | 100 | 0.378 | 0.001 | ↓ |
| EXOSC9 | exosome complex exonuclease RRP45 | gi\|77797833 | *Gallus gallus* | 1 | 1 | 0.023 | 100 | 0.1 | 0.001 | ↓ |

Abbreviations: NCBInr Identity, Identity score of blast (NCBInr); NCBInr Accession, Matched accession of blast (NCBInr); NCBInr Description, Description of matched accession (NCBInr); Uniq_Pep_Num, Identified unique peptide number of protein; Uniq_Spec_Num, Identified unique spectrum number of protein.

**^#^** compared with control group, ↑ indicated up-regulated; ↓ indicated down-regulated.

Tendency: proteins expression changes at E19d than that at E14d in chicken embryo, ↑indicated up-regulated; ↓indicated down-regulated.
