# Supplementary material for: Proteomics reveals changes in hepatic proteins during chicken embryonic development: an alternative model to study human obesity
Source: BMC Genomics. 2018 Jan 8;19:29. doi: 10.1186/s12864-017-4427-6 (PMC5759888; doi:10.1186/s12864-017-4427-6)
Supplement: Supplementary file 6 — Differentially expressed proteins with no annotated functions at H1d when compared to E19d in chicken embryos. (DOCX 32 kb) [file 12864_2017_4427_MOESM6_ESM.docx]

**Online Additional file**

**Proteomics analysis reveals hepatic proteins changes during chicken embryonic development：An alternative model for human obesity study**

Mengling Peng, Shengnan Li, Qianqian He, Jinlong Zhao, Longlong Li, Haitian Ma*

**Additional Table 5.** Differentially expressed proteins with no annotated functions at H1d when compared to E19d in chicken embryos

| Gene Ontology | NCBInr Description | NCBInr Accession | Species | Uniq_Pep _Num | Uniq_Spec_Num | Protein Coverage | NCBInr Identity | Ratio | P-value | Tendency |
| --- | --- | --- | --- | --- | --- | --- | --- | --- | --- | --- |
| IGLL1 | Ig light chain precursor | gi\|212195 | *Gallus gallus* | 1 | 17 | 0.194 | 100 | 1.954 | 0.001 | ↑ |
| XPNPEP3 | probable Xaa-Pro aminopeptidase 3 | gi\|50728694 | *Gallus gallus* | 3 | 9 | 0.055 | 100 | 1.794 | 0.004 | ↑ |
| A2ML4 | alpha-2-macroglobulin-like protein 1-like | gi\|363743392 | *Gallus gallus* | 16 | 26 | 0.135 | 99.72 | 1.592 | 0.001 | ↑ |
| SLC26A5 | prestin | gi\|118405150 | *Gallus gallus* | 2 | 4 | 0.044 | 100 | 1.578 | 0.022 | ↑ |
| COX7C | cytochrome c oxidase subunit 7C, mitochondrial-like | gi\|118104378 | *Gallus gallus* | 1 | 4 | 0.143 | 100 | 1.556 | 0.001 | ↑ |
| MRPS36 | 8S ribosomal protein S36, mitochondrial | gi\|363744215 | *Gallus gallus* | 1 | 7 | 0.126 | 100 | 1.517 | 0.004 | ↑ |
| SCUBE2 | signal peptide, CUB domain, EGF-like 2 | gi\|363734260 | *Gallus gallus* | 1 | 7 | 0.008 | 100 | 1.498 | 0.001 | ↑ |
| MYDGF | uncharacterized protein LOC420161 precursor | gi\|57530610 | *Gallus gallus* | 1 | 3 | 0.054 | 100 | 1.471 | 0.005 | ↑ |
| GFM1 | elongation factor G, mitochondrial | gi\|118095339 | *Gallus gallus* | 9 | 25 | 0.14 | 100 | 1.436 | 0.001 | ↑ |
| TOP1 | DNA topoisomerase 1 | gi\|45384130 | *Gallus gallus* | 2 | 4 | 0.024 | 100 | 1.415 | 0.034 | ↑ |
| SSB | Sjogren syndrome antigen B (autoantigen La) isoform 1 | gi\|302488427 | *Gallus gallus* | 5 | 13 | 0.139 | 99.75 | 1.369 | 0.004 | ↑ |
| SLC9A3R2 | Na(+)/H(+) exchange regulatory cofactor NHE-RF2 | gi\|363739404 | *Gallus gallus* | 2 | 5 | 0.093 | 100 | 1.33 | 0.013 | ↑ |
| COMMD6 | COMM domain-containing protein 6 | gi\|363729141 | *Gallus gallus* | 2 | 4 | 0.135 | 100 | 1.277 | 0.039 | ↑ |
| SF3B1 | splicing factor 3B subunit 1 | gi\|363735880 | *Gallus gallus* | 4 | 11 | 0.037 | 100 | 1.273 | 0.011 | ↑ |
| OVALY | ovalbumin-related protein Y | gi\|71897377 | *Gallus gallus* | 7 | 13 | 0.229 | 100 | 1.268 | 0.031 | ↑ |
| PRDX1 | peroxiredoxin-1 | gi\|429836849 | *Gallus gallus* | 9 | 97 | 0.724 | 100 | 1.265 | 0.001 | ↑ |
| PRMT5 | hypothetical protein RCJMB04_14b8 | gi\|53132882 | *Gallus gallus* | 4 | 5 | 0.076 | 100 | 1.252 | 0.029 | ↑ |
| C11ORF54 | chromosome 1 open reading frame, human C11orf54 | gi\|471434827 | *Gallus gallus* | 10 | 37 | 0.5 | 100 | 1.243 | 0.001 | ↑ |
| EFHD1 | EF-hand domain-containing protein D1 | gi\|72535161 | *Gallus gallus* | 3 | 9 | 0.151 | 100 | 1.243 | 0.011 | ↑ |
| YVCT | probable 2-ketogluconate reductase-like, partial | gi\|363745151 | *Gallus gallus* | 2 | 7 | 0.195 | 100 | 1.24 | 0.013 | ↑ |
| PABPC1 | polyadenylate-binding protein 1 | gi\|71896197 | *Gallus gallus* | 12 | 42 | 0.316 | 100 | 1.233 | 0.001 | ↑ |
| MRPL40 | 39S ribosomal protein L40, mitochondrial-like | gi\|363739973 | *Gallus gallus* | 5 | 8 | 0.337 | 100 | 1.226 | 0.026 | ↑ |
| PLG | plasminogen | gi\|118088308 | *Gallus gallus* | 9 | 13 | 0.132 | 100 | 1.216 | 0.021 | ↑ |
| TOMM70A | mitochondrial import receptor subunit TOM70 [Gallus gallus] | gi\|50729660 | *Gallus gallus* | 8 | 13 | 0.139 | 100 | 1.212 | 0.011 | ↑ |
| MANF | putative RNA-binding protein 15B | gi\|363738498 | *Gallus gallus* | 5 | 10 | 0.383 | 100 | 1.211 | 0.001 | ↑ |
| AKR1B10 | aldo-keto reductase family 1 member B10 | gi\|45382879 | *Gallus gallus* | 1 | 8 | 0.069 | 100 | 0.79 | 0.002 | ↓ |
| SLC25A20 | mitochondrial carnitine/acylcarnitine carrier protein | gi\|50754473 | *Gallus gallus* | 7 | 30 | 0.296 | 100 | 0.785 | 0.001 | ↓ |
| BPIFB2 | ovoglobulinG2 type AA | gi\|385145527 | *Gallus gallus* | 1 | 8 | 0.109 | 100 | 0.784 | 0.018 | ↓ |
| NLN | neurolysin, mitochondrial | gi\|118103869 | *Gallus gallus* | 3 | 5 | 0.046 | 100 | 0.779 | 0.011 | ↓ |
| CO1A2 | Collagen alpha-2(I) chain | gi\|5921192 | *Gallus gallus* | 9 | 15 | 0.112 | 100 | 0.766 | 0.01 | ↓ |
| SCARB2 | lysosome membrane protein 2 | gi\|50746651 | *Gallus gallus* | 9 | 31 | 0.249 | 100 | 0.756 | 0.001 | ↓ |
| P22 | calcium-binding protein | gi\|46048671 | *Gallus gallus* | 5 | 27 | 0.26 | 100 | 0.754 | 0.001 | ↓ |
| COMTD1 | catechol O-methyltransferase domain-containing protein 1 | gi\|363735497 | *Gallus gallus* | 5 | 8 | 0.199 | 100 | 0.739 | 0.012 | ↓ |
| GPD1L | glycerol-3-phosphate dehydrogenase 1-like | gi\|50732786 | *Gallus gallus* | 2 | 16 | 0.076 | 100 | 0.737 | 0.001 | ↓ |
| CATH1 | fowlicidin-1 | gi\|72003802 | *Gallus gallus* | 2 | 4 | 0.196 | 99.32 | 0.733 | 0.007 | ↓ |
| AvBD1 | gallinacin-1 | gi\|50404774 | *Gallus gallus* | 1 | 3 | 0.205 | 100 | 0.696 | 0.038 | ↓ |
| CAPN11 | CAPN1 | gi\|209892841 | *Gallus gallus* | 2 | 4 | 0.044 | 100 | 0.684 | 0.005 | ↓ |
| SPINK7 | ovomucoid | gi\|209979542 | *Gallus gallus* | 2 | 5 | 0.157 | 100 | 0.676 | 0.005 | ↓ |
| MELTF | melanotransferrin precursor | gi\|45383930 | *Gallus gallus* | 7 | 18 | 0.13 | 99.05 | 0.669 | 0.001 | ↓ |
| FBXL12 | hepatic lectin | gi\|45382743 | *Gallus gallus* | 2 | 9 | 0.068 | 100 | 0.658 | 0.001 | ↓ |
| LYG2 | lysozyme g precursor | gi\|47825389 | *Gallus gallus* | 5 | 7 | 0.299 | 99.53 | 0.657 | 0.021 | ↓ |
| RSPRY1 | RING finger and SPRY domain-containing protein 1 | gi\|50753502 | *Gallus gallus* | 1 | 3 | 0.014 | 100 | 0.655 | 0.023 | ↓ |
| MOGAT1 | 2-acylglycerol O-acyltransferase 1 | gi\|363737112 | *Gallus gallus* | 5 | 15 | 0.221 | 99.67 | 0.643 | 0.001 | ↓ |
| M126 | Protein MRP-126 | gi\|126659 | *Gallus gallus* | 2 | 2 | 0.143 | 100 | 0.637 | 0.049 | ↓ |
| TTN | connectin/titin | gi\|1513030 | *Gallus gallus* | 1 | 5 | 0.005 | 88.65 | 0.522 | 0.002 | ↓ |
| HELZ2 | peroxisomal proliferator-activated receptor A-interacting complex 285 kDa protein | gi\|118100744 | *Gallus gallus* | 2 | 4 | 0.006 | 100 | 0.327 | 0.03 | ↓ |

Abbreviations: NCBInr Identity, Identity score of blast (NCBInr); NCBInr Accession, Matched accession of blast (NCBInr); NCBInr Description, Description of matched accession (NCBInr); Uniq_Pep_Num, Identified unique peptide number of protein; Uniq_Spec_Num, Identified unique spectrum number of protein.

**^#^** compared with control group, ↑ indicated up-regulated; ↓ indicated down-regulated.

Tendency: proteins expression changes at E19d than that at E14d in chicken embryo, ↑indicated up-regulated; ↓indicated down-regulated.
