# Supplementary material for: Proteomics reveals changes in hepatic proteins during chicken embryonic development: an alternative model to study human obesity
Source: BMC Genomics. 2018 Jan 8;19:29. doi: 10.1186/s12864-017-4427-6 (PMC5759888; doi:10.1186/s12864-017-4427-6)
Supplement: Supplementary file 7 — Detailed pathway enrichment of differential expression protein between E14d and E19d in chicken embryos. (DOCX 22 kb) [file 12864_2017_4427_MOESM7_ESM.docx]

**Online Additional file**

**Proteomics analysis reveals hepatic proteins changes during chicken embryonic development：An alternative model for human obesity study**

Mengling Peng, Shengnan Li, Qianqian He, Jinlong Zhao, Longlong Li, Haitian Ma*

**Additional Table 6.** Detailed pathway enrichment of differential expression protein between E14d and E19d in chicken embryos

| Pathway Description | Observed Protein Count | False Discovery Rate | Matching Proteins in Pathway Network |
| --- | --- | --- | --- |
| Metabolic pathways | 56 | 7.64E-22 | ACAA2, ACOX1, ACSL1, ACSS1, ADI1, AGPAT3, AGXT2L1, AKR1A1, AKR1D1, ALDH3A2, ALDH7A1, ALDH9A1, AMACR, ATP5F1, ATP5H, AUH, BDH1, BDH2, CBR1, COX4I1, CTH, DDOST, DUT, EHHADH, ENOPH1, FBP1, FBP2, FTCD, GATM, GLUL, GPI, GRHPR, GSTZ1, HMBS, HMGCS1, HPGDS, HSD17B4, IMPDH2, KMO, LDHA, MAOA, MAT1A, NDUFA9, NDUFB6, NDUFV1, NME2, NNT, NT5C2, NT5C3L, PAH, PAPSS1, PGM1, PGM2, PSAT1, RPN2, SCP2 |
| Glycolysis / Gluconeogenesis | 11 | 1.34E-10 | ACSS1, AKR1A1, ALDH3A2, ALDH7A1, ALDH9A1, FBP1, FBP2, GPI, LDHA, PGM1, PGM2 |
| Fatty acid degradation | 9 | 2.97E-09 | ACAA2, ACOX1, ACSL1, ALDH3A2, ALDH7A1, ALDH9A1, CPT1A, ECI2, EHHADH |
| Valine, leucine and isoleucine degradation | 8 | 7.06E-07 | ACAA2, ALDH3A2, ALDH7A1, ALDH9A1, AUH, EHHADH, HMGCS1, OXCT1 |
| Protein processing in endoplasmic reticulum | 11 | 1.28E-05 | CALR3, CKAP4, DDOST, ERP29, HYOU1, LMAN1, P4HB, PDIA4, RPN2, TXNDC5, UBQLN1 |
| Pentose phosphate pathway | 5 | 2.97E-05 | FBP1, FBP2, GPI, PGM1, PGM2 |
| Glycine, serine and threonine metabolism | 6 | 2.97E-05 | ALDH7A1, CTH, GATM, GRHPR, MAOA, PSAT1 |
| Pyruvate metabolism | 6 | 4.05E-05 | ACSS1, ALDH3A2, ALDH7A1, ALDH9A1, GRHPR, LDHA |
| Butanoate metabolism | 5 | 5.90E-05 | BDH1, BDH2, EHHADH, HMGCS1, OXCT1 |
| Arginine and proline metabolism | 6 | 0.00018 | ALDH3A2, ALDH7A1, ALDH9A1, GATM, GLUL, MAOA |
| Biosynthesis of amino acids | 6 | 0.000298 | ALDH7A1, CTH, GLUL, MAT1A, PAH, PSAT1 |
| Cysteine and methionine metabolism | 5 | 0.000323 | ADI1, CTH, ENOPH1,LDHA,MAT1A |
| Ribosome | 8 | 0.000375 | MRPS6, RPL10A, RPL24, RPL27A, RPL31, RPL7A, RPLP1, RPS7 |
| PPAR signaling pathway | 6 | 0.000395 | ACOX1, ACSL1, CPT1A, EHHADH, FABP5, SCP2 |
| Fatty acid metabolism | 5 | 0.00102 | ACAA2, ACOX1, ACSL1, CPT1A, EHHADH |
| beta-Alanine metabolism | 4 | 0.00103 | ALDH3A2, ALDH7A1, ALDH9A1, EHHADH |
| Glycerolipid metabolism | 5 | 0.00155 | AGPAT3, AKR1A1, ALDH3A2, ALDH7A1, ALDH9A1 |
| Carbon metabolism | 6 | 0.00232 | ACSS1, EHHADH, FBP1, FBP2, GPI, PSAT1 |
| Purine metabolism | 7 | 0.00647 | IMPDH2, NME2, NT5C2, NT5C3L, PAPSS1, PGM1, PGM2 |
| Amino sugar and nucleotide sugar metabolism | 4 | 0.00661 | CYB5R2, GPI, PGM1, PGM2 |
| Oxidative phosphorylation | 6 | 0.00993 | ATP5F1, ATP5H, COX4I1, NDUFA9, NDUFB6, NDUFV1 |
