# Supplementary material for: Proteomics reveals changes in hepatic proteins during chicken embryonic development: an alternative model to study human obesity
Source: BMC Genomics. 2018 Jan 8;19:29. doi: 10.1186/s12864-017-4427-6 (PMC5759888; doi:10.1186/s12864-017-4427-6)
Supplement: Supplementary file 8 — Detailed pathway enrichment of differential expression protein between E19d and H1d in chicken embryos. (DOCX 22 kb) [file 12864_2017_4427_MOESM8_ESM.docx]

**Online additional file**

**Proteomics analysis reveals hepatic proteins changes during chicken embryonic development：An alternative model for human obesity study**

Mengling Peng, Shengnan Li, Qianqian He, Jinlong Zhao, Longlong Li, Haitian Ma*

**Additional Table 7.** Detailed pathway enrichment of differential expression protein between E19d and H1d in chicken embryos

| Pathway Description | Observed Protein Count | False Discovery Rate | Matching Proteins in Pathway Network |
| --- | --- | --- | --- |
| Metabolic pathways | 33 | 3.45E-11 | ABAT, ACOX1, ACSL5, ADSL, AGPAT3, AKR1A1, AKR1D1, ATP5H, ATP8, COX7C, CYCS, FASN, GAPDH, GATM, GLDC, GNE, HAL, HMGCS1, HPD, IMPDH2, ITPK1, LDHA, LDHB, NT5C2, OGDH, PAICS, PGM2, PPAT, SHMT1, UGP2, UQCRFS1, XDH |
| Glycolysis / Gluconeogenesis | 5 | 0.00112 | AKR1A1, GAPDH, LDHA, LDHB, PGM2 |
| Purine metabolism | 7 | 0.00327 | ADSL, IMPDH2, NT5C2, PAICS, PGM2, PPAT, XDH |
| Ribosome | 6 | 0.00527 | RPL18A, RPL19, RPL7A, RPLP1, RPS2, RPS3A |
| Pentose and glucuronate interconversions | 2 | 0.00685 | AKR1A1, UGP2 |
| PPAR signaling pathway | 4 | 0.0172 | ACOX1, ACSL5, DBI, FABP1 |
| Galactose metabolism | 3 | 0.0195 | PGM2, UGP2 |
| Propanoate metabolism | 3 | 0.0197 | ABAT, LDHA, LDHB |
| Alanine, aspartate and glutamate metabolism | 3 | 0.0247 | ABAT, ADSL, PPAT |
| Glycine, serine and threonine metabolism | 3 | 0.0247 | GATM, GLDC, SHMT1 |
| Pyruvate metabolism | 2 | 0.0297 | LDHA, LDHB |
| Amino sugar and nucleotide sugar metabolism | 3 | 0.0355 | GNE, PGM2, UGP2 |
| Carbon metabolism | 4 | 0.0355 | GAPDH, GLDC, OGDH, SHMT1 |
| Protein processing in endoplasmic reticulum | 5 | 0.0377 | ATXN3, CALR3, ERP29, HSPA5, HSPH1 |
| Fatty acid metabolism | 3 | 0.039 | ACOX1, ACSL5, FASN |
| Phenylalanine metabolism | 2 | 0.0446 | HPD, MIF |
| Glycerolipid metabolism | 3 | 0.0464 | AGPAT3, AKR1A1 |
| Nitrogen metabolism | 1 | 0.0464 | CA2 |
