# Supplementary material for: Proteomics reveals changes in hepatic proteins during chicken embryonic development: an alternative model to study human obesity
Source: BMC Genomics. 2018 Jan 8;19:29. doi: 10.1186/s12864-017-4427-6 (PMC5759888; doi:10.1186/s12864-017-4427-6)
Supplement: Supplementary file 10 — Differentially expressed proteins which were not assigned to any known functions at H1d when compared to E19d in chicken embryos. (DOCX 28 kb) [file 12864_2017_4427_MOESM10_ESM.docx]

**Online Additional file**

**Proteomics analysis reveals hepatic proteins changes during chicken embryonic development：An alternative model for human obesity study**

Mengling Peng, Shengnan Li, Qianqian He, Jinlong Zhao, Longlong Li, Haitian Ma*

**Additional Table 9.** Differentially expressed proteins which were not assigned to any known functions at H1d when compared to E19d in chicken embryos

| Gene Ontology | NCBInr Description | NCBInr Accession | Species | Uniq_Pep _Num | Uniq_Spec_Num | Protein Coverage | NCBInr Identity | Ratio | P-value | Tendency |
| --- | --- | --- | --- | --- | --- | --- | --- | --- | --- | --- |
| IGLL1 | Ig light chain precursor | gi\|212195 | *Gallus gallus* | 1 | 17 | 0.194 | 100 | 1.954 | 0.001 | ↑ |
| A2ML4 | alpha-2-macroglobulin-like protein 1-like | gi\|363743392 | *Gallus gallus* | 16 | 26 | 0.135 | 99.72 | 1.592 | 0.001 | ↑ |
| SCUBE2 | signal peptide, CUB domain, EGF-like 2 | gi\|363734260 | *Gallus gallus* | 1 | 7 | 0.008 | 100 | 1.498 | 0.001 | ↑ |
| MYDGF | uncharacterized protein LOC420161 precursor | gi\|57530610 | *Gallus gallus* | 1 | 3 | 0.054 | 100 | 1.471 | 0.005 | ↑ |
| SLC9A3R2 | Na(+)/H(+) exchange regulatory cofactor NHE-RF2 | gi\|363739404 | *Gallus gallus* | 2 | 5 | 0.093 | 100 | 1.33 | 0.013 | ↑ |
| COMMD6 | COMM domain-containing protein 6 | gi\|363729141 | *Gallus gallus* | 2 | 4 | 0.135 | 100 | 1.277 | 0.039 | ↑ |
| OVALY | ovalbumin-related protein Y | gi\|71897377 | *Gallus gallus* | 7 | 13 | 0.229 | 100 | 1.268 | 0.031 | ↑ |
| PRMT5 | hypothetical protein RCJMB04_14b8 | gi\|53132882 | *Gallus gallus* | 4 | 5 | 0.076 | 100 | 1.252 | 0.029 | ↑ |
| C11ORF54 | chromosome 1 open reading frame, human C11orf54 | gi\|471434827 | *Gallus gallus* | 10 | 37 | 0.5 | 100 | 1.243 | 0.001 | ↑ |
| EFHD1 | EF-hand domain-containing protein D1 | gi\|72535161 | *Gallus gallus* | 3 | 9 | 0.151 | 100 | 1.243 | 0.011 | ↑ |
| YVCT | probable 2-ketogluconate reductase-like, partial | gi\|363745151 | *Gallus gallus* | 2 | 7 | 0.195 | 100 | 1.24 | 0.013 | ↑ |
| MANF | putative RNA-binding protein 15B | gi\|363738498 | *Gallus gallus* | 5 | 10 | 0.383 | 100 | 1.211 | 0.001 | ↑ |
| AKR1B10 | aldo-keto reductase family 1 member B10 | gi\|45382879 | *Gallus gallus* | 1 | 8 | 0.069 | 100 | 0.79 | 0.002 | ↓ |
| SLC25A20 | mitochondrial carnitine/acylcarnitine carrier protein | gi\|50754473 | *Gallus gallus* | 7 | 30 | 0.296 | 100 | 0.785 | 0.001 | ↓ |
| BPIFB2 | ovoglobulinG2 type AA | gi\|385145527 | *Gallus gallus* | 1 | 8 | 0.109 | 100 | 0.784 | 0.018 | ↓ |
| NLN | neurolysin, mitochondrial | gi\|118103869 | *Gallus gallus* | 3 | 5 | 0.046 | 100 | 0.779 | 0.011 | ↓ |
| CO1A2 | Collagen alpha-2(I) chain | gi\|5921192 | *Gallus gallus* | 9 | 15 | 0.112 | 100 | 0.766 | 0.01 | ↓ |
| P22 | calcium-binding protein | gi\|46048671 | *Gallus gallus* | 5 | 27 | 0.26 | 100 | 0.754 | 0.001 | ↓ |
| COMTD1 | catechol O-methyltransferase domain-containing protein 1 | gi\|363735497 | *Gallus gallus* | 5 | 8 | 0.199 | 100 | 0.739 | 0.012 | ↓ |
| CATH1 | fowlicidin-1 | gi\|72003802 | *Gallus gallus* | 2 | 4 | 0.196 | 99.32 | 0.733 | 0.007 | ↓ |
| AvBD1 | gallinacin-1 | gi\|50404774 | *Gallus gallus* | 1 | 3 | 0.205 | 100 | 0.696 | 0.038 | ↓ |
| CAPN11 | CAPN1 | gi\|209892841 | *Gallus gallus* | 2 | 4 | 0.044 | 100 | 0.684 | 0.005 | ↓ |
| SPINK7 | ovomucoid | gi\|209979542 | *Gallus gallus* | 2 | 5 | 0.157 | 100 | 0.676 | 0.005 | ↓ |
| MELTF | melanotransferrin precursor | gi\|45383930 | *Gallus gallus* | 7 | 18 | 0.13 | 99.05 | 0.669 | 0.001 | ↓ |
| FBXL12 | hepatic lectin | gi\|45382743 | *Gallus gallus* | 2 | 9 | 0.068 | 100 | 0.658 | 0.001 | ↓ |
| RSPRY1 | RING finger and SPRY domain-containing protein 1 | gi\|50753502 | *Gallus gallus* | 1 | 3 | 0.014 | 100 | 0.655 | 0.023 | ↓ |
| M126 | Protein MRP-126 | gi\|126659 | *Gallus gallus* | 2 | 2 | 0.143 | 100 | 0.637 | 0.049 | ↓ |
| TTN | connectin/titin | gi\|1513030 | *Gallus gallus* | 1 | 5 | 0.005 | 88.65 | 0.522 | 0.002 | ↓ |

Abbreviations: NCBInr Identity, Identity score of blast (NCBInr); NCBInr Accession, Matched accession of blast (NCBInr); NCBInr Description, Description of matched accession (NCBInr); Uniq_Pep_Num, Identified unique peptide number of protein; Uniq_Spec_Num, Identified unique spectrum number of protein.

**^#^** compared with control group, ↑ indicated up-regulated; ↓ indicated down-regulated.

Tendency: proteins expression changes at E19d than that at E14d in chicken embryo, ↑indicated up-regulated; ↓indicated down-regulated.
